# A novel multistage antiplasmodial inhibitor targeting *Plasmodium falciparum* histone deacetylase 1

**DOI:** 10.1038/s41421-020-00215-4

**Published:** 2020-12-11

**Authors:** Zhenghui Huang, Ruoxi Li, Tongke Tang, Dazheng Ling, Manjiong Wang, Dandan Xu, Maoxin Sun, Lulu Zheng, Feng Zhu, Hui Min, Rachasak Boonhok, Yan Ding, Yuhao Wen, Yicong Chen, Xiaokang Li, Yuxi Chen, Taiping Liu, Jiping Han, Jun Miao, Qiang Fang, Yaming Cao, Yun Tang, Jie Cui, Wenyue Xu, Liwang Cui, Jin Zhu, Gary Wong, Jian Li, Lubin Jiang

**Affiliations:** 1 Key Laboratory of Molecular Virology and Immunology, Institut Pasteur of Shanghai, University of Chinese Academy of Sciences, Chinese Academy of Sciences, Shanghai 200031, China; 2 State Key Laboratory of Bioreactor Engineering, East China University of Science and Technology, 130 Mei Long Road, Shanghai 200237, China; 3 Shanghai Key Laboratory of New Drug Design, School of Pharmacy, East China University of Science and Technology, 130 Mei Long Road, Shanghai 200237, China; 4 School of Life Science and Technology, ShanghaiTech University, Shanghai 201210, China; 5 Department of Microbiology and Parasitology, Bengbu Medical College, and Anhui Key Laboratory of Infection and Immunity, Bengbu, Anhui 233030 China; 6 Division of Infectious Diseases and International Medicine, Department of Internal Medicine, Morsani College of Medicine, University of South Florida, Tampa, FL USA; 7 Department of Pathogenic Biology, Army Medical University, Chongqing 400038, China; 8 Department of Immunology, College of Basic Medical Sciences, China Medical University, Shenyang, Liaoning 110122 China

**Keywords:** Gene silencing, Acetylation

## Abstract

Although artemisinin combination therapies have succeeded in reducing the global burden of malaria, multidrug resistance of the deadliest malaria parasite, *Plasmodium falciparum*, is emerging worldwide. Innovative antimalarial drugs that kill all life-cycle stages of malaria parasites are urgently needed. Here, we report the discovery of the compound JX21108 with broad antiplasmodial activity against multiple life-cycle stages of malaria parasites. JX21108 was developed from chemical optimization of quisinostat, a histone deacetylase inhibitor. We identified *P. falciparum* histone deacetylase 1 (PfHDAC1), an epigenetic regulator essential for parasite growth and invasion, as a molecular target of JX21108. *PfHDAC1* knockdown leads to the downregulation of essential parasite genes, which is highly consistent with the transcriptomic changes induced by JX21108 treatment. Collectively, our data support that PfHDAC1 is a potential drug target for overcoming multidrug resistance and that JX21108 treats malaria and blocks parasite transmission simultaneously.

## Introduction

There are ~200 million clinical infections and 0.4 million deaths from malaria annually, mainly among children and pregnant women in Africa^1^. At present, no effective malaria vaccines are available. In Southeast Asia and Africa, the *Plasmodium falciparum* parasite has developed resistance to all commonly used antimalarial drugs, including frontline artemisinin-based combination therapies^2,3^. Thus, the development of innovative antimalarial drugs is now urgently needed.

The majority of the current first-line antimalarial drugs target asexual blood-stage parasites^4^. Although liver-stage and gametocyte-stage parasites do not cause clinical symptoms, drugs that inhibit these two stages are essential for preventing disease epidemics and protecting vulnerable populations due to the increase in asexual antimalarial drug resistance^5–7^.

Unfortunately, almost all approved antimalarial drugs do not satisfy all these requirements for multiple life-cycle-stage antimalarial activity. While several promising advances with small molecules may achieve these important goals^8–11^, a single drug targeting all of the life-cycle stages of malaria parasites is unavailable for clinical use^12^ due to the complicated life cycle of malaria parasites. The current treatment and eradication of malaria require new antimalarials with novel mechanisms of action and without cross-resistance to the approved drugs as well as multistage antimalarial activity, especially for transmission-blocking and anti-relapse activities.

Since the discovery of the first epigenetic enzymes controlling histone acetylation for transcriptional regulation of genes, many inhibitors of epigenetic enzymes, especially histone deacetylases (HDACs), have been discovered for clinical use, mainly in cancer therapy^13,14^. Histone modifications, such as histone lysine acetylation, deacetylation, and methylation, are associated with the regulation of key processes in the life cycle of malaria parasites^15–18^. Previous research reported that three out of five *P. falciparum* HDAC (PfHDAC) genes in the parasite genome are essential for the blood stage of the parasite^18,19^, and numerous studies have identified potent antiplasmodial HDAC inhibitors^20–30^, including dual-/multistage-targeting compounds^31–35^, suggesting the possibility of targeting PfHDACs in drug development. In addition, indirect effects on histone acetylation, deacetylation activity, and transcriptomic changes have been demonstrated^36,37^. However, direct evidence of PfHDACs as a target of these compounds is lacking.

Here, we report the discovery of the compound JX21108 as a selective inhibitor of PfHDAC1, with broad activities in killing the blood stage, liver stage, and gametocytes of malaria parasites. In particular, JX21108 inhibits the growth of asexual parasites mainly by acting on trophozoites and schizonts by repressing the expression of essential genes related to metabolic processes and erythrocyte invasion. Based on three-dimensional (3D) structure modeling of PfHDACs, PfHDAC1 was implicated as a molecular target of JX21108. We demonstrated that *PfHDAC1* knockdown (KD) led to changes in the parasite transcriptome, similar to those with JX21108 treatment. Our findings of JX21108 and its target, PfHDAC1, provide a potential application in drug discovery aimed at treating malaria and blocking its transmission.

## Results

### Discovery of a new antimalarial compound

**Fig. 1:**
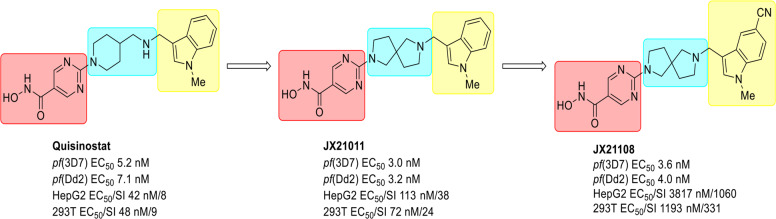
Chemical evolution of JX21108 from quisinostat. SI, selectivity index, which was calculated by the EC_50_ for HepG2 or 293T/EC_50_ for 3D7, a drug-sensitive strain of *P. falciparum*. Dd2 is another *P. falciparum* strain resistant to several antimalarial drugs, including mefloquine, pyrimethamine, and chloroquine.

To evaluate the antimalarial effect of epigenetic inhibitors, an in vitro blood-stage growth inhibition assay (GIA) against a drug-sensitive *P. falciparum* strain, 3D7, was used for phenotypic screening of a compound library synthesized by WuXi AppTec, which comprises 41 characterized inhibitors targeting HDACs, 12 targeting histone demethylases (HDMs), 2 targeting histone methyltransferases (HMTs), and 9 targeting bromodomain and extraterminal domain (BET) proteins (Supplementary Fig. S1a). Some of these compounds’ antiplasmodial activity has already been reported by previous works^35,38^. Eleven out of 64 compounds showed strong inhibitory activities on parasite growth (> 90% inhibition) at 500 nM. Among them, only five HDAC inhibitors still efficiently inhibited parasite growth at 50 nM. Furthermore, EC_50_ values of these five compounds were tested by a 3-day SYBR Green I GIA, and quisinostat showed the best antiplasmodial efficiency with an EC_50_ value of 5.2 ± 0.7 nM (Supplementary Fig. S1b). An in vivo efficacy study on a rodent malaria model with *Plasmodium yoelii* showed that only quisinostat among these five HDAC inhibitors remained effective in clearing *P. yoelii* blood-stage infections (Supplementary Fig. S1c), making quisinostat a lead compound for further research. Quisinostat is a drug candidate for the treatment of cancer, with high potency against class I and II HDACs^39,40^, and one previous research work also reported that quisinostat has antiplasmodial activity^41^. Considering the potential toxicity of quisinostat to both HepG2 and 293T cells (Fig. 1), a series of structural optimizations was conducted.

Quisinostat is constituted by three groups: a hydroxamic acid group serving as a zinc-binding group (ZBG) that chelates the Zn^2+^ cofactor in the catalytic pocket, an N-methylindole fragment (CAP region) that recognizes and interacts with the amino acid residues at the entrance of the catalytic pocket, and a pyrimidinyl 4-aminomethyl piperidine fragment (linker) connecting the two previously mentioned moieties (Fig. 1). Since hydroxamic acid is a critical pharmacophore, this domain was maintained in the structure design. Thus, the middle diamine linker was first converted into different diamines to generate 15 novel “linker” analogs (Supplementary Materials and Methods). The antiplasmodial activity of each resulting compound was evaluated by using the 3-day SYBR Green I GIA against both 3D7 and the multidrug-resistant Dd2 strain of *P. falciparum*, and cytotoxicity was also measured in HepG2 and 293T cells, among them only the compounds JX21002 (EC_50_ value of 4.4 ± 0.2 nM to 3D7 and 7.0 ± 0.2 nM to Dd2) and JX21011 (EC_50_ value of 3.0 ± 0.7 nM to 3D7 and 3.2 ± 0.1 nM to Dd2) showed similar antiplasmodial activities with attenuated cytotoxicity (selectivity index is ~24–70) compared to quisinostat (EC_50_ value of 5.2 ± 1.6 nM to 3D7, 7.09 ± 0.01 nM to Dd2 and selectivity index is ~8–9) (Supplementary Tables S1 and S2). Since the linker of JX21002 has already been protected by a patent^42^, compounds with new structure prototypes were preferentially selected for further chemical discovery. Thus, JX21011, bearing a 2,7-diazaspiro [4.4] nonane moiety, was chosen as a new lead compound (Fig. 1 and Supplementary Table S1). Further modification focusing on fine-tuning the aromatic moiety resulted in 39 analogs (Supplementary Materials and Methods). Among these, several compounds including JX21108 showed similar in vitro antiplasmodial activities, but reduced cytotoxicity compared to JX21011 (Supplementary Table S2). Specifically, JX21108 showed similarly low EC_50_ values, 3.6 nM and 4.0 nM, against 3D7 and Dd2, respectively, with the selectivity index of ~1060 and ~331 against Hep2G and 293T cells, respectively (Fig. 1 and Supplementary Table S2). All these factors made JX21108 the optimal compound over 39 derivatives.

**Fig. 2:**
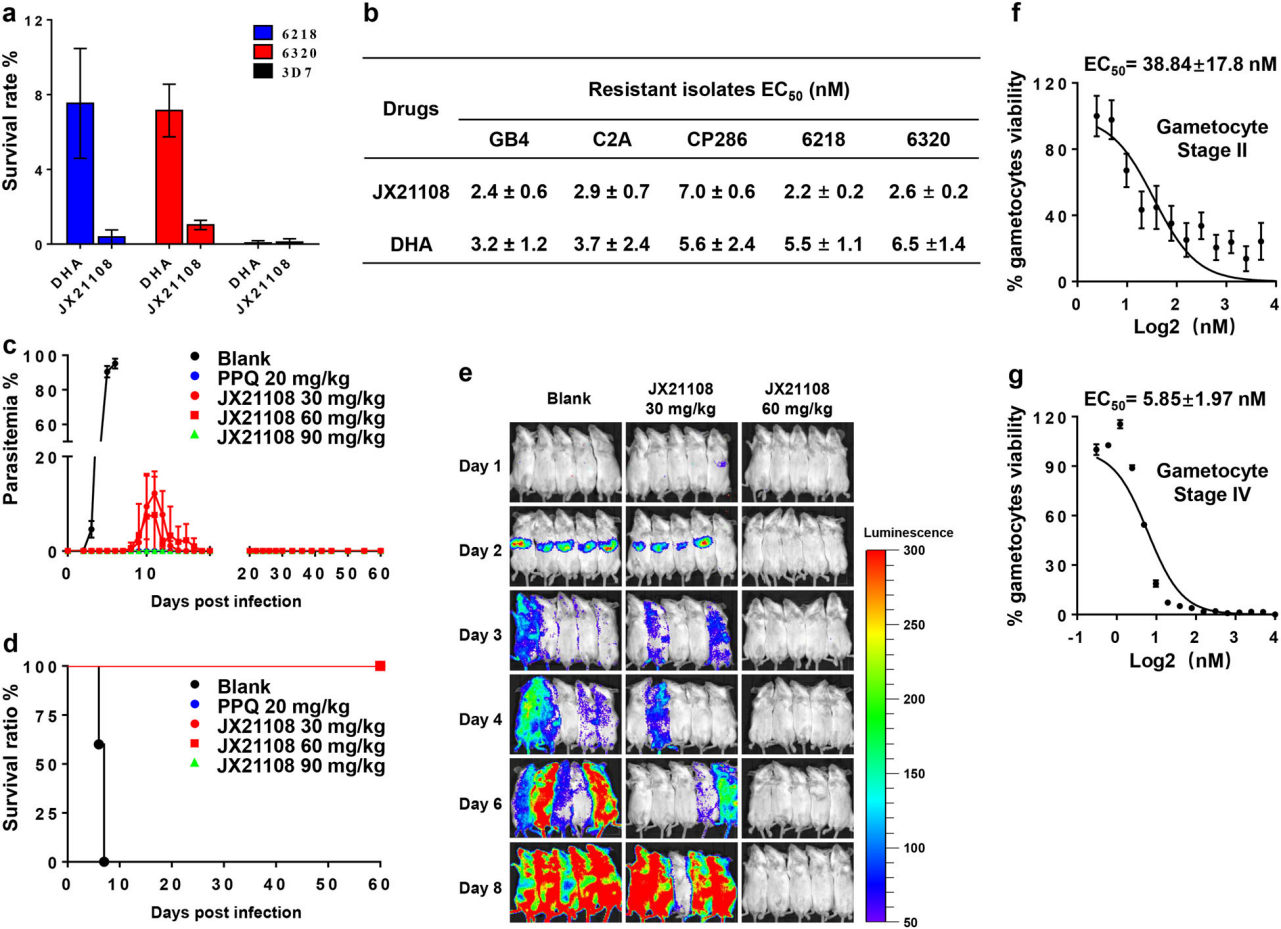
Activities of JX21108 against multiple stages of the parasite life cycle. **a** In vitro ring-stage survival assay to assess the effect of compounds on the artemisinin- and piperaquine-resistant *P. falciparum* isolates 6218 and 6320. Parasitemia was calculated from at least 10,000 RBCs by microscopy. The survival rate of the drug-exposed groups was calculated as the percentage of parasitemia compared to that of the non-drug-exposed group. The concentration of DHA and JX21108 used in this experiment was 700 nM. **b** In vitro activity against the blood stage of various *P. falciparum* strains GB4, C2A, CP286, 6218, and 6320. GB4 is resistant to chloroquine, and both C2A and CP286 are resistant to pyrimethamine and chloroquine. **c** In vivo activity against the blood stage of *P. yoelii* in BALB/c mice. Five mice were used in each group. Parasitemia was calculated from at least 5000 RBCs by microscopy. Blank, untreated group; PPQ, piperaquine phosphate. The administration route was intraperitoneal (i.p.). **d** The survival ratio of the tested mice from the experiment described in panel **c**. **e** In vivo efficacy of JX21108 against the liver stage of *P. berghei*. Mice were treated with a single dose of 30 or 60 mg/kg JX21108 by tail vein intravenous (i.v.) injection after sporozoite inoculation (i.v.) (day 0), and the infections were detected daily for 8 days by bioluminescence (five mice per group as shown). Blank, untreated group. **f**, **g** In vitro activity of JX21108 against gametocyte stages II and IV of the *P. falciparum* strain NF54. Gametocyte viability was monitored by bioluminescence. Data in **a**, **b**, **c**, **f**, and **g** are means ± SEM. Data in **a**, **b**, **f**, and **g** are from three biological replicates with duplication.

The metabolic stability of JX21108 was examined using mouse liver microsomes in vitro. The half-life of JX21108 was 12.4 h compared to 13.3 min for quisinostat, showing significant improvement in stability. In addition, JX21108 clearly exhibited interspecific differences in metabolism within rat and human liver microsomes (Table 1). Subsequently, pharmacokinetic analyses in mice indicated that after intraperitoneal injection of the tested compound at 5 mg/kg, nearly all of the tested pharmacokinetic parameters of JX21108 were better than those of quisinostat with one exception, as evidenced by the half-life values (Table 2). Although the clearance (CL) of quisinostat was higher than JX21108 in both liver microsome and in vivo pharmacokinetic assays, the in vivo half-life (T1/2) of JX21108 was still notably lower than quisinostat. Considering that quisinostat had a significantly higher apparent volume of distribution (V/F) than JX21108, we speculated that quisinostat might be widely distributed among tissues and the elimination of quisinostat in plasma might be compensated by release of quisinostat in tissues, while JX21108 might be mainly distributed and metabolized in blood. Actually, the slow release of quisinostat in tissues might also contribute to the low but detectable quisinostat (~2.5 ng/mL) in plasma during 8–12 h after administration (Table 2 and Supplementary Fig. S6). Moreover, JX21108 exhibited approximately 4-fold lower inhibitory activity on human HDACs than quisinostat (Supplementary Table S3). The improved pharmacokinetic properties including C_max_ and AUC_INF_ may have contributed to the pharmaceutical efficacy of JX21108 in *P. yoelli*-infected mice, despite the shorter half-life values (Fig. 2c, d and Table 2).

**Table 1:** In vitro drug metabolic stability of quisinostat and JX21108 in liver microsomes.

Entry	Compound	Species	*T*_1/2_^(min)^	CL (mL/min/kg)
^1^	Quisinostat	Mouse	13.31	410.10
2	JX21108	Mouse	745.27	7.32
3	JX21108	Rat	12.65	196.34
4	JX21108	Human	520.95	3.34

**Table 2:** In vivo pharmacokinetic parameters of quisinostat and JX21108 in mice.

Parameter	Quisinostat	JX21108
Dose (mg/kg)	5 (i.p.)	
*T*_max_ (h)	0.0830	0.250
*C*_max_ (ng/mL)	88.7	511
*T*_1/2_ (h)	6.13	0.926
AUC_last_ (h ng/mL)	157	432
AUC_INF_ (h ng/mL)	180	434
CL/F (L/h/kg)	27.7	11.5
V/F (L/kg)	245	15.4

As JX21108 has one stereocenter, pharmacological differences between its racemic and enantiopure forms were evaluated in the rodent malaria model. Both enantiopure antipodes of JX21108, (+)-JX21108, and (–)-JX21108, were synthesized from enantiopure starting materials obtained via chiral chromatographic separation (e.g., > 99%, Supplementary Materials and Methods). Our results showed that both enantiopure antipodes and racemic JX21108 showed similar activities regarding the parasite inhibition rate, effect on bodyweight, and survival rate of experimental animals (Fig. 2c, d; Supplementary Fig. S2a–d). Hence, racemic JX21108 would be beneficial cost-wise for antimalarial drug development, and was selected for further investigation in this study.

### The activity of JX21108 against drug-resistant *P. falciparum* isolates in vivo and *P. yoelii* in BALB/c mouse model

Having demonstrated the promising inhibitory activities of JX21108 on both drug-sensitive 3D7 and multidrug-resistant Dd2 parasites (Fig. 1 and Supplementary Table S2), we wanted to determine the effect of JX21108 on artemisinin-resistant parasites. The in vitro 3-day SYBR Green I GIA showed that JX21108 and dihydroartemisinin (DHA) have similar antiplasmodial activities against two artemisinin- and piperaquine-resistant field isolates (6218 and 6320) collected from Cambodia^43^ compared to that against 3D7 (Fig. 2b). Artemisinin resistance is manifested by delayed parasite clearance in vivo and improved survival of ring-stage parasites. Thus, a ring-stage survival assay (RSA) was performed to investigate the effect of JX21108 on the ring-stage survival of the clinical isolates 6218 and 6320. The RSA with DHA (700 nM) showed an almost 8% survival rate in the synchronized rings of the two field isolates. However, unlike DHA, JX21108 (700 nM) greatly inhibited parasite growth at ring stage of 6218 and 6320. As a positive control, both JX21108 and DHA efficiently killed the ring-stage parasites of the sensitive strain 3D7 (Fig. 2a).

JX21108 was further evaluated against different *P. falciparum* strains (GB4, C2A, and CP286) that are resistant to other first-line antimalarial drugs, as reported previously^44^. According to the standard 3-day SYBR Green I GIA, JX21108 exhibited potent inhibitory activities on parasite growth of these three strains tested with low-nanomolar EC_50_ values of 2–8 nM, in a similar range to DHA (Fig. 2b). Taken together, our results suggested that JX21108 has potent inhibitory activity against all three drug-resistant malaria parasites.

In addition, JX21108 also showed strong in vivo antiplasmodial activity in *P. yoelii*-infected mice at all doses tested (30–90 mg/kg). In the dimethyl sulfoxide (DMSO) group, the parasitemia broke out on day 2 after infection, and all mice died on day 6, while in 30 mg/kg and 60 mg/kg treatment groups, parasites were detected till day 8 and were completely cleaned up on day 17. Notably, no relapse of malaria was observed in the 90 mg/kg treatment group, and all the mice survived in the JX21108-treated group (Fig. 2c, d; Supplementary Fig. S2a). Our result strongly suggested that JX21108 can completely cure the parasite infection in the mouse model.

### Multistage activity of JX21108

We further tested the potential activity of JX21108 against other stages of the malaria parasites. To evaluate the activity of JX21108 against the liver stage, a rodent malaria model with a transgenic *P. berghei* strain expressing a reporter luciferase was utilized^45^. Two different dosages of JX21108 (30 and 60 mg/kg), presenting growth inhibition efficacy in the blood stage, were chosen for evaluation. In the control group with no drug treatment, the infection was detected and restricted to the liver at 2 days post inoculation (dpi) of *P. berghei* sporozoites by a bioluminescence assay. At a single 60 mg/kg dose, JX21108-treated mice did not show signs of liver infection at 2 dpi, nor did the treated mice show any blood-stage infection until 8 dpi (Fig. 2e). However, treatment with 30 mg/kg JX21108 did not produce significant effects on liver infection or subsequent blood-stage *P. berghei* infection (Fig. 2e).

To examine whether JX21108 has activity against gametocytes, a *P. falciparum* gametocyte producer, NF54, expressing luciferase was used^46^. Stage II and stage IV gametocytes were purified at 3 and 9 days, respectively, after induction of gametocytogenesis. Luciferase activity was monitored as an indicator of gametocyte viability. With the stage II gametocytes, JX21108 showed an EC_50_ value of 38.8 ± 17.8 nM, which was higher than its activity against the asexual blood stage (Fig. 2f). However, JX21108 displayed potent gametocytocidal activity against stage IV gametocytes with an EC_50_ value of 5.9 ± 1.97 nM (Fig. 2g). Thus, it is worth assessing the inhibition activity of JX21108 to all stages of gametocytes, especially stage V for a full assessment of transmission blocking by JX21108. Collectively, JX21108 possesses inhibitory activity against multiple life stages of malaria parasites.

### The activity of JX21108 against different blood stages

**Fig. 3:**
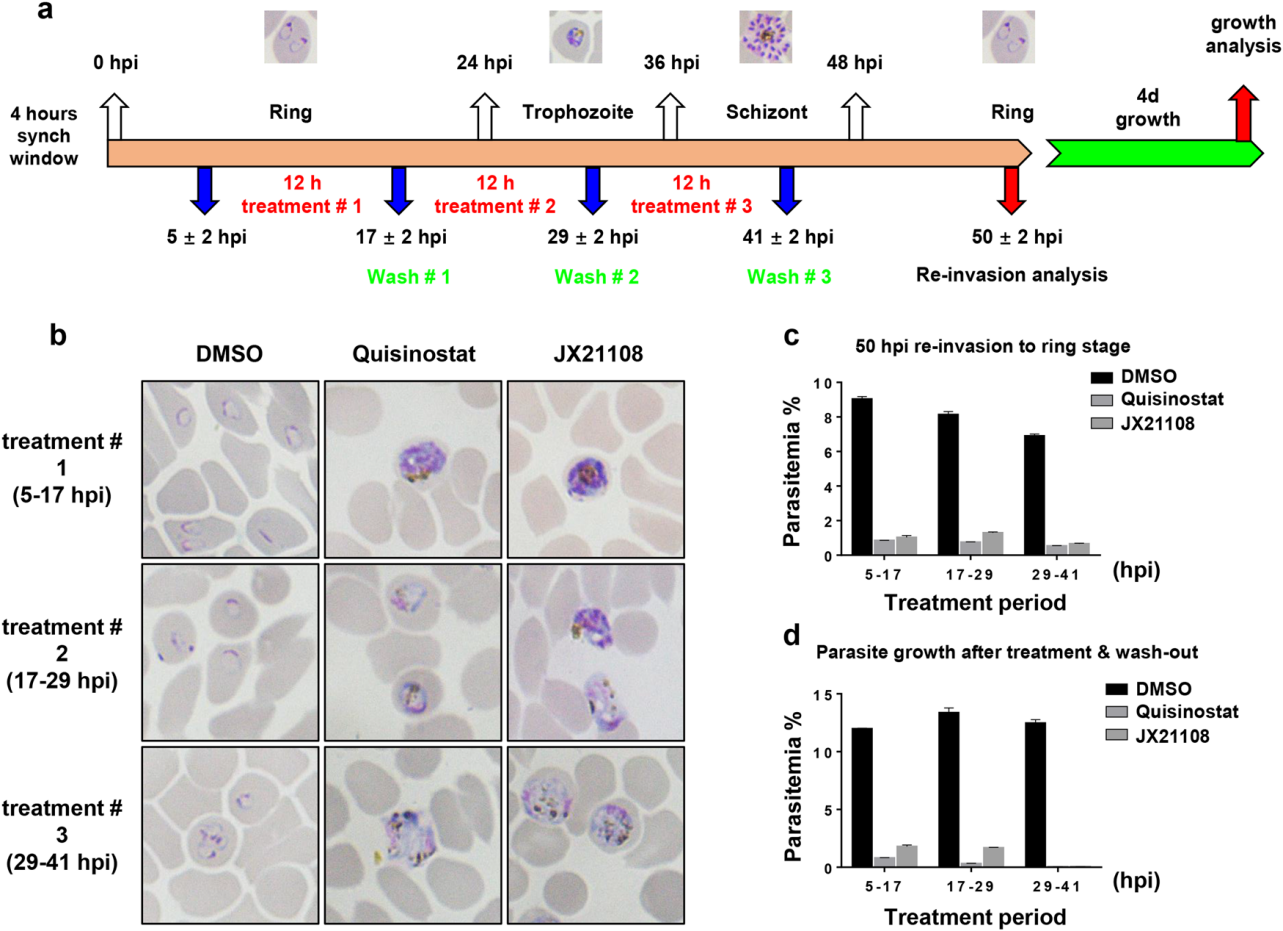
Stage-dependent antiplasmodial activity of JX21108 in the blood stage. **a** Schematics of the experimental design. Highly synchronized *P. falciparum* 3D7 parasites were treated for 12 h with 40 nM of the test compounds or DMSO as a control for three consecutive periods of the intraerythrocytic life cycle. **b** The morphology of parasites was evaluated by Giemsa-staining smear. **c** Parasitemia of reinvasion at 50 hpi was measured by flow cytometry. **d** Parasites were washed, followed by 1:40 dilution, and cultured for 4 days while parasitemia was measured by flow cytometry. Data in **c** and **d** are means ± SEM of 100,000 RBCs from three biological replicates.

We further evaluated the activity of JX21108 against different blood stages using highly synchronized *P. falciparum* parasites. Parasites were exposed to JX21108 at a 10× EC_50_ concentration (40 nM) for a 12-h treatment at the ring (5–7 hpi (hours post invasion)), trophozoite (17–29 hpi), and schizont (29–41 hpi) stages (Fig. 3a). Morphological analyses showed that compared to control treatment, JX21108 treatment at all blood stages could strongly block the asexual development of parasites to the next cycle (Fig. 3b), consistent with the parasitemia in the culture at 50 hpi, as monitored by flow cytometry (Fig. 3c). Further measurement of parasitemia at 4 dpi indicated that the treatment at the schizont stage was more active in parasite killing than that at the ring and trophozoite stages (Fig. 3d). Consistently, JX21108 and quisinostat showed similar stage-dependent activities. This result suggested that the parasiticidal mechanism of JX21108 may be associated with schizont growth or invasion of red blood cells (RBCs).

**Fig. 4:**
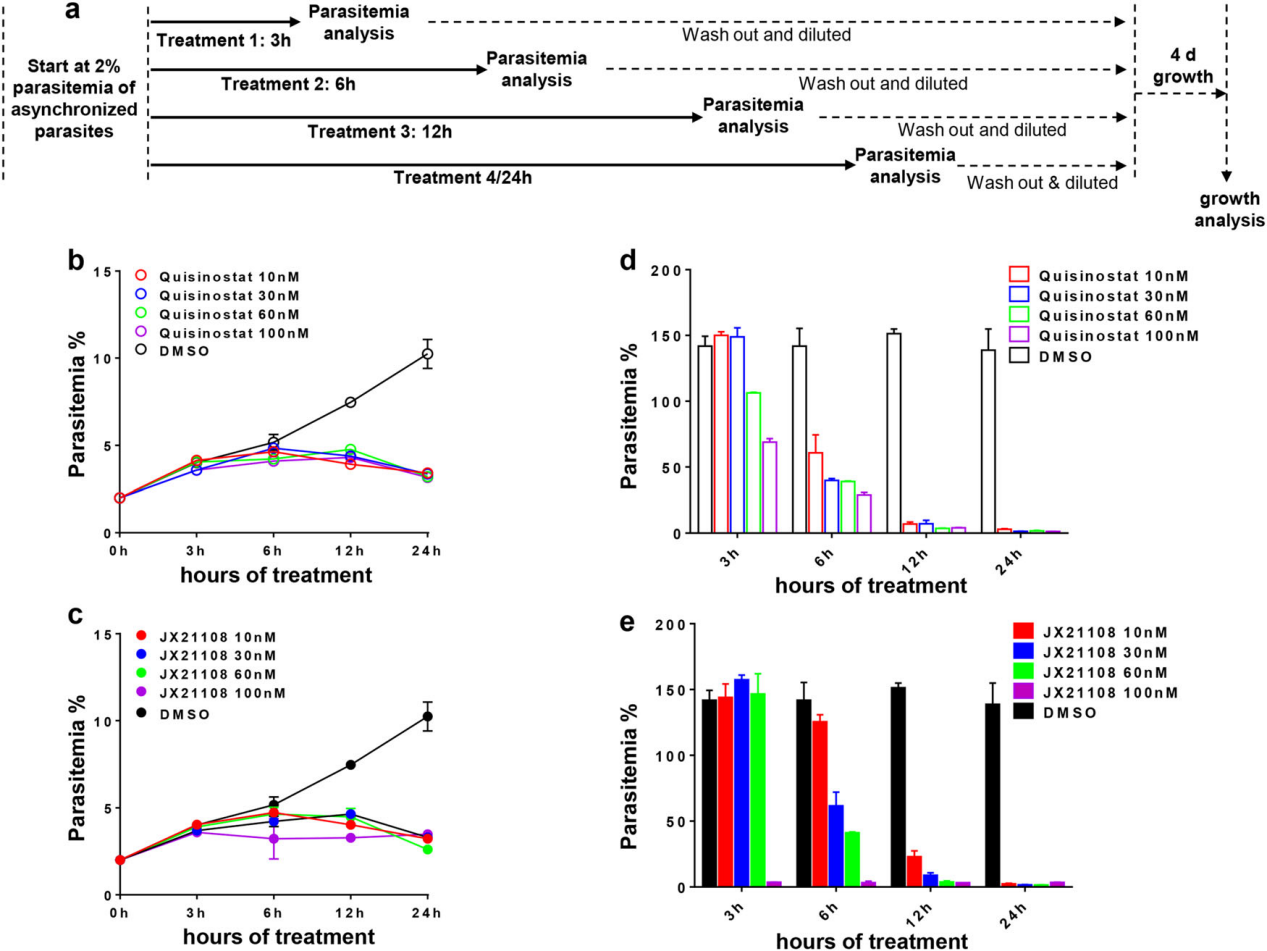
Time- and dose-dependent antiplasmodial activity of JX21108. **a** Schematics of the experimental design. Asynchronous 3D7 parasites were exposed to the tested compounds at the given concentrations for different time periods, as shown. **b**, **c** Parasites were exposed to the indicated concentrations of quisinostat (**b**) and JX21108 (**c**) for 3, 6, 12 or 24 h. Parasitemia was detected immediately after treatment by flow cytometry. **d**, **e** The treated parasites from **b** and **c** were washed, followed by 1:40 dilution and cultured for 4 days. Parasitemia was measured by flow cytometry. Data are means ± SEM of 100,000 RBCs from three biological replicates.

Moreover, dose- and time-dependent antiplasmodial activities of JX21108 against the blood stage were evaluated by using asynchronous 3D7 (Fig. 4a). Flow-cytometry analyses showed that 12- and 24-h treatments significantly inhibited the asexual growth of 3D7 parasites with no observed differences among the tested dosages of quisinostat (Fig. 4b) or JX21108 (Fig. 4c). By monitoring the long-term effect of a single, short period of treatment, we found that 6-h treatment with both quisinostat and JX21108 resulted in a substantial reduction in parasitemia when measured 4 days later (Fig. 4d, e). Both quisinostat and JX21108 showed dose-dependent activity against the blood stages (Fig. 4b–e). Notably, at a concentration of 100 nM JX21108, even 3 h of treatment completely blocked parasite growth (Fig. 4e).

### JX21108 targets PfHDAC1

**Fig. 5:**
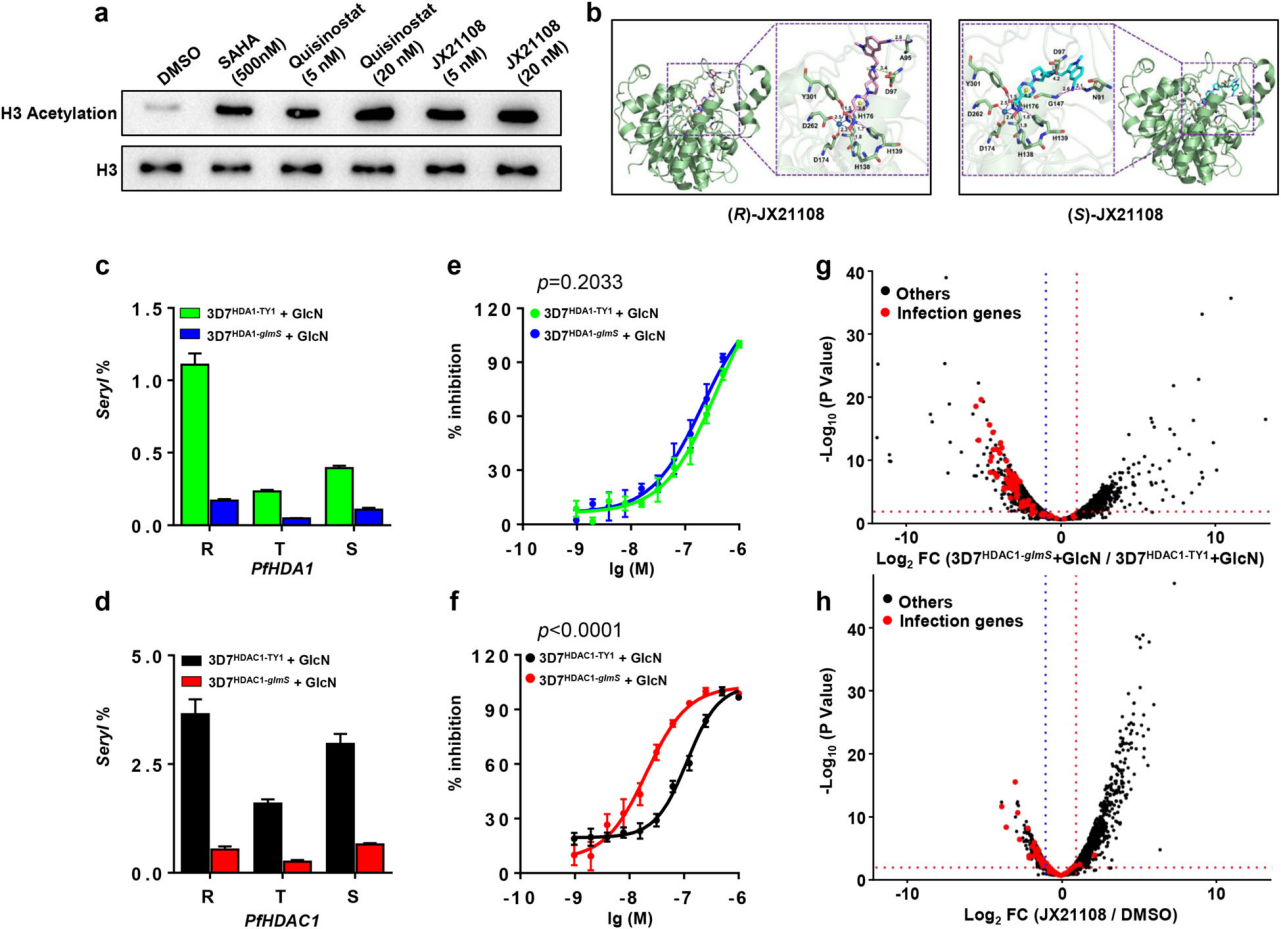
JX21108 targets PfHDAC1. **a** Asynchronous 3D7 parasites were treated with quisinostat, JX21108 or SAHA (a broad HDAC inhibitor) for 4 h. Histone H3 acetylation (Ace) levels were detected by western blot. Dimethyl sulfoxide (DMSO) was used as a negative control, and SAHA was used as a positive control. H3, histone H3, was used as a loading control. **b** In silico docking of a homology model of PfHDAC1 showing that (*R*)-JX21108 (left panel) and (*S*)-JX21108 (right panel) could be perfectly accommodated by the catalytic site surface of PfHDAC1. PfHDAC1 is shown as a green cartoon. Small molecules and key residues in PfHDAC1 are drawn as sticks. Carbons are in pink, cyan and orange in small molecules, and green in key residues. All oxygen, nitrogen, and hydrogen atoms (polar hydrogens only) are in red, blue, and white, respectively. Zn^2+^ and π-bond centers are represented as blue and yellow spheres, respectively. Metal bonds, hydrogen bonds, π bonds, and ionic bonds are shown as blue, purple, yellow, and green dashed lines, respectively. The distances of these interactions are also labeled near the dashed lines and shown in angstroms. **c**, **d** RT-qPCR detecting the gene expression of *PfHDA1* (**c**) and *PfHDAC1* (**d**) in the transgenic parasites 3D7^HDA1-TY1^, 3D7^HDA1-*glmS*^ (**c**) and 3D7^HDAC1-TY1^, 3D7^HDAC1-*glmS*^ (**d**) at the ring (R), trophozoite (T), and schizont (S) stages, respectively, after treatment with 2.5 mM GlcN. The expression values were normalized to those of a housekeeping gene *seryl-tRNA synthetase*. **e**, **f** EC_50_ of JX21108 against *P. falciparum* from **c** and **d**, respectively. *P* values were determined by two-tailed Student’s *t*-test. **g**, **h** Comparative transcriptome analysis. The log_2_ (TPM + 1) of 3D7^HDAC1-*glmS*^ with GlcN compared to that of 3D7^HDAC1-TY1^ with GlcN (**g**) and log_2_ (TPM + 1) of 3D7 treated with JX21108 compared to that of 3D7 treated with DMSO are shown for all parasite genes (**h**). Each infection gene is shown as a red dot. TPM, transcripts per million. Data in **c**–**f** are means ± SEM from three biological repeats with duplication. Data in **g** and **h** are from two RNA-seq experiments. The infection genes are listed in Supplementary Table S5.

To understand the parasiticidal mechanism of JX21108, the global histone H3 acetylation level after JX21108 treatment was first examined by western blot. The results showed that similar to the HDAC inhibitor SAHA, both quisinostat and JX21108 greatly increased the pan-acetylation levels of parasite histone H3 (Fig. 5a), showing possible HDAC inhibitory activity of JX21108 in *P. falciparum*. There are five *HDAC* genes in *P. falciparum*^47^. Phylogenetic analysis showed that *P. falciparum* HDAC1 (PfHDAC1, PF3D7_0925700), an essential gene for the blood stage^19^, is the only class I HDAC representative in *P. falciparum*, while PfHDA1 (PF3D7_1472200) and PfHDA2 (PF3D7_1008000) belong to class II HDACs, and PfSir2a (PF3D7_1328800) and PfSir2b (PF3D7_1451400) are phylogenetically close to class III HDACs^47^. Class I and II HDACs are the main targets of quisinostat^40^.

Sequence alignment showed that human HDAC2^48^ and HDAC8^49^ are the closest structural homologs of PfHDAC1 (Supplementary Fig. S3a, b). The 3D structure of PfHDAC1 was first modeled following the crystal structure of human HDAC8, which was co-crystallized with the trichostatin A (TSA), an HDAC inhibitor with the same active functional group in JX21108, hydroxamate. As the homology of human HDAC2 with the sequence from the N-terminus to the end of the L1 loop of PfHDAC1 is much closer than that of human HDAC8 (Supplementary Fig. S4b), this region of PfHDAC1 was remodeled following the human HDAC2 structure (Supplementary Fig. S3c). A Ramachandran plot illustrated that only 0.3% of residues are located in the disallowed region, verifying the rationale of our homology structure model to a certain extent (Supplementary Fig. S3d). The binding site of PfHDAC1 comprises a deep cavity that can accommodate active molecules. Zn^2+^ forms metal coordinations with Asp174, Asp262, and His176 in the predicted catalytic site of PfHDAC1, which makes up the lower surface of the active site^50^.

A molecular docking study^51^ was implemented to investigate the potential interaction between PfHDAC1 and JX21108. The binding energies of enantiopure (*R*)-JX21108 and (*S*)-JX21108 were −89.539 kcal/mol and −73.831 kcal/mol, respectively, compared to −83.127 kcal/mol for quisinostat (Fig. 5b and Supplementary Fig. S3e). The interactions occurring in the linker and tail group may stabilize the binding of JX21108 to PfHDAC1. Collectively, favorable intermolecular interactions and strong binding energies between PfHDAC1 and JX21108 may ensure the promising antiplasmodial activities of the drug.

To further determine whether PfHDAC1 is a molecular target of JX21108, the *PfHDAC1* gene was knocked down in 3D7 by using the CRISPR/Cas9 system to generate 3D7^HDAC1-glmS^^52,53^, which introduced the *glmS* sequence at the 3′-terminus of *PfHDAC1* for posttranscriptional degradation of the *PfHDAC1* mRNA (Supplementary Fig. S4a). As a control, a TY1 epitope was used to replace the *glmS* sequence to generate 3D7^HDAC1-TY1^ with no induced degradation of the *PfHDAC1* transcript. As PfHDA1 is related to class II HDACs, which are not the strongest targets of quisinostat, PfHDA1 was also knocked down in parallel as a control. Genetic manipulation was determined by PCR analyses (Supplementary Fig. S4b–e), and reduced mRNA levels for both *PfHDA1* and *PfHDAC1* were confirmed by RT-qPCR (Fig. 5c, d). Western blot analyses showed that the global histone acetylation levels were greatly increased in the presence of glucosamine (GlcN) in both 3D7^HDAC1-*glmS*^ and 3D7^HDA1-*glmS*^ (Supplementary Fig. S4f), consistent with the function of both PfHDAC1 and PfHDA1 as histone deacetylases of *P. falciparum*^36^. However, in control TY1-tagged parasites induced with GlcN, there was no increase in histone acetylation. Importantly, GlcN-induced *PfHDA1* KD in 3D7^HDA1-*glmS*^ did not change the sensitivity of the parasite to JX21108; the EC_50_ values of JX21108 are 330 ± 57 nM for 3D7^HDA1-TY1^ and 210 ± 25 nM for 3D7^HDA1-*glmS*^. *PfHDAC1* KD in 3D7^HDAC1-*glmS*^ resulted in significantly increased sensitivity (5.5-fold shift) of the parasites to JX21108 treatment, with an EC_50_ of 20.5 ± 6.95 nM compared to 114 ± 26.5 nM in 3D7^HDAC1-TY1^, suggesting that PfHDAC1 is a molecular target of JX21108 (Fig. 5e, f).

To further evaluate the molecular mechanism of PfHDAC1 function in *P. falciparum*, the schizont-stage transcriptomes of 3D7^HDAC1-*glmS*^ and 3D7^HDAC1-TY1^ in the presence of GlcN were compared by mRNA sequencing (RNA-seq) analysis (Fig. 5g; Supplementary Table S7). A total of 771 genes were downregulated, while 659 genes were upregulated after PfHDAC1 KD. Gene Ontology (GO) analyses of the RNA-seq data showed that most of the downregulated genes belong to infection-related gene terms, including rhoptry (GO:CC), entry into host cells (GO:BP), inner membrane pellicle complex (GO:CC), subcellular localization of proteins involved in invasion (MPMP), genes encoding GPI-anchored membrane proteins (MPMP), and components of the linear motor responsible for merozoite motility in invasion (MPMP). In addition, metabolic (GO:BP), host cell part (GO:CC), and mitochondrion (GO:BP) were the most enriched terms for upregulated genes (Supplementary Fig. S5). After JX21108 treatment of 3D7 strain at the schizont stage, 505 genes were downregulated and 837 were upregulated. Similar GO results were observed for downregulated genes with one exception: the metabolic process (GO:BP) term was enriched in downregulated genes by JX21108 treatment, while it was enriched in upregulated genes by PfHDAC1 KD (Fig. 5g, h; Supplementary Table S8, Fig. S5). Interestingly, 167 common genes were downregulated and 235 common genes were upregulated after PfHDAC1 KD or JX21108 treatment, and the downregulated genes were enriched to the similar GO terms mentioned above (Supplementary Fig. S5). Taken together, our results demonstrated that inhibition by JX21108 causes broadly similar alterations of gene expression to PfHDAC1 KD, especially for the downregulated genes. This finding supports PfHDAC1 as a target of JX21108.

## Discussion

Multidrug resistance in malaria parasites is a major threat against malaria elimination^2,3^. Despite many attempts that have been made to identify compounds with antimalarial activity, drug development still lacks target diversity, and the main malaria drugs are only efficacious to the asexual stage parasite. Thus, new antimalarial drugs with a new mechanism of action, no cross-resistance to current antimalarial drug, and multistage antimalarial activity are urgently needed for malaria control.

HDACs are potential targets for treatments of various human diseases^54–56^, especially for the development of antimalarial drugs^28,30,57^ due to its greater limitations and potentially less redundancy in the *Plasmodium* species. There are five putative HDACs in *P. falciparum*; PfHDAC1 (PF3D7_0925700) is the only class I HDAC representative in *P. falciparum*, while PfHDA1 (PF3D7_1472200) and PfHDA2 (PF3D7_1008000) belong to class II HDACs, and PfSir2a (PF3D7_1328800) and PfSir2b (PF3D7_1451400) are phylogenetically close to class III HDACs^47^. Several HDAC inhibitors with in vitro and in vivo antiplasmodial activity have been reported previously^21–35,37,38,57–61^. For instance, WR301801 was discovered via chemical modification based on the structure of SAHA and can inhibit the growth of plasmodial at a low-nanomolar range in vitro, and the selectivity index is about 1000. However, it failed to completely cure the *P. berghei*-infected mice^23^. In this investigation, we screened an epigenetic inhibitor library to find compounds that could irreversibly inhibit parasite growth in vivo and have good drug-like properties.

As a HDAC inhibitor, quisinostat mainly targets the human class I and class II HDACs. We demonstrated its high antiplasmodial activity both in vitro and in vivo (Supplementary Fig. S1). Due to the potential cytotoxicity, the chemical modification was conducted to optimize the toxicity by modifying the central diamine first, followed by optimization of the CAP group. Finally, a structurally novel PfHDAC inhibitor JX21108 with similar antiplasmodial activity and attenuated cytotoxicity compared to quisinostat was discovered (Fig. 1). We showed that JX21108 could be efficiently against a variety of geographically representative drug-sensitive and multidrug-resistant strains of *P. falciparum* with a low-nanomolar EC_50_ value of ~2–7 nM (Figs. 1, 2a, b), while the selectivity index was increased to 331/1060 compared to 9/8 of quisinostat (Fig. 1). The metabolic stability of JX21108 was improved compared to quisinostat by mouse liver microsome testing, and the in vivo PK value was also better than that of quisinostat, except the half-life. This may be because quisinostat had a significantly higher apparent volume of distribution (V/F) than JX21108, which might cause its wide distribution among tissues, while JX21108 might be mainly distributed and metabolized in blood. Thus, the slow release of quisinostat in tissues might contribute to the longer half-life in plasma, despite the higher clearance (CL) of quisinostat in both liver microsome and in vivo pharmacokinetic assays (Tables 1, 2; Supplementary Fig. S6).

It is worth noting that JX21108 can completely clean up the *P. yoelii* infection in mice with no relapse of malaria at a dose of 90 mg/kg for a 5-day treatment (Fig. 2c, d). In addition, JX21108 can also inhibit the growth of stage II and stage IV gametocytes efficiently (Fig. 2f, g). Our results suggested that JX21108 has a potential malaria transmission-blocking activity. All the results demonstrated that modifying the central diamine led to attenuated cytotoxicity and that appropriate optimization of the CAP group could further weaken cytotoxicity while retaining antiplasmodial activity compared to quisinostat. More importantly, JX21108 is active against not only the blood stage but also the gametocyte and liver stages. This activity provides the potential of the PfHDAC1 inhibitor function in blocking transmission and preventing malaria infection.

HDACs regulate various important cellular processes, and we demonstrated that PfHDAC1 is a target of JX21108 and the inhibition of PfHDAC1 by JX21108 would lead to the aberrant expression of genes, especially the downregulation of RBC infection-related genes, which results in the growth inhibition of parasites directly (Fig. 5 and Supplementary Fig. S5).

Although JX21108 was discovered with the same antiplasmodial activity and reduced cytotoxicity, the in vitro enzyme activity assay showing that JX21108 still has a strong inhibition activity to human HDAC1 indicates potent cytotoxicity of JX21108 (Supplementary Table S3). Thus, further structural optimization of JX21108 would be conducted to optimize safety. Moreover, given the relatively short half-life of JX21108 in mouse blood (Table 2), a combination of JX21108 with another long-half-life antimalarial drug would be worth investigating.

## Materials and methods

### Epigenetic inhibitor library

The compound library, comprising 41 characterized inhibitors targeting HDACs, 12 targeting histone demethylases (HDMs), 2 targeting histone methyltransferases (HMTs), and 9 targeting bromodomain and extraterminal domain (BET) proteins, was customized and synthesized by WuXi AppTec.

### Parasite culture and transfection

*P. falciparum* was cultured according to a standard protocol^62^. Briefly, all parasites were cultured in completed medium (CM) consisting of hypoxanthine (50 mg/L), RPMI (10.44 g/L) supplemented with HEPES (5.94 g/L), Albumax (5 g/L), sodium bicarbonate (2.2 g/L), and gentamycin (50 mg/L) in an atmosphere consisting of 5% CO_2_, 5% O_2_, and 90% N_2_. Parasite transfections were performed by electroporation at the ring stage of 7% parasitemia by using 50 μg of the plasmid, as reported previously^52^. Transfected parasites were selected by 2.5 μg/mL Blasticidin S Deaminase (Life Technologies) or 5 nM WR99210 (Jacobus Pharmaceuticals), and cloned by limiting dilution. Parasite genomic DNAs were extracted for validation of gene editing by PCR.

### Plasmid constructs

All molecular cloning steps were carried out by using a ClonExpress II One Step Cloning Kit (Vazyme C112–02). The fusion of 3× TY1 or 3× TY1 plus *glmS* at the C-terminus of PfHDA1 or PfHDAC1 was conducted by using the CRISPR/Cas9 system. The homologous arms of *PfHDA1* and *PfHDAC1* were amplified by using primers 1/2 and 3/4, primers 5/6 and 7/8, respectively. The 3× TY1 tag and *glmS* sequences for *PfHDA1* editing were amplified by using primers 9/10 and 11/12, respectively, and for *PfHDAC1* by using primers 13/14 and 15/16. The full donor sequences were amplified by overlap PCR using primers 1/4 for *PfHDA1* and primers 5/8 for *PfHDAC1*. The purified PCR products were inserted into pL6CS at the ASC I/Afl II sites. The sgRNA for knock-in of *PfHDA1* and *PfHDAC1* was made by annealing primers 21/22 with the antisense strand. Gene knock-in was confirmed by PCR using primers 17/18 and 19/20, followed by DNA sequencing. All primer sequences used in this study are shown in Supplementary Table S4.

### Antiplasmodial activity screening of the epigenetic inhibitor library

Each of 64 epigenetic inhibitors was dissolved in DMSO to generate a 10 mM stock. The stocks were diluted with CM to 100 nM or 1 μM, and highly synchronized ring-stage parasite culture by 5% sorbitol (1% parasitemia, 2% hematocrit) were incubated in a 96-well plate with compounds at a concentration of 50 nM or 500 nM in a total volume of 200 μL. The parasites were cultured at 37 °C in an atmosphere consisting of 5% CO_2_, 5% O_2_, and 90% N_2_ for 72 h. Parasites were lysed by adding 100 μL of lysis buffer (0.12 mg/mL saponin, 0.12% Triton X-100, 30 mM Tris-HCl, and 7.5 mM EDTA) containing 5× SYBR Green I (Invitrogen; supplied as 10,000× dilution) to each well, followed by a 2-h incubation in the dark. The fluorescence signal representing parasite DNA was monitored on an instrument at 485-nm excitation and 535-nm emission. DMSO was used as a negative control. Growth inhibition was normalized to that of the DMSO control. The activity screening was performed by three independent assays with two technical replicates each time.

### In vitro growth inhibition assay for EC_50_ determination

A 3-day SYBR Green I inhibition EC_50_ assay was performed as described previously^63^. Briefly, highly synchronized ring-stage parasites by 5% sorbitol (1% parasitemia and 2% hematocrit) were incubated in a 96-well plate with compounds in a total volume of 200 μL. The compounds were added from an initial concentration of 200 nM with a 2-fold gradient dilution for a total of 11 test points. The parasites were cultured for 72 h before SYBR Green I staining as described above. The EC_50_ was calculated by GraphPad Prism. DHA was used as a positive control, and the final concentration of DMSO was 1%. Three independent assays with two technical replicates each time were conducted in this experiment.

### In vitro cytotoxicity analysis

The mammalian cell lines HepG2 and 293T were cultured in 10-cm dishes at 37 °C and 5% CO_2_ with Dulbecco’s Modified Eagle Medium consisting of 10% fetal bovine serum and 1% penicillin/streptomycin. When starting the cytotoxicity analysis, 10,000 cells per well were seeded in a white 96-well plate in a total volume of 100 μL and cultured for 24 h. After 24 h, the medium was replaced with a new medium containing the tested compounds, added at concentrations ranging from the highest concentration of 100 μM with a 2-fold gradient dilution for a total of 11 test points. The cells were therefore cultured for 72 h before the analysis of the cell viability assay by using CellTiter-Glo (Promega, Cat# G7572). In all, 1% DMSO was used as a negative control. The inhibition of each drug was normalized to that of the DMSO control. EC_50_ was calculated by GraphPad Prism. Three independent assays with two technical replicates each time were conducted in this experiment.

### In vivo blood-stage inhibition assay

Experiment 1: Five female BALB/c mice (6 weeks, 18 g) from each experimental group were infected with 1 × 10^5^ rodent malaria parasite *P. yoelii* via intraperitoneal (i.p) injection^64^. After 24 h, the mice were treated with 30, 60, or 90 mg/kg/day JX21108 via i.p injection and 20 mg/kg/day piperaquine phosphate (PPQ) as a positive control for 5 days. DMSO (5%) was used as a blank control.

Experiment 2: To analyze the differences in drug potency between racemic and enantiopure forms of JX21108, five female BALB/c mice (6 weeks, 18 g) per group were treated with 60 mg/kg/day (+)-JX21108, (−)-JX21108, or JX21108 as described above. Blood smears were made daily from the blood of veins and stained by Giemsa. Parasitemia was counted from at least 5000 RBCs by microscopy. The bodyweight of the mice and the number of deaths were recorded daily. All the graphs were generated by GraphPad Prism. All experimental procedures followed the National Institutes of Health Guide for the Care and Use of Laboratory Animals, as well as the guidelines of the Animal Welfare and Committee of Institut Pasteur of Shanghai, Chinese Academy of Science, IACUC issue No. A2018009.

### RSA

An RSA was performed as described previously^65^ to evaluate the activity of JX21108 against the artemisinin- and piperaquine-resistant *P. falciparum* clinical isolates 6218 and 6320^43^. Briefly, parasites were highly synchronized at 0–3 hpi by a 60%/40%-Percoll gradient^66^ followed by 5% D-sorbitol^67^ treatment after 3 h. The parasite suspension (0.5%–1% parasitemia, 2% hematocrit) was incubated with compounds at 700 nM in a total volume of 2 mL in a 48-well plate for 6 h. After that, the culture mixture was washed three times with 15 mL of CM before another 66 h of cultivation with CM. A Giemsa-stained blood smear was made after cultivation. Parasitemia was counted from at least 10,000 RBCs by microscopy. The survival rate was calculated as the percentage of that in DMSO control. DHA at a concentration of 700 nM served as a positive control. Three independent assays with two technical replicates each time were conducted in this experiment.

### In vivo liver-stage parasite inhibition assay

An in vivo liver-stage parasite inhibition assay was conducted as described previously^68,69^. Briefly, five female BALB/c mice (6 weeks, 18 g) per group were infected with 5000 *P. berghei-ANKA-GFP-luc* sporozoites by i.v injection followed immediately by i.v.-administered JX21108 at a single dose of 30 mg/kg or 60 mg/kg (day 0). The β-cyclodextrin was used as a blank control. For fluorescence detection, 3 mg of D-luciferin sodium (Sinochrome BC-220–10) in 100 μL of ddH_2_O was i.p. injected into each mouse. The fluorescence signals were visualized by an IVIS Lumina series (PerkinElmer) on days 1–4, 6, and 8 after infection. All experimental procedures followed the National Institutes of Health Guide for the Care and Use of Laboratory Animals, as well as the guidelines of the Animal Welfare and Committee of Institut Pasteur of Shanghai, Chinese Academy of Science, IACUC issue No. A2018009.

### In vitro gametocyte inhibition assay

To test the activities of JX21108 against different gametocyte stages, the *P. falciparum* producer NF54^p230p-*luc*^ was utilized, as described previously^18^. Stage II (day 3 after induction) and stage IV (day 9 after induction) gametocytes were isolated by a 70%/40%-Percoll gradient and seeded in 96-well plates, followed by exposure to JX21108 at an initial concentration of 10 μM with a 2-fold gradient dilution for a total of 12 test points, for 48 h in a total volume of 100 μL. Luciferase intensity based on luciferase expression was detected as described above. The EC_50_ was calculated by GraphPad Prism. DMSO (1%) was used as a negative control. Three independent assays with two technical replicates each time were performed.

### Stage-specific parasite inhibition assay

To analyze the stage-specific parasite inhibition, highly synchronized parasites were cultured in 24-well plates with a starting parasitemia of 1% at a hematocrit of 2%. After 12 h of culture with the tested compounds at a concentration of 40 nM, parasites were washed three times to remove the compounds, and parasitemia was diluted 1:40 by new RBCs into CM for another 4 days of culture. DMSO was used as a blank control. Parasitemia was counted by flow cytometry. Flow cytometry was conducted as described previously^70^. Briefly, the parasite-infected RBCs were fixed in 4% formaldehyde/0.015% glutaraldehyde for 30 min at room temperature and incubated with 500 μL of a SYBR green staining solution (Invitrogen) (1:5000) in 1× PBS (phosphate-buffered saline, pH 7.4, without calcium and magnesium). Cells were washed three times with 1× PBS prior to flow-cytometry analysis on a MoFloAstrios EQ instrument (BECKMAN COULTER). RBCs (5 × 10^5^) were measured (excitation 488 nm; emission detection FL1 513 ± 26 nm), and a value of 5 on FL1 (SYBR Green intensity) was applied as a threshold to identify (gate) the infected RBCs (iRBCs). Acquired data were processed using the FlowJo software (v.10.0.5). Three independent assays with two technical replicates each time were performed.

### Time- and dose-dependent parasite inhibition assay

Asynchronous parasites (2% parasitemia, 4% hematocrit) were treated with the tested compounds at a concentration of 10, 30, 60, or 100 nM, respectively. At 3, 6, 12, and 24 h post treatment, parasitemia of each sample was detected by flow cytometry to investigate the time-dependent activity. Parasites from the same treatment were further washed to remove compounds, and parasitemia was diluted 1:40 by new RBCs into CM for an additional 4 days of culture. Parasitemia of each sample was detected by flow cytometry as described above to investigate the dose-dependent activity. DMSO was used as a blank control. Three independent assays with two technical replicates each time were performed.

### The sensitivity of 3D7^HDAC1-*glmS*^ and 3D7^HDA1-*glmS*^ to JX21108

Highly synchronized schizont-stage parasites were prepared and treated as described above, with adjustments. In brief, the parasites were exposed to JX21108 at an initial concentration of 1 μM with a 2-fold gradient dilution for a total of 11 test points, for 4 h. After washing, parasites were cultured in new CM for another 68 h. Parasite growth was monitored by SYBR Green I staining, as described above. The EC_50_ was calculated by GraphPad Prism. Three independent assays with two technical replicates each time were performed.

### Pharmacokinetic assay

Five female BALB/c mice (6–8 weeks, 18–19 g) per group were purchased from JH Laboratory Animal Co., Ltd. All mice were raised at controlled temperature and humidity and had free access to food and water. Compounds were dissolved in 5% DMSO and diluted with 20% aqueous 2-hydroxypropyl-β-cyclodextrin solution to a stock concentration of 0.5 mg/mL. Each mouse was i.p. injected with the compound solution at a dose of 5 mg/kg. Blood samples collected by anticoagulant (K_2_EDTA)-containing tubes at 0.25, 0.5, 1, 2, 4, 8, and 24 h after injection were centrifuged at 2000× *g* at 4 °C for 5 min to obtain plasma. LC-MS/MS analysis of samples was carried out by an ACQUITY UPLC HSS T3 1.8-μm column. The mobile phase was a mixture of phase A (0.1% formic acid in water) and phase B (0.1% formic acid in acetonitrile), which ran in gradient mode at a flow rate of 0.6 mL/min at 60 °C. Mass spectra were obtained on an API6500 triple quadrupole equipped with an ESI source. Propranolol was used as an internal standard. The plasma concentrations of the compounds were analyzed, and the pharmacokinetic parameters were calculated via WinNonlin.

### Liver microsome metabolism assay

Liver microsomes (0.5 mg/mL) were purchased from Corning. Ketanserin was selected as a positive control. The tested compounds were first dissolved in DMSO to a concentration of 10 mM and then diluted to 0.5 mM with acetonitrile. Liver microsome buffer was used to dilute the compounds to a working concentration of 1.5 µM, 30 µL of which was mixed with 15 µL of 6 mM NADPH at 37 °C. At 0, 5, 15, 30, and 45 min after incubation, 135 µL of acetonitrile was added to quench the reaction. The mixture was shaken on a vortex mixer (IKA, MTS 2/4) for 10 min at 600 rpm/min and then centrifuged at 5594× *g* for 15 min (Thermo Multifuge×3R). The supernatant was diluted 1:1 into distilled water, and analyzed by LC-MS/MS.

### Human HDAC inhibition assay

Human HDACs 1–3 (Cat# 50058, 5002, 50003), 6 (Cat# 50046), and 8 (Cat# 50008) and Sirt 2 (Cat# 50013) were purchased from BPS Bioscience. SAHA and suramin were used as positive controls. The tested compounds were dissolved into 20 mM stock in 100% DMSO. First, each of the tested compounds at concentrations of 10, 3.33, 1.11, 0.37, 0.12, 0.041, 0.014, 0.0046, 0.0015, and 0.005 µM were incubated with 15 μL of enzyme/Tris buffer solution at room temperature for 15 min. Ten microliters of trypsin and Ac-peptide substrate/Tris buffer solution were added to start the reaction, followed by a 1-h incubation at room temperature for HDACs 1–3 and 6. Ten microliters of Ac-peptide substrate/Tris buffer solution was added to start the reaction, followed by a 4-h incubation at room temperature and a further 2-h incubation with trypsin solution for HDAC8 and Sirt 2. Enzyme activities were measured on a Synergy MX with excitation at 355 nm and emission at 460 nm. This experiment was performed only once.

### Western blot

The asynchronous parasites were treated with quisinostat (5 nM, 20 nM) or JX21108 (5 nM, 20 nM) for 4 h, 1% DMSO was used as a negative control, and SAHA (500 nM) was used as a positive control. Then the parasite samples were harvested and treated with 0.15% saponin to remove the RBC proteins and sonicated in 1% NP40 lysis buffer. After centrifugation at 12,000× *g* for 15 min, the supernatant was resuspended in a routine SDS loading buffer. Parasite proteins were separated by 10% SDS-PAGE and transferred onto PVDF membranes (Millipore). Immunodetection was carried out using an anti-acetyl-histone H3 (Millipore; 06–599) or anti-histone H3 antibody (ABclonal; A2348) followed by a secondary antibody conjugated with HRP (Jackson Immuno Research Laboratories). The chemiluminescent HRP substrate Immobilon Western Kit (Millipore) was used for signal detection.

### RNA-seq and RT-qPCR

Parasite total RNA was extracted by using a TRIzol kit (Life Technologies) according to the manufacturer’s instructions. Four micrograms of the total RNA was used for mRNA-seq library construction by using a KAPA Stranded mRNA-Seq Kit (KAPA Biosystems, KK8240) according to the manufacturer’s instructions. Deep sequencing was performed on an HS X10-PE150 (Illumina) at GENEWIZ. The complementary DNAs were synthesized using a HiScript II Q Select RT SuperMix kit for qPCR (Vazyme, R223-01) according to the product manual. qPCR was performed on an ABI 7900 system with the following program: 5 min at 95 °C for initial denaturing, followed by 40 cycles of 10 s at 95 °C, 20 s at 50 °C, and 30 s at 60 °C. A housekeeping gene *seryl-tRNA synthetase* (PF3D7_0717700) was used as an internal control. qPCR primers for detecting mRNA expression of *PfHDA1* and *PfHDAC1* are shown in Supplementary Table S4. Two independent assays were performed for RNA-seq and three for RT-qPCR, and the raw RNA-seq data have been uploaded to the NCBI database, the accession number is PRJNA659150.

### RNA-seq analysis pipeline

The raw data of the next-generation sequencing were filtered as previously described and aligned to the *P. falciparum* v36 genome using HISAT2 (version 2.1.0) with default parameters^71^. Alignment files were converted to “BAM” format and merged using SAMtools. The duplicates were removed using Picard (version 2.18.6) with default parameters. Transcripts Per Million (TPM) were calculated using Stringtie (version 1.3.4, parameters “-e --rf -j 1”)^72^. Analysis of differential expression was conducted using DESeq2 (version 1.16.1)^73^ in the R platform, and a scatter plot was generated by the ggplot2 package (version 3.1.1)^74^ in R with the logarithm TPM values [log_2_ (TPM + 1)] as axes. GO analysis was performed in PlasmoDB. Perl module Text::NSP::Measures::2D::Fisher::two-tailed based on Fisher’s exact test was chosen to calculate the *P* values.

### Homology modeling

The protein sequence of PfHDAC1 (GenBank: CAD51938.1) was retrieved from the NCBI Protein Database. Homology modeling studies were performed on the Prime module of Maestro 10.5 embedded in the Schrödinger software package (2016-1). Within Prime, the fasta format of the PfHDAC1 protein sequence was used to run a BLAST search to find its structural homologs. The closest structural homologs were human HDAC2 (PDB ID: 3MAX) and human HDAC8 (PDB ID: 1T64) with sequence identities of 61% and 41%, respectively. The initial alignment was further modified manually to generate the final template sequence. 3MAX was utilized for the template of the N-terminus to Lys27 of PfHDAC1 while the rest of the residues were built based on the 3D structure of 1T64. The homology model of PfHDAC1 was constructed using an energy-based method. Zn^2+^ and its metal coordination were generated from the structure of 1T64 during the model generation process. Refine loops were utilized for refinement of non-template residues using the OPLS-2005 force field under the VSGB solvation model. Atoms within 8.5 Å of non-template residues were further minimized to optimize the local environment of loop regions using the OPLS-2005 force field under the VSGB solvation model. PDBsum (http://www.ebi.ac.uk/thornton-srv/databases/pdbsum/) was used to generate a Ramachandran plot of our modeled PfHDAC1.

### Molecular docking

Docking studies were performed on Maestro 10.5 embedded in the Schrödinger software package (2016–1). Protein Preparation Wizard was utilized for the PfHDAC1 homology model. Protein energy was minimized with a root-mean-square deviation value of 0.3 Å using the OPLS-2005 force field. LigPrep was employed to prepare small molecules utilizing the OPLS-2005 force field. The protonation states of small molecules were generated using Epik at pH values from 5.0 to 9.0, and metal-binding states were added during the preparation process. The inner box (the docking search space) of the binding site was centered on Zn^2+^-enclosing residues located within 15 Å around it; the grid box was set to 25 Å to ensure enough space to accommodate all ligand atoms. A metal constraint was set for Zn^2+^ due to its significant role in catalytic function. Then, the glide-stand precision (SP) model was applied to dock our small molecules to PfHDAC1 for the investigation of potential interaction mechanisms. The metal constraint was retained during the docking process to remove unreasonable ligand conformations. Prime MM-GBSA was applied to calculate binding energies between the most superior rational conformations of small molecules and PfHDAC1. 3D interaction figures were generated by PyMOL 1.5.

### Statistical analysis

Graphical representation and statistical analyses were performed using Prism 7 software. Unless otherwise stated, the results are shown as means ± SEM from three independent experiments in duplicate. Differences were tested for statistical significance using two-tailed Student’s *t*-test.

## Supplementary information

Supplementary information

Supplementary Table S7

Supplementary Table S8

## Supplementary information

**Supplementary Information** accompanies the paper at (10.1038/s41421-020-00215-4).
