# Supplementary material for: A novel multistage antiplasmodial inhibitor targeting Plasmodium falciparum histone deacetylase 1
Source: Cell Discov. 2020 Dec 11;6:93. doi: 10.1038/s41421-020-00215-4 (PMC7733455; doi:10.1038/s41421-020-00215-4)
Supplement: Supplementary file 1 — Supplementary information [file 41421_2020_215_MOESM1_ESM.pdf]

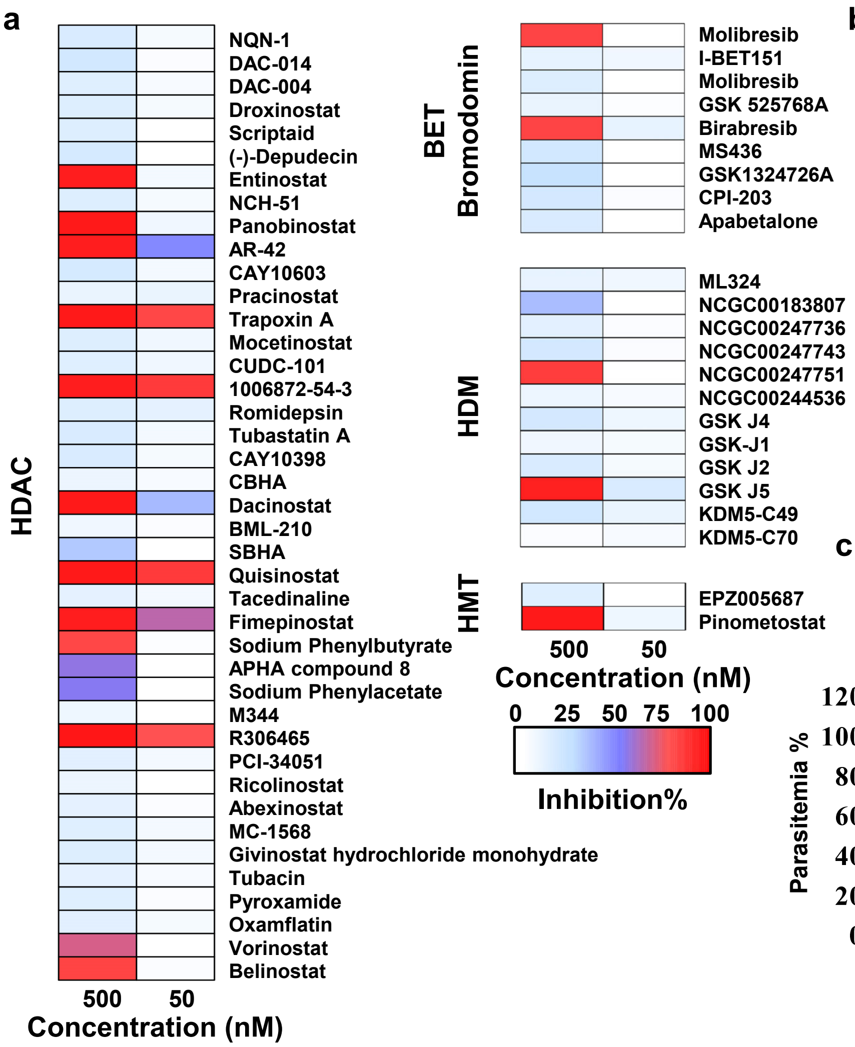

b

Drugs	EC50 (nM) *Pf*3D7	HepG2	293	SI[a]	SI[b]
Quisinostat	5.2 ±0.7	48 ±2	42 ±1.4	9	8
R306465	23 ±3.2	877 ±24	6601 ±125	38	287
1006872-54-3	32 ±5.2	129 ±11	398 ±25	4	12
Fimepinost at	63 ±9.5	70 ±13	69 ±8.6	1	1
Trapoxin A	82 ±10	6318 ±220	724 ±65	77	9

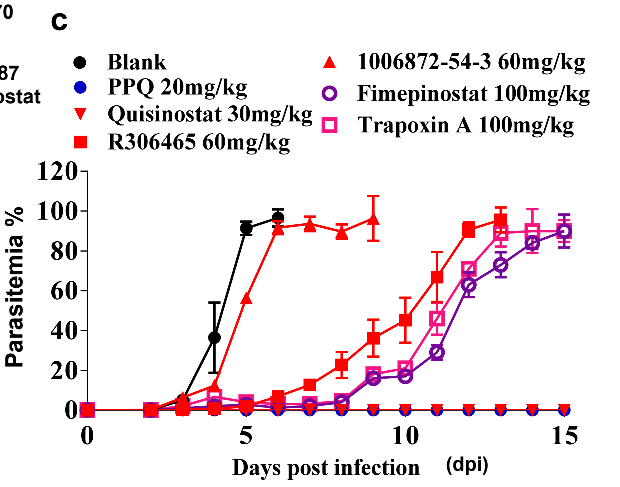

Supplementary Fig. S1 Screening of epigenetic inhibitors against blood-stage *P. falciparum*. a A heatmap indicates the percent inhibition of 3D7 asexual growth at 50 nM and 500 nM tested compounds compared to that with the DMSO control. **b** The EC_{50} values of five compounds against *Pf*3D7, HepG2 and 293T cells; SI[a], EC_{50} HepG2/ EC_{50} *Pf*3D7; SIb, EC_{50} 293T/EC_{50} *Pf*3D7. **c** *In vivo* activity assay against the blood stage of P. yoelii in BALB/c mice. Three mice were used in each group. Parasitemia was calculated from at least 5,000 red blood cells (RBCs) by microscopy. Blank, untreated group; PPQ, piperaquine phosphate. The administration route was intraperitoneal (i.p.). The data of **a** and **b** are from three independent biological replicates in duplication.

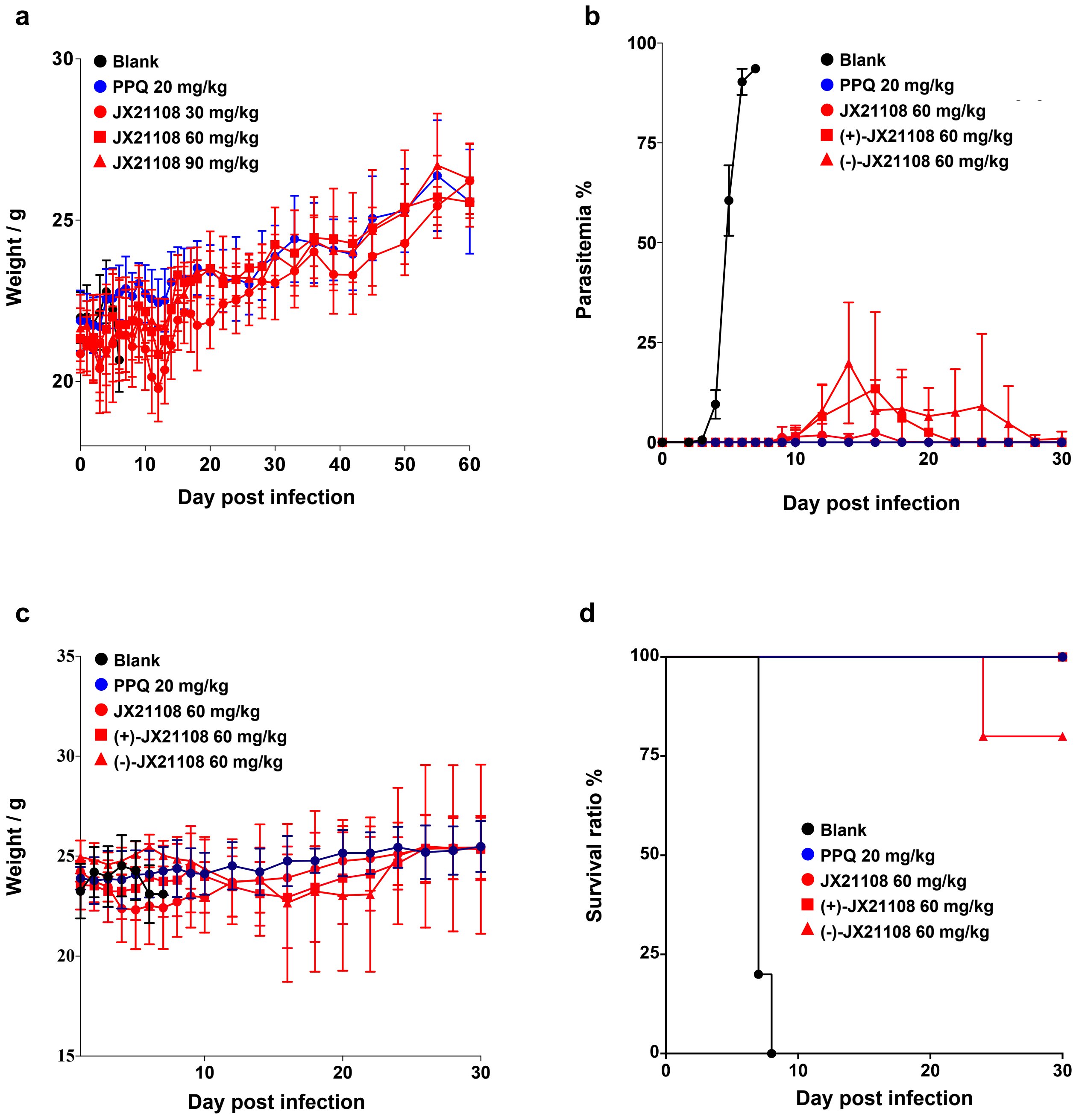

Supplementary Fig. S2 *In vivo* activity of JX21108 against *P. yoelii* in BALB/c mice. a The body weight of the tested mice from the experiment described in **Fig. 2c. b** *In vivo* activity of JX21108,(+)-JX21108 and (-)-JX21108 against the blood stage of *P. yoelii* in BALB/c mice. Five mice were used in each group. Parasitemia was calculated from at least 5,000 RBCs by microscopy. Blank, untreated group; PPQ, piperaquine phosphate. **c** and **d** The body weight and survival ratio of the tested mice from the experiment described in **c**. All data are the mean ± *s.e.m*.

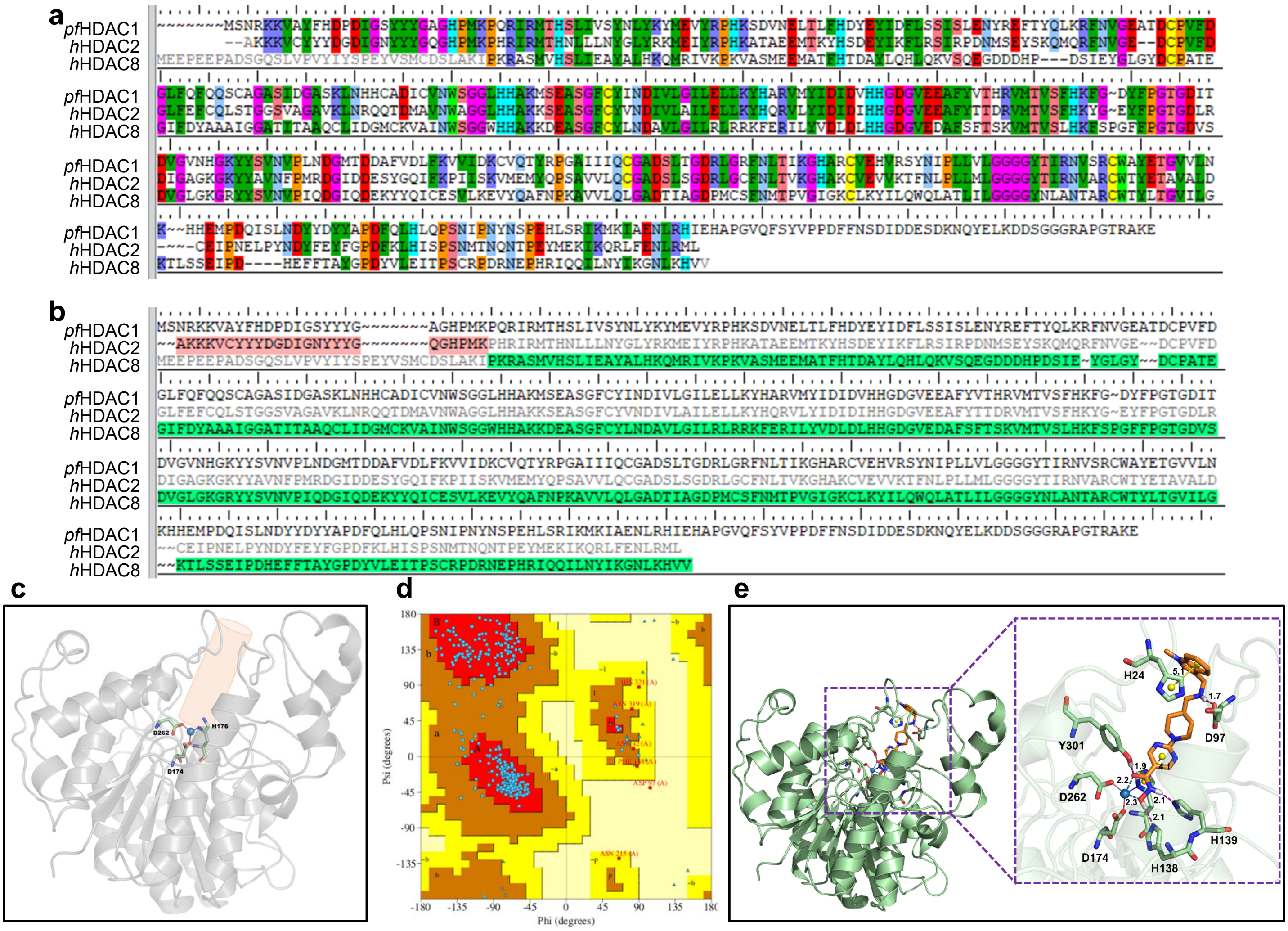

Supplementary Fig. S3 3D structure modeling of PfHDAC1. a Multiple sequence alignment of PfHDAC1, human HDAC2 and human HDAC8 was utilized for the generation of the PfHDAC1 model. **b** The template sequence used for model construction, in which reference sequence regions are labeled in pink for human HDAC2 and green for HDAC8. **c** Cartoon representation of the PfHDAC1 model. Zn^{2+} is shown as blue sphere and its coordinated residues are shown as sticks. The active site is illustrated by pale orange column. **d** Ramachandran plot of the PfHDAC1 model. **e** *In silico* docking of a homology model of PfHDAC1 showing that quisinostat could be perfectly accommodated by the catalytic site surface of PfHDAC1. PfHDAC1 is shown as green cartoon. Small molecules and key residues in PfHDAC1 are drawn as sticks. Carbons are shown in pink, cyan and orange, in small molecules and green in key residues. All oxygen, nitrogen and hydrogen atoms (polar hydrogens only) are colored red, blue and white, respectively. Zn^{2+} and π bond centers are represented by blue and yellow spheres, respectively. Metal bonds, hydrogen bonds, π bonds and ionic bonds are shown as blue, purple, yellow and green dashed lines, respectively. The distances of these interactions are also labeled near the dashed lines and shown in angstroms.

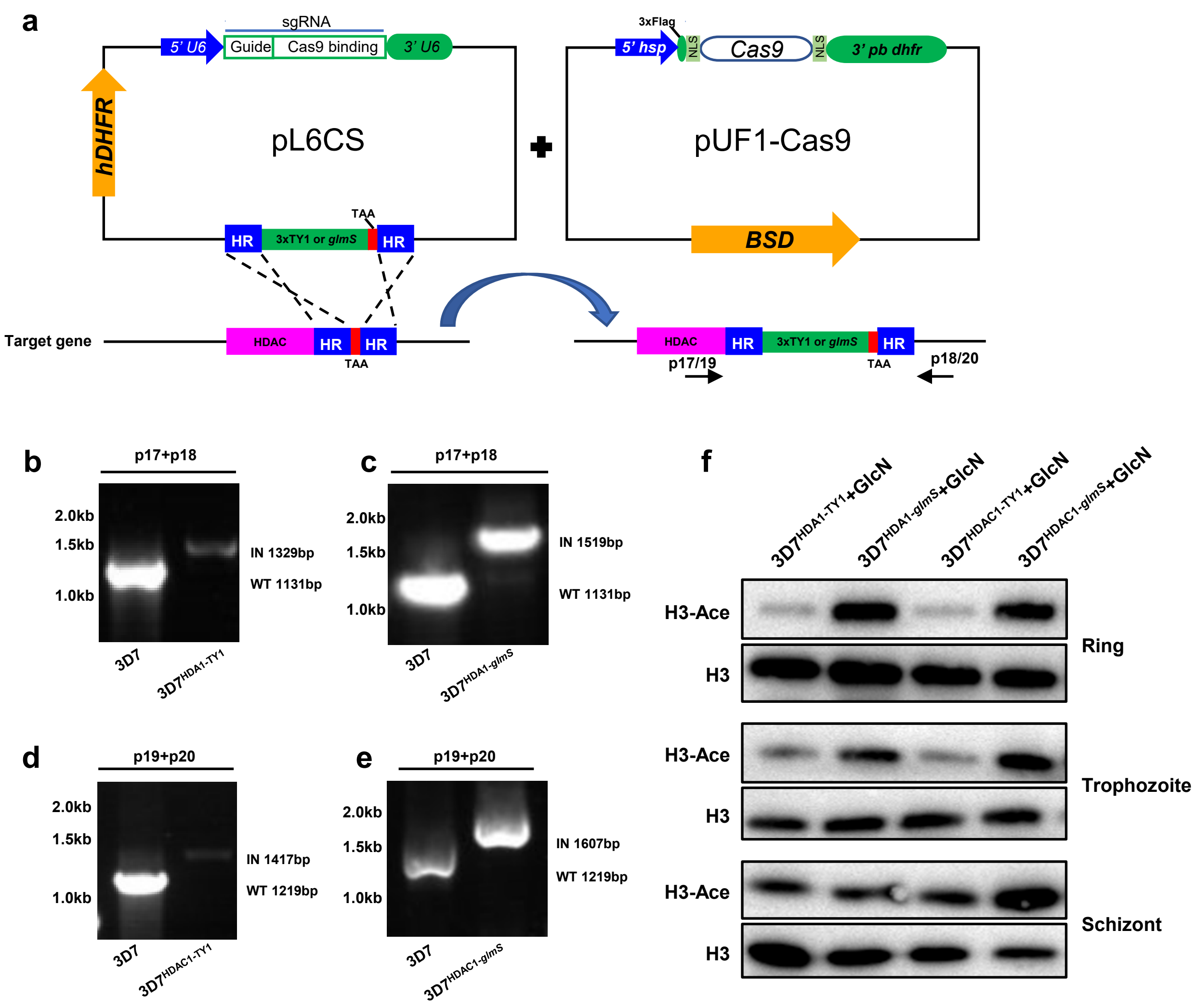

Supplementary Fig. S4 Knockdown of *PfHDA1* and *PfHDAC1*. a Schematic of the gene knockdown strategy using the CRISPR/Cas9 system. HR1, the 5' flanking fragment for homologous recombination. HR2, the 3' flanking fragment for homologous recombination. *hDHFR*, human dihydrofolate reductase. *BSD*, Blasticidin S Deaminase. 3 × TY1, a tag sequence for detection of the recombinant protein by western blot. *glmS*, sequence of the ribozyme for posttranscriptional degradation of the target gene induced by glucosamine (GlcN). p17/19 and p18/20, primers 17, 18, 19 and 20 used for PCR identification of gene editing (see also in Supplementary information, Table S6). **b-e** PCR identification of gene editing at the genomic loci of *PfHDA1* (**b** and **c**) and *PfHDAC1* (**d** and **e**). **f** Western blot analysis of the histone H3 acetylation levels (H3-Ace) of GlcN-treated 3D7$^{HDA1\text{-}TY1}$, 3D7$^{HDA1\text{-}glmS}$, 3D7$^{HDAC1\text{-}TY1}$, and 3D7$^{HDAC1\text{-}glmS}$ at the ring, trophozoite and schizont stages. Histone H3 (H3) was used as the loading control.

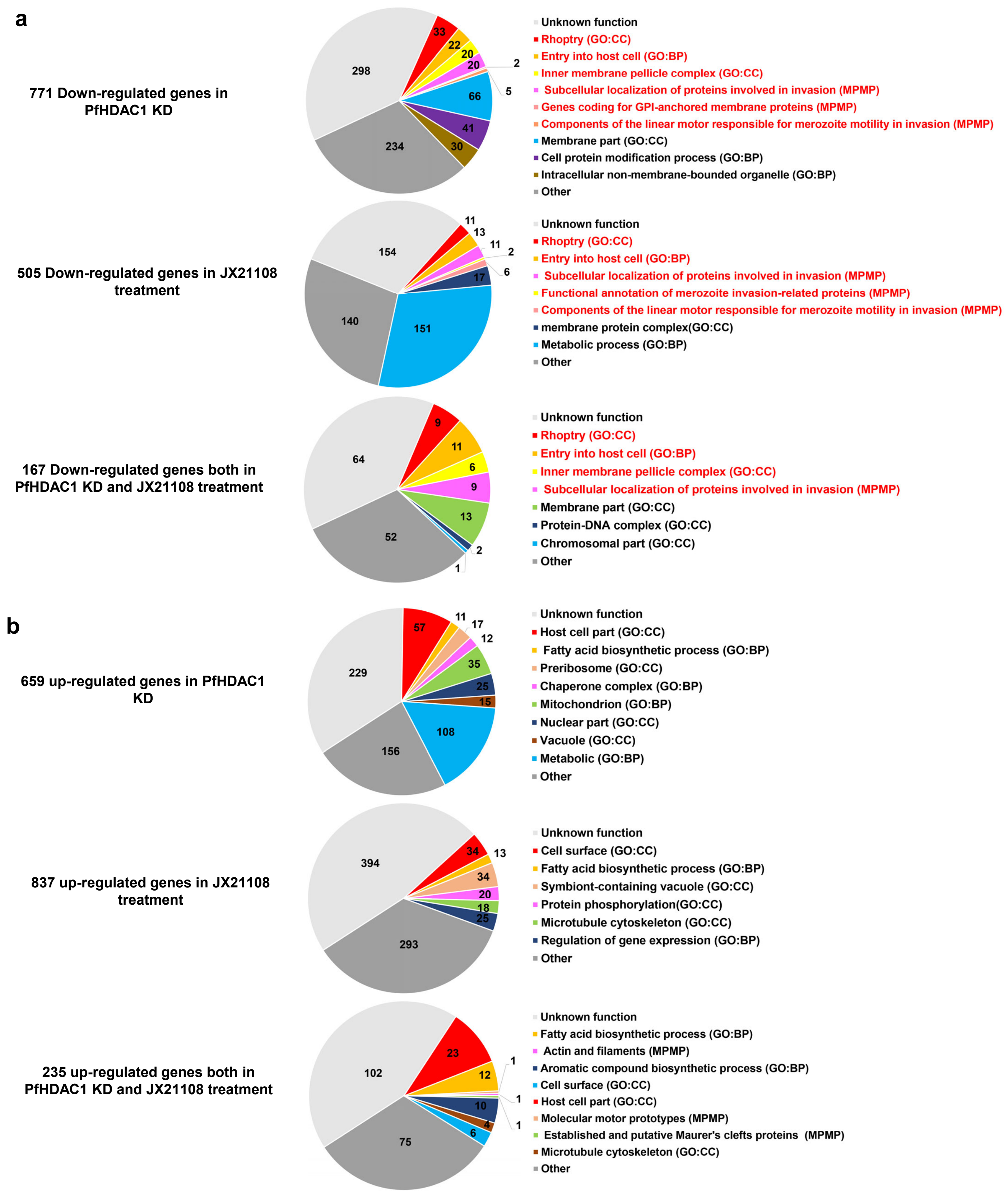

Supplementary Fig. S5 Gene Ontology (GO) analyses of genes with altered expression after PfHDAC1 knockdown (KD) or JX21108 treatment. GO analyses for downregulated genes (**a**) or u-regulated genes (**b**) from PfHDAC1 KD, JX21108 treatment or both in PfHDAC1 KD and JX21108-treated parasites. GO analyses were performed in PlasmoDB.

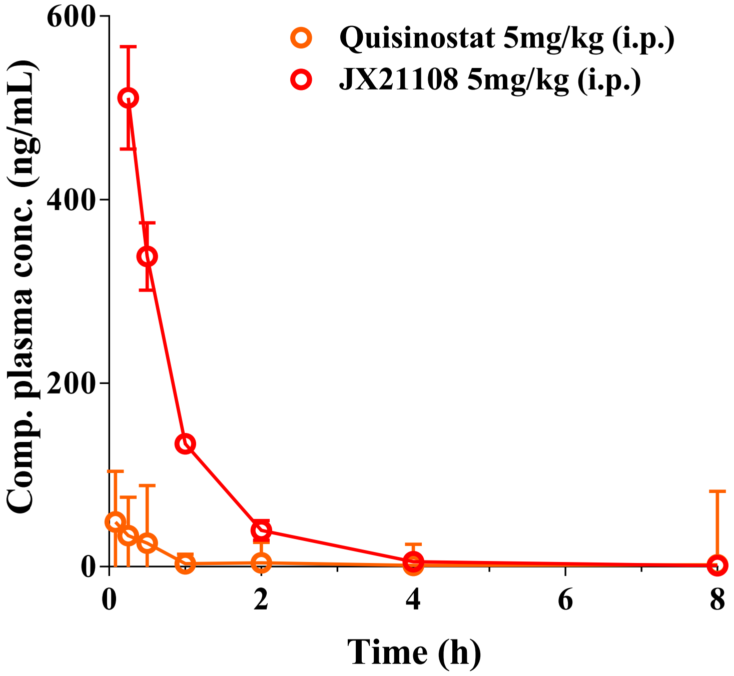

Supplementary Fig. S6 The PK curve graph of JX21108 and quisinostat in BALB/c mice related to supplementary table S4. Five mice per group were used in this experiment; i.p., intraperitoneal injection.

Supplementary Table S1. Anti-*P. falciparum* activity and cytotoxicity profiles of linker derivatives.

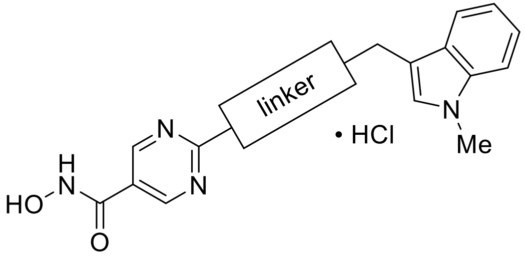

Entry	Comp.	Linker	Molecular Weight	cLog$_P$	Antiplasmodinal Activity [a], EC_{50} (nM)		Cytotoxicity [a], EC_{50} (nM)/SI			
					3D7	Dd2	HepG2	SI[b]	293T	SI[c]
1	**Quisinostat**	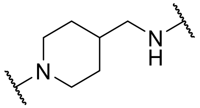	430.94	-0.98	5.2 ± 1.6	7.09 ± 0.01	42 ± 10	8	48 ± 1	9
2	**JX21002**	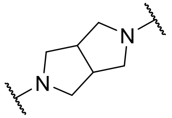	428.92	-1.19	4.4 ± 0.2	7.0 ± 0.2	128 ± 2	29	307 ± 4	70
3	**JX21003**	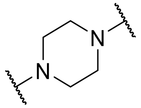	402.88	-1.79	10.9 ± 0.8	15.3 ± 0.4	44 ± 7	4	47 ± 21	4
4	**JX21004**	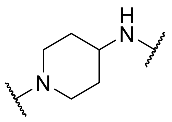	416.91	-1.36	32.9 ± 0.9	61.0 ± 0.4	294 ± 57	9	519 ± 112	16

5	**JX21005**	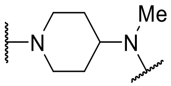	430.94	-1.26	73.6 ± 3.5	107.5 ± 5.6	643 ± 36	9	1978 ± 407	27
6	**JX21006**	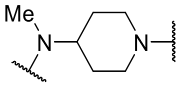	430.94	-1.26	26.5 ± 1.2	32.0 ± 2.5	37 ± 10	1	118 ± 24	4
7	**JX21008**	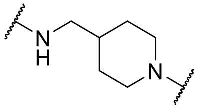	430.94	-1.24	13.1 ± 0.6	23.6 ± 0.4	688 ± 10	52	253 ± 89	19
8	**JX21022**	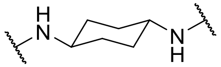	430.94	-1.20	151.1 ± 2.7	125.4 ± 4.5	>10000	>66	>10000	>66
9	**JX21024**	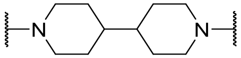	485.03	-0.11	19.8 ± 2.5	54.0 ± 3.8	871 ± 61	44	1547 ± 5	78
10	**JX21010**	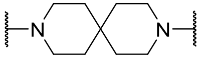	471.00	-0.78	23.4 ± 2.4	31.1 ± 4.6	904 ± 18	39	2212 ± 226	94
**11**	**JX21011**	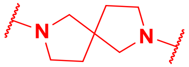	**442.95**	**-1.32**	**3.0 ± 0.7**	**3.2 ± 0.1**	**113 ± 1**	**38**	**72 ± 10**	**24**
12	**JX21013**	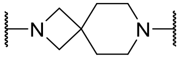	442.95	-1.32	79.2 ± 2.4	44.0 ± 10.0	>10000	>126	>10000	>126
13	**JX21014**	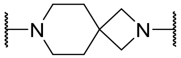	442.95	-1.32	16.9 ± 0.8	10.7 ± 0.2	348 ± 24	21	1132 ± 26	67
14	**JX21017**	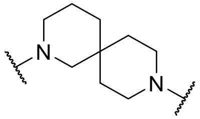	471.00	-0.78	28.9 ± 1.2	32.4 ± 1.8	2347 ± 93	98	4497 ± 33	156

15	**JX21019**	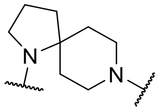	456.98	-0.79	45.2 ± 1.2	38.7 ± 0.6	875 ± 70	19	1003 ± 208	22
16	**JX21020**	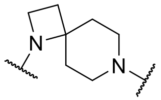	442.95	-1.06	203.1 ± 23.6	243.8 ± 47.8	1031 ± 456	5	4009 ± 223	20

[a]Results are expressed as the mean ± SD of at least two independent experiments.

[b]Selectivity index for HepG2 is defined as IC_{50} (HepG2)/IC_{50} (3D7).

[c]Selectivity index for 293T is defined as IC_{50} (293T)/IC_{50} (3D7).

$cLog_P$ was calculated from www.molinspiration.com

Supplementary Table S2. Anti *P. falciparum* activity and cytotoxicity profiles of R group derivatives.

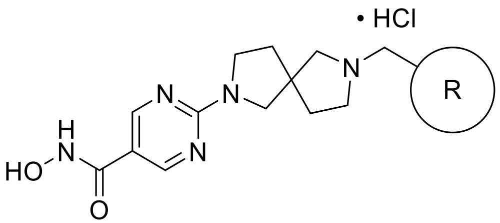

Entry	Comp.	Linker	Molecular Weight	cLogP	Antiplasmodinal Activity[a], EC50 (nM)		Cytotoxicity[a], EC50 (nM)/SI			
					3D7	Dd2	HepG2	SI[b]	293T	SI[c]
**1**	**JX21011**	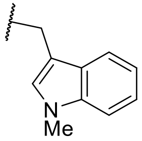	442.95	-1.32	3.0 ± 0.7	3.2 ± 0.1	113 ± 1	38	72 ± 10	24
**2**	**JX21131**	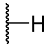	299.76	-3.23	40.4 ± 9.2	20.7 ± 2.4	> 10000	> 248	> 10000	> 248
**3**	**JX21121**	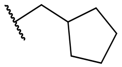	381.91	-1.35	92.5 ± 11.3	61.6 ± 4.8	> 10000	> 108	> 10000	> 108
**4**	**JX21112**	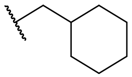	395.93	-0.84	40.4 ± 4.1	28.3 ± 3.8	2493 ± 520	62	607 ± 42	15
**5**	**JX21105**	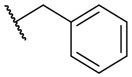	389.88	-1.54	17.8 ± 0.5	28.6 ± 1.8	1204 ± 68	68	1787 ± 37	101
**6**	**JX21119**	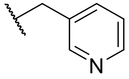	390.87	-2.77	53.3 ± 0.5	39.0 ± 4.0	6421 ± 540	120	6998 ± 57	131

**7**	**JX21111**	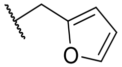	379.85	-2.28	45.5 ± 3.3	34.0 ± 2.5	1105 ± 18	24	2256 ± 286	50
**8**	**JX21136**	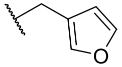	379.85	-2.59	84.7 ± 4.3	55.9 ± 2.2	> 10000	> 118	> 10000	> 118
**9**	**JX21110**	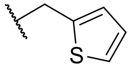	395.91	-1.64	49.7 ± 1.1	35.4 ± 1.3	1693 ± 163	34	4540 ± 323	91
**10**	**JX21120**	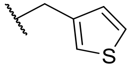	395.91	-1.95	18.6 ± 3.9	23.2 ± 1.2	2115 ± 165	114	3928 ± 374	211
**11**	**JX21123**	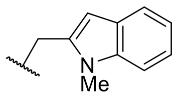	442.95	-1.01	6.2 ± 1.3	7.2 ± 0.3	1254 ± 62	202	1203 ± 156	193
**12**	**JX21103**	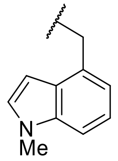	442.95	-1.29	13.9 ± 0.9	23.7 ± 1.0	1284 ± 152	93	2155 ± 81	155
**13**	**JX21135**	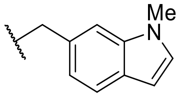	442.95	-1.27	67.4 ± 3.9	60.6 ± 7.1	7742 ± 950	115	10292 ± 1157	153
**14**	**JX21133**	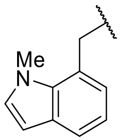	442.95	-1.29	240.8 ± 55.2	231.7 ± 9.5	> 10000	> 42	> 10000	> 42
**15**	**JX21118**	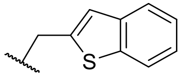	445.97	-0.33	5.61 ± 0.04	6.4 ± 1.3	1673 ± 230	298	1638 ± 122	292
**16**	**JX21117**	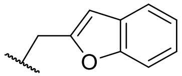	429.91	-0.97	6.4 ± 0.3	7.61 ± 0.05	1639 ± 25	255	1614 ± 334	252

**17**	**JX21115**	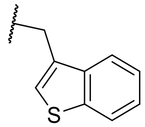	445.97	-0.64	4.8 ± 0.1	7.2 ± 0.2	864 ± 76	179	482 ± 42	100
**18**	**JX21113**	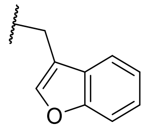	429.91	-1.28	67.5 ± 5.3	112.3 ± 13.1	> 10000	> 148	3018 ± 67	45
**19**	**JX21109**	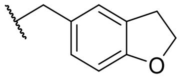	431.92	-1.51	9.8 ± 0.3	7.0 ± 0.3	1311 ± 73	133	2561 ± 113	260
**20**	**JX21140**	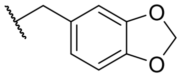	433.89	-1.65	10.5 ± 0.1	5.4 ± 0.2	493 ± 149	47	2452 ± 301	235
**21**	**JX21122**	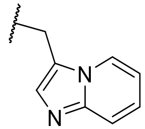	429.91	-1.93	72.9 ± 10.0	74.4 ± 27.4	> 10000	> 137	> 10000	> 137
**22**	**JX21130**	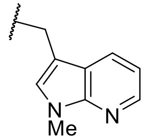	443.94	-2.22	8.4 ± 0.4	7.5 ± 2.0	1179 ± 84	140	4637 ± 375	552
**23**	**JX21129**	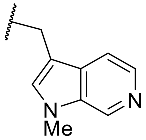	443.94	-2.41	8.0 ± 0.5	7.8 ± 1.3	1532 ± 69	192	1125 ± 98	141

**24**	**JX21134**	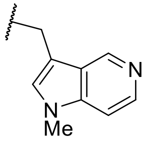	443.94	-2.41	169.9 ± 17.8	131.4 ± 11.9	> 10000	> 59	> 10000	> 59
**25**	**JX21128**	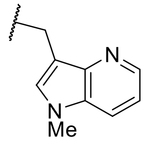	443.94	-2.22	8.7 ± 0.7	7.7 ± 0.1	>10000	>1148	>10000	>1148
**26**	**JX21124**	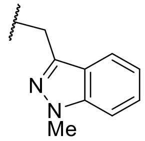	443.94	-1.54	5.8 ± 0.9	4.3 ± 0.5	1131 ± 168	195	1327 ± 29	229
**27**	**JX21141**	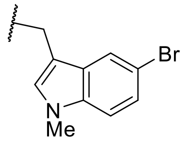	521.84	-0.53	3.3 ± 0.5	1.736±0.005	294 ± 45	88	633 ± 209	189
**28**	**JX21104**	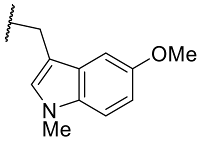	472.97	-1.29	1.6 ± 0.3	3.0 ± 0.1	534 ± 45	176	659 ± 128	217
**29**	**JX21139**	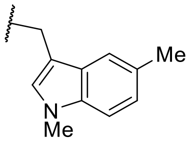	456.98	-0.89	3.1 ± 0.3	2. 1 ± 0.3	866 ± 157	283	737 ± 109	241

**30**	**JX21108**	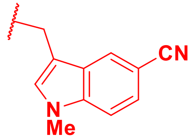	**467.96**	**-1.59**	**3.57 ± 0.06**	**4.0 ± 0.9**	**3817 ± 145**	**1069**	**1193 ± 233**	**334**
**31**	**JX21142**	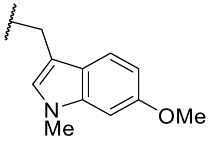	472.97	-1.29	13.6 ± 4.2	3.4 ± 2.2	975± 1	72	1229 ± 102	90
**32**	**JX21102**	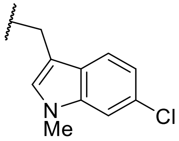	477.39	-0.66	4.2 ± 0.7	2.4 ± 0.2	514 ± 22	123	1673±110	574
**33**	**JX21106**	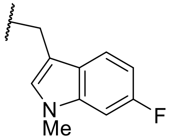	460.94	-1.18	2.0 ± 0.9	3.60 ± 0.08	178 ± 4	88	402 ± 96	198
**34**	**JX21125**	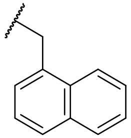	439.94	-0.38	6.0 ± 0.4	4.9 ± 0.7	815 ± 48	136	1243 ± 185	207
**35**	**JX21114**	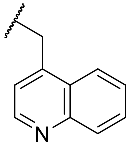	440.93	-1.30	45.9 ± 11.8	76.2 ± 9.9	3932 ± 537	86	1859 ± 12	41

**36**	**JX21101**	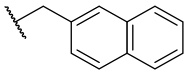	439.94	-0.35	3.5 ± 0.2	2.18 ± 0.02	651 ± 63	186	1159 ± 193	331
**37**	**JX21143**	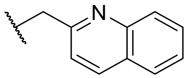	440.93	-1.46	7.9 ± 1.0	3.1 ±0.3	388 ± 58	49	2907± 655	368
**38**	**JX21137**	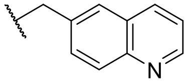	440.93	-1.55	7.0 ± 0.4	9.6 ± 0.5	1253 ± 190	179	1773 ± 28	254
**39**	**JX21138**	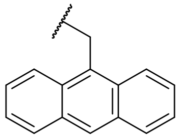	490.00	0.76	28.8 ± 18.5	21.2 ± 0.9	1308 ± 14	45	1994 ± 559	69
**40**	**JX21116**	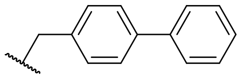	465.98	0.26	21.7 ± 0.2	33.9 ± 0.3	2961 ± 152	137	2407 ± 118	111

[a]Results are expressed as the mean ± SD of at least two independent experiments.

[b]Selectivity index for HepG2 is defined as IC_{50} (HepG2)/IC_{50} (3D7).

[c]Selectivity index for 293T is defined as IC_{50} (293T)/IC_{50} (3D7).

cLog$_P$ was calculated from www.molinspiration.com

Supplementary Table S3. Activity of Quisinostat and JX21108 to inhibit human HDACs *in vitro*

Entry	Comp.	Inhibitory Activity, IC_{50} (nM)					
		*h*HDAC1	*h*HDAC2	*h*HDAC3	*h*HDAC8	*h*HDAC6	*h*Sirt2
1	Quisinostat	0.46	1.1	2.1	9.2	35	>10000
2	JX21108	1.8	4.6	19	62	66	>10000

Supplementary Table S4. Primers used in this study

Primer name	Sequence
**Primers used in gene Knock down (5’ to 3’)**	
**P1**	GCGGCCCTAGTCTAGGGCGCGCCGTGGAATATAACAATTTCTTGAAAATAT
**P2**	GCGGCCGCGCTAGCATTCCAGAAATTTATGAATTCGAAATTC
**P3**	GCTAGCGCGGCCGCTAAACCAAATTTTAAAGCATCAAAAA
**P4**	TTTTTTTACAAAATGCTTAAGGGTAAAAGAAAATGATGTAAAAAAAC
**P5**	GCGGCCCTAGTCTAGGGCGCGCCAATATACCTTTACTCGTTCTAGGTGGTG
**P6**	GCGGCCGCGCTAGCATATGGTACAATAGATTGATCTCTGTCAGATAA
**P7**	GCTAGCGCGGCCGCGTGGAAAAATATATAACCAATGTG
**P8**	TTTTTTTACAAAATGCTTAAGGTTCCTACTTGGAACATATATATT
**P9**	ATAAATTTCTGGAATGCTAGCGGAAGTGGTTCAGGTGGA
**P10**	TTAAAATTTGGTTTCGCGGCCGCTTACTTGTCATCGTCATCCTTGT
**P11**	ATAAATTTCTGGAATGCTAGCGGCTCTGGCAGCGGCAGC
**P12**	TTAAAATTTGGTTTCGCATGCGTCCCCTCCTACATGTTTTTTG
**P13**	TCTATTGTACCATATGCTAGCGGAAGTGGTTCAGGTGGA

**P14**	TATATATTTTTCCACGCGGCCGCTTACTTGTCATCGTCATCCTTGT
**P15**	TCTATTGTACCATATGCTAGCGGCTCTGGCAGCGGCAGC
**P16**	TATATATTTTTCCACGCATGCGTCCCCTCCTACATGTTTTTTG
**P17**	ATAAATATGTTCGAACGTTATTTCTC
**P18**	AAATTATAAGAATATTAAAACGAATCGC
**P19**	ATCGATTGGGTAGATTTAATTTAAC
**P20**	TTATTTATTTACCAACTCAGAATGC
**P21**	TAAGTATATAATATTATTCATAAATTTCTGGAATTGTTTTAGAGCTAGAA
**P22**	TAAGTATATAATATTTTTTTTTGATTTATCTGACAGTTTTAGAGCTAGAA
**Primers used in RT-qPCR (5' to 3')**	
**PfHDA1-FW**	GTATGAAACCTAAAACACCTAC
**PfHDA1-RV**	AATCGTAAGGATTGTTGATTGC
**PfHDAC1-FW**	CAAAGGAACATTCAACCACAC
**PfHDAC1-RV**	GGTACAATAGATTGATCCCTG

Supplementary Table S5. The list of genes in infection term

The list of genes in Infection term			
**ID**	**Symbol**	**Description**	**function**
PF3D7_1371600	EBL1	"erythrocyte binding like protein 1, pseudogene"	invasion
PF3D7_0102500	EBA181	erythrocyte binding antigen-181	invasion
PF3D7_1301600	EBA140	erythrocyte binding antigen-140	invasion
PF3D7_0731500	EBA175	erythrocyte binding antigen-175	invasion
PF3D7_0424300	EBA165	"erythrocyte binding antigen-165, pseudogene"	invasion
PF3D7_1335300	RH2b	reticulocyte binding protein 2 homologue b	invasion
PF3D7_1252400	RH3	"reticulocyte binding protein homologue 3, pseudogene"	invasion
PF3D7_1335400	RH2a	reticulocyte binding protein 2 homologue a	invasion
PF3D7_0424200	RH4	reticulocyte binding protein homologue 4	invasion
PF3D7_0402300	RH1	reticulocyte binding protein homologue 1	invasion
PF3D7_1335200	RH6	"reticulocyte binding protein homologue 6, pseudogene"	invasion
PF3D7_0424100	RH5	reticulocyte binding protein homologue 5	invasion
PF3D7_0323400	RIPR	Rh5 interacting protein	invasion
PF3D7_1035500	MSP6	merozoite surface protein 6	invasion
PF3D7_1035700	DBLMSP	duffy binding-like merozoite surface protein	invasion
PF3D7_1036300	DBLMSP2	duffy binding-like merozoite surface protein 2	invasion
PF3D7_1035400	MSP3	merozoite surface protein 3	invasion
PF3D7_0502400	MSP8	merozoite surface protein 8	invasion
PF3D7_1036000	MSP11	merozoite surface protein	invasion
PF3D7_0620400	MSP10	merozoite surface protein 10	invasion
PF3D7_1335100	MSP7	merozoite surface protein 7	invasion
PF3D7_0930300	MSP1	merozoite surface protein 1	invasion
PF3D7_0206900	MSP5	merozoite surface protein 5	invasion
PF3D7_0207000	MSP4	merozoite surface protein 4	invasion
PF3D7_0206800	MSP2	merozoite surface protein 2	invasion
PF3D7_1228600	MSP9	merozoite surface protein 9	invasion
PF3D7_0613900	myoE	"myosin E, putative"	invasion
PF3D7_1229800	MyoD	myosin D	invasion
PF3D7_0503600	MyoB	myosin B	invasion
PF3D7_1342600	MyoA	myosin A	invasion
PF3D7_1329100	MyoC	myosin C	invasion
PF3D7_1140500	MyoF	"myosin F, putative"	invasion
PF3D7_1246400	MTIP	myosin A tail domain interacting protein	invasion
PF3D7_1361800	GAC	glideosome-associated	invasion
PF3D7_1222700	GAP45	glideosome-associated protein	invasion
PF3D7_0722200	RALP1	rhoptry-associated leucine zipper-like protein 1	invasion
PF3D7_0707300	RAMA	rhoptry-associated membrane antigen	invasion
PF3D7_0214900	RON6	rhoptry neck protein 6	invasion
PF3D7_0414900	ARO	armadillo-domain containing rhoptry protein	invasion
PF3D7_0613300	ROP14	rhoptry protein ROP14	invasion

PF3D7_1452000	RON2	rhoptry neck protein 2	invasion
PF3D7_1116000	RON4	rhoptry neck protein 4	invasion
PF3D7_0817700	RON5	rhoptry neck protein 5	invasion
PF3D7_1252100	RON3	rhoptry neck protein 3	invasion
PF3D7_1012200	RA	rhoptry associated adhesin	invasion
PF3D7_1410400	RAP1	rhoptry-associated protein 1	invasion
PF3D7_1017100	RON12	rhoptry neck protein 12	invasion
PF3D7_0905400	RhopH3	high molecular weight rhoptry protein 3	invasion
PF3D7_0929400	RhopH2	high molecular weight rhoptry protein 2	invasion
PF3D7_0501600	RAP2	rhoptry-associated protein 2	invasion
PF3D7_0511600	ARNP	apical rhoptry neck protein	invasion
PF3D7_0501500	RAP3	rhoptry-associated protein 3	invasion
PF3D7_1466900	N/A	"rhoptry protein, putative"	invasion
PF3D7_1366400	RHOP148	rhoptry protein RHOP148	invasion
PF3D7_1133400	AMA1	apical membrane antigen 1	invasion
PF3D7_0828800	GAMA	GPI-anchored micronemal	invasion
PF3D7_0316000	MA	microneme associated antigen	invasion
PF3D7_1030200	CLAMP	"claudin-like apicomplexan microneme protein, putative"	invasion
PF3D7_0404700	DPAP3	dipeptidyl aminopeptidase 3	invasion
PF3D7_0423400	AARP	apical asparagine-rich protein AARP	invasion
PF3D7_1431400	SRA	"conserved Plasmodium protein, unknown function"	invasion
PF3D7_1238900	pk2	protein kinase 2	invasion
PF3D7_0528400	DHHC3	palmitoyltransferase DHHC7	invasion
PF3D7_1337800	CDPK5	calcium-dependent protein kinase 5	egeress
PF3D7_0217500	CDPK1	calcium-dependent protein kinase 1	egeress
PF3D7_1136900	SUB2	subtilisin-like protease 2	egeress
PF3D7_0507500	SUB1	subtilisin-like protease 1	egeress
PF3D7_1028700	MTRAP	merozoite TRAP-like protein	egeress
PF3D7_1436600	PKG	cGMP-dependent protein kinase	egeress
PF3D7_0808200	plasmepsin X	plasmepsin X	egeress

Supplementary Table S6. Quality control information of RNAseq

Quality control information of RNAseq				
sample name	raw reads	paired reads	aligned concordantly exactly 1 time	aligned concordantly >1 times
3D7-DMSO-Schizont-rep1	24655275	24652825	20702280	608339
3D7-DMSO-Schizont-rep2	21979048	21977179	18574575	451470
3D7HDAC1-glms-Glcn-Schizont-rep1	27320353	27319574	24820322	732113
3D7HDAC1-glms-Glcn-Schizont-rep2	20832241	20831010	18054228	524288
3D7HDAC1-Ty1-Glcn-Schizont-rep1	27310681	27309676	24240205	856501
3D7HDAC1-Ty1-Glcn-Schizont-rep2	20678886	20677064	17548123	999342
3D7-JX21108-Schizont-rep1	23735453	23733167	20005169	438247
3D7-JX21108-Schizont-rep2	24727183	24725140	20722299	457828

Supplementary Materials and Methods

Chemical Synthesis and analytical data

Abbreviation

Ac	acetyl
Boc	*t*-butoxycarbonyl
calcd.	calculated
Cbz	benzyloxycarbonyl
DCM	dichloromethane
DCE	dichloroethane
DIPEA	*N*,*N*-diisoprapylethylamine
DMF	*N*,*N*-dimethylformadine
DMSO	dimethylsulfoxide
EDCI	1-(3-dimethylaminopropyl)-3-ethylcarbodiimide hydrochloride
EA	ethyl acetate
Et	ethyl
ESI	electrospray ionization
h	hour
HOBt	1-Hydroxybenzotriazole
HRMS	high-resolution mass spectra
Me	methyl
min	minute
NMR	nuclear magnetic resonance
PE	petroleum ether
rt	room temperature
TEA	triethylamine
THF	tethahydrofuran
THP	2-tetrahydropyranyl
TLC	thin-layer chromatography

Chemical Synthesis

JL01 sample was purchased from WuXi AppTec. Unless otherwise noted, reagents and solvents were purchased from commercial suppliers and were directly used without further purification. Flash column chromatography was performed using HSGF 254 (150–200 µm thickness; Yantai Huiyou Co., China). Analytical thin-layer chromatography (TLC) was performed using HSGF 254 (150–200 µm thickness; Yantai Huiyou Co., China), and spots were visualized with UV light, iodine, and potassium permanganate staining. Reaction conditions were not optimized. Chiral column chromatography was conducted in Daicel Chiral Technologies (China) CO. Nuclear magnetic resonance (NMR) spectroscopy was performed on a Bruker AMX-400 NMR (IS as TMS). Chemical shifts were reported in parts per million (ppm, *δ*) downfield from tetramethylsilane. Proton coupling patterns were described as singlet (s), doublet (d), triplet (t), quartet (q), multiplet (m), and broad (br). High-resolution mass spectra (HRMS) was obtained by electrospray ionization (ESI) using a Waters GCT Premie and Waters LCT. HPLC

analysis was performed on Agilent 1100 with quaternary pump and diode-array detector (DAD), using Agilent Exlipse XDB-C18 (250×4.6 mm, 5 μm particle size) column. Eluent was MeOH/aq. H_3PO_4 (0.1%, 90/10), flow rate was 0.5 ml/min, and peak purity was checked with UV spectra. Optical activity of enantiopure compound was measured via Rudolph Research Analytical AUTOPOL V automatic polarimeter.

Synthesis of linker derivatives[a, b]

Generally, preparation of linker derivatives is based on the synthetic procedure of **JL01**[1] using corresponding **H-L-Boc** as starting material. H-L-Boc were purchased from commercial suppliers.

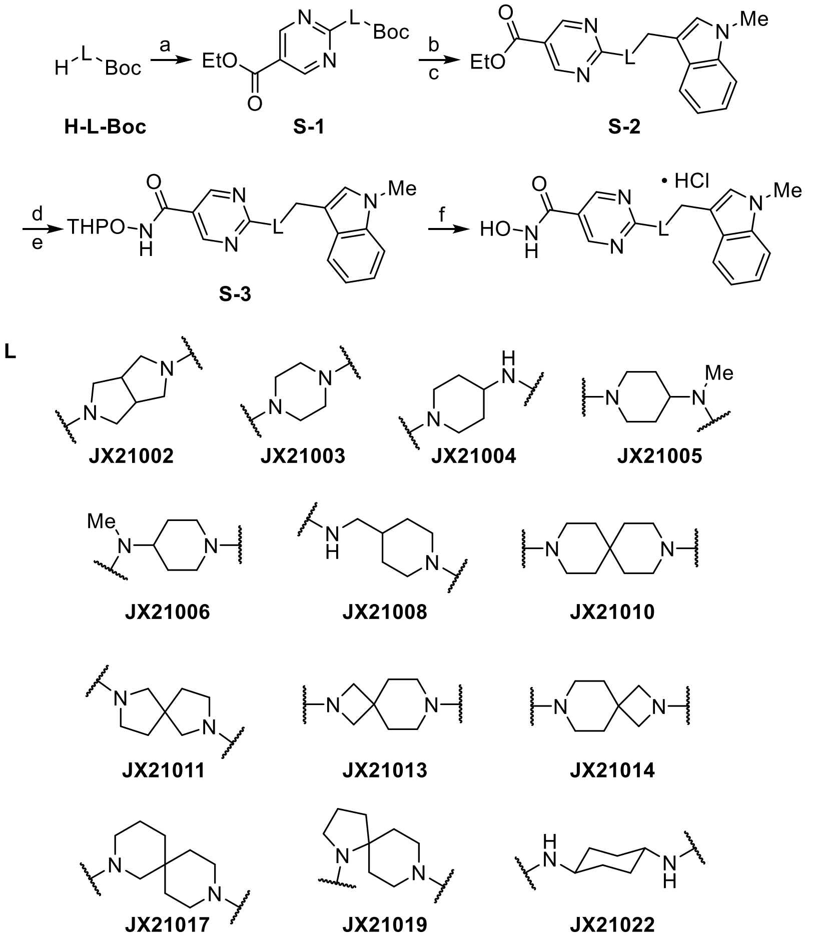

Scheme 1. [a]Reagents and conditions: (a) ethyl 2-chloropyrimidine-5-carboxylate, DIPEA, DCM, rt, 3-6 h; (b) HCl/1, 4-dioxane (4 M), DCM, rt, 2-5 h; (c) 1-methyl-3-formylindole, $NaBH(OAc)_3$, HOAc, DCE, rt, 12 h; (d) NaOH, THF, H_2O, reflux; (e) $THPONH_2$, EDCI, HOBt, TEA, DMF, rt, 72 h; (f) HCl/1, 4-dioxane (4 M), DCM, rt, 30 min. [b]In the substrate **H-L-Boc** the right-side nitrogen atom of L link to Boc group.

General synthetic menthod of compounds JX21002-JX21022

General synthesis of **S-1**:

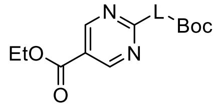

To a mixture of corresponding **H-L-Boc** (1 g) and ethyl 2-chloropyrimidine-5-carboxylate (1.2 eq.) in DCM (0.2 M for **H-L-Boc**) was added DIPEA (1.5 eq.) in one portion at 0 °C under N_2. The mixture was stirred at rt for 3 h. TLC (PE/EA = 2/1) showed the reaction was complete. The reaction mixture was concentrated under reduced pressure to remove DCM. The residue was purified by flash column chromatography (SiO_2, PE/EA) to get compound **S-1**.

General synthesis of **S-2**:

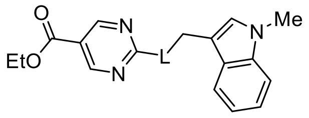

To a solution of compound **S-1** (1 eq) in DCM (0.5 M) was added HCl/1, 4-dioxane (4 M, 6 eq.). The mixture was stirred at rt for 2 h. TLC (PE/EA = 2/1) showed the reaction was complete. The solvent was removed in vacuo. The reaction mixture was washed with saturated $NaHCO_3$ and filtered. The resulted precipitate was wash with H_2O for 3 times, dried in vacuo to get deprotected mixture which was used directly in the next steps without purification.

To the mixture of deprotected mixture (considered as 1 eq.), *N*-methyl-3-formylindole (1.2 eq.) was added DCE (0.2 M) and HOAc (2 eq.) under N_2. The mixture was stirred at rt for 30 min. $NaBH(OAc)_3$ (1.5 eq.) was then added and the mixture stirred for 12 h. TLC (DCM/MeOH = 9/1) showed the reaction was complete. The reaction mixture was quenched by addition saturated aqueous $NaHCO_3$, and then the reaction mixture was diluted with $H_2O$and extracted three times with DCM. The combined organic layers were dried over Na_2SO_4, filtered and concentrated under reduced pressure. The residue was purified by flash column chromatography (SiO_2, DCM/MeOH) to get compound **S-2**.

General synthesis of **S-3**:

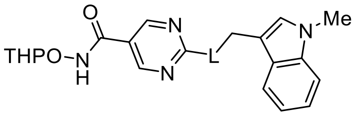

To a solution of compound **S-2** (1 eq.) in THF (0.1 M) was added aqueous NaOH (1M, 10 eq.). The mixture was stirred at reflux until TLC (DCM/MeOH = 9/1) showed the reaction was complete. The mixture was then acidified to pH = 2~3 with 2 M HCl, causing a solid to precipitate. This solid was collected, washed with large amount of H_2O to pH = ca.7 and dried to get hydrolyzed mixture which was used directly in the next steps without purification.

To the hydrolyzed mixture (considered as 1 eq.) was stirred with EDCI (3 eq.) and HOBt (3 eq.) in DMF (0.2 M) at rt under a nitrogen atmosphere for 30 min. $THPONH_2$ (5 eq.) was then added followed by TEA (5 eq.) and the mixture allowed to stirred at rt for 72 h. TLC (DCM/MeOH = 9/1) showed the reaction was complete. The mixture was then diluted with H_2O and extracted three times with DCM. The combined organic layers were wash twice with H_2O, once with brine, dried (Na_2SO_4), and the solvent was removed in vacuo. The residue was purified by column chromatography (SiO_2, DCM/MeOH) to get

compound **S-3**.

General synthesis of compounds **JX21002-JX21022**

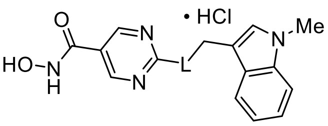

To a solution of compound **S-3** (1 eq.) in DCM (0.1 M) was added HCl/dioxane (4 M, 2.5 eq.). The mixture was stirred at rt for 30 min under a nitrogen atmosphere. TLC showed the reaction was complete. The reaction mixture was filtered and the resulted precipitate was wash with large amount of DCM and ether, dried in vacuo to get compound.

***N*-Hydroxy-2-(5-((1-methyl-1*H*-indol-3-yl)methyl)hexahydropyrrolo[3,4-c]pyrrol-2(1*H*)-yl)pyrimidine-5-carboxamide hydrochloride (JX21002)**

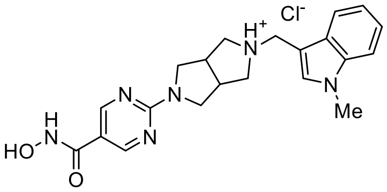

Compound **JX21002** was synthesized according to the above general synthetic method.
112 mg (5.5% yield from **H-L-Boc**); ^{1}H-NMR (400 MHz, DMSO-d_6) δ 11.19 (m, 1H), 8.71 (m, 2H), 7.85 (t, J=7.8 Hz, 1H), 7.51–7.43 (m, 1H), 7.22 (q, J=7.6 Hz, 1H), 7.13 (dt, J=11.5, 7.5 Hz, 1H), 4.52 (m, 2H), 3.81 (m Hz, 4H), 3.75 (m, 1H), 3.70–3.53 (m, 3H), 3.41 (m, 2H), 3.25 (m, 1H), 3.09 (m, 1H), 3.01 (m, 1H); HRMS (ESI) m/z calcd. for $C_{21}H_{23}N_6O_2$ [M-H]$^+$ 391.1882, found 391.1884; Purity: 97% (254 nm, RT = 4.066 min)

***N*-Hydroxy-2-(4-((1-methyl-1*H*-indol-3-yl)methyl)piperazin-1-yl)pyrimidine-5-carboxamide hydrochloride (JX21003)**

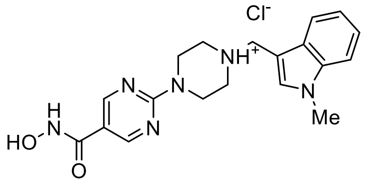

Compound **JX21003** was synthesized according to the above general synthetic method.
101 mg (4.7% yield from **H-L-Boc**); ^{1}H NMR (400 MHz, DMSO-d_6) δ 11.19 (s, 1H), 10.47–10.35 (m, 1H), 9.08 (s, 1H), 8.74 (s, 2H), 7.81 (d, J=7.9 Hz, 1H), 7.60 (s, 1H), 7.51 (d, J=8.2 Hz, 1H), 7.24 (t, J=7.6 Hz, 1H), 7.16 (t, J=7.4 Hz, 1H), 4.80 (m, 2H), 4.49 (m, 2H), 3.84 (s, 3H), 3.50 (m, 2H), 3.33 (m, 2H), 3.10 (m, 2H); HRMS (ESI) m/z calcd. for $C_{19}H_{23}N_6O_2$ [M+H]$^+$ 367.1882, found 367.1883; Purity: 99% (254 nm, RT = 4.017 min).

***N*-Hydroxy-2-(4-(((1-methyl-1*H*-indol-3-yl)methyl)amino)piperidin-1-yl)pyrimidine-5-carboxamide hydrochloride (JX21004)[2]**

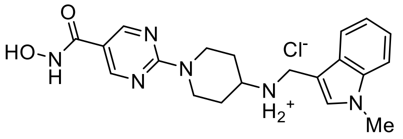

Compound **JX21004** was synthesized according to the above general synthetic method.
103 mg (4.9% yield from **H-L-Boc**); ^{1}H NMR (400 MHz, DMSO-d_6) δ 9.32 (m, 1H), 8.71 (s, 2H), 7.81 (d, J=7.9 Hz, 1H), 7.63 (s, 1H), 7.48 (d, J=8.2 Hz, 1H), 7.22 (t, J=7.6 Hz, 1H), 7.13 (t, J=7.4 Hz, 1H), 4.79 (m, 2H), 4.32 (m, 2H), 3.81 (s, 3H), 3.40 (m, 1H), 2.99 (m, 2H), 2.38–2.14 (m, 2H), 1.63 (m, 2H); HRMS (ESI) m/z calcd. for $C_{20}H_{25}N_6O_2$ [M+H]$^+$ 381.2039, found 381.2038; Purity: 98% (254 nm, RT = 4.055 min)

***N*-Hydroxy-2-(4-(methyl((1-methyl-1*H*-indol-3-yl)methyl)amino)piperidin-1-yl)pyrimidine-5-carboxamide hydrochloride (JX21005)**

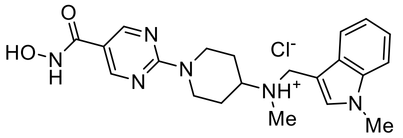

Compound **JX21005** was synthesized according to the above general synthetic method.
110 mg (5.5% yield from **H-L-Boc**); ^{1}H NMR (400 MHz, DMSO-d_6) δ 10.63 (s, 1H), 8.74 (s, 2H), 7.81 (d, J=7.9 Hz, 1H), 7.71 (s, 1H), 7.50 (d, J=8.2 Hz, 1H), 7.23 (t, J=7.6 Hz, 1H), 7.14 (t, J=7.4 Hz, 1H), 4.89 (m, 2H), 4.57 (m, 1H), 4.38 (m, 1H), 3.83 (s, 3H), 3.57 (m, 1H), 2.94 (m, 2H), 2.61 (m , 3H), 2.35 (m, 1H), 2.24 (m, 1H), 1.77 (m, 2H); HRMS (ESI) m/z calcd. for $C_{21}H_{27}N_6O_2$ [M+H]$^+$ 395.2195, found 395.2194; Purity: 99% (254 nm, RT = 3.978 min).

***N*-Hydroxy-2-(methyl(1-((1-methyl-1*H*-indol-3-yl)methyl)piperidin-4-yl)amino)pyrimidine-5-carboxamide hydrochloride (JX21006)**

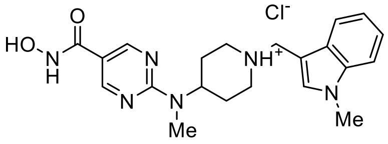

Compound **JX21006** was synthesized according to the above general synthetic method. 108 mg (5.4% yield from **H-L-Boc**); ^{1}H NMR (400 MHz, DMSO-d_6) δ 11.13 (s, 1H), 10.27 (s, 1H), 8.69 (s, 2H), 7.83 (d, J=7.9 Hz, 1H), 7.61 (s, 1H), 7.51 (d, J=8.2 Hz, 1H), 7.24 (t, J=7.5 Hz, 1H), 7.16 (t, J=7.4 Hz, 1H), 4.87 (m, 1H), 4.44 (m, 2H), 3.84 (s, 3H), 3.57 – 3.45 (m, 3H), 3.24 – 3.11 (m, 2H), 2.99 (s, 3H), 2.17 (m, 2H), 1.79 (m, 2H). HRMS (ESI) m/z calcd. for $C_{21}H_{27}N_6O_2$ [M+H]$^+$ 395.2195, found 395.2196. Purity: 99% (254 nm, RT = 4.049 min)

N-hydroxy-2-(((1-((1-methyl-1H-indol-3-yl)methyl)piperidin-4-yl)methyl)amino)pyrimidine-5-carboxamide hydrochloride (JX21008)

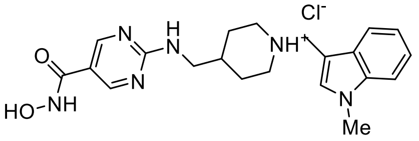

Compound **JX21008** was synthesized according to the above general synthetic method.

116 mg (5.8% yield from **H-L-Boc**); ^{1}H NMR (400 MHz, DMSO-d_6) δ 10.06 (s, 1H), 8.76–8.33 (m, 2H), 7.91 (t, 1H), 7.87–7.75 (m, 1H), 7.60 (m, 1H), 7.49 (d, J=8.2 Hz, 1H), 7.22 (t, J=7.5 Hz, 1H), 7.13 (t, J=7.3 Hz, 1H), 4.38 (m, 2H), 3.83 (m, 3H), 3.41 (m, 2H), 3.20 (m, 2H), 2.97 (m, 1H), 2.88 (m, 2H), 1.81 (m, 3H), 1.48 (m, 2H); HRMS (ESI) m/z calcd. for $C_{21}H_{27}N_6O_2$ $[M+H]^+$ 395.2195, found 395.2194; Purity: 99% (254 nm, RT = 4.004 min)

***N*-Hydroxy-2-(9-((1-methyl-1*H*-indol-3-yl)methyl)-3,9-diazaspiro[5.5]undecan-3-yl)pyrimidine-5-carboxamide hydrochloride (JX21010)**

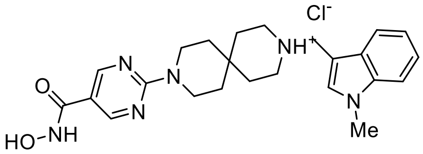

Compound **JX21010** was synthesized according to the above general synthetic method.

123 mg (6.6% yield from **H-L-Boc**); ^{1}H NMR (400 MHz, DMSO-d_6) δ 10.24 (s, 1H), 8.67 (s, 2H), 7.86 (d, J=7.9 Hz, 1H), 7.62 (s, 1H), 7.50 (d, J=8.2 Hz, 1H), 7.23 (t, J=7.6 Hz, 1H), 7.15 (t, J=7.4 Hz, 1H), 4.44 (m, 2H), 3.82 (m, 7H), 3.23 (m, 2H), 3.12 (m, 2H), 1.87 (m, 2H), 1.76–1.62 (m, 4H), 1.35 (m, 2H); HRMS (ESI) m/z calcd. for $C_{24}H_{31}N_6O_2$ $[M+H]^+$ 435.2508, found 435.2507; Purity: 99% (254 nm, RT = 4.068 min).

***N*-Hydroxy-2-(7-((1-methyl-1*H*-indol-3-yl)methyl)-2,7-diazaspiro[4.4]nonan-2-yl)pyrimidine-5-carboxamide hydrochloride (JX21011)**

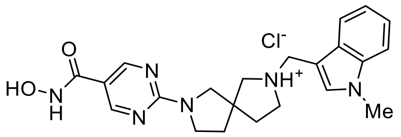

Compound **JX21011** was synthesized according to the above general synthetic method.

129 mg (6.6% yield from **H-L-Boc**); ^{1}H NMR (400 MHz, DMSO-d_6) δ 11.11 (m 1H), 8.69 (m, 2H), 7.86 (d, J=8.0 Hz, 1H), 7.65 (s, 1H), 7.49 (d, J=8.2 Hz, 1H), 7.22 (td, J=7.7, 3.1 Hz, 1H), 7.14 (q, J=7.4 Hz, 1H), 4.52 (m, 2H), 3.82 (s, 3H), 3.67–3.39 (m, 6H), 3.33 (m, 1H), 3.23 (m, 1H), 2.05 (m, 4H); ^{13}C NMR (151 MHz, DMSO) δ 161.93, 161.88, 159.84, 159.80, 157.07, 156.80, 136.40, 136.37, 132.32, 132.29, 127.45, 127.41, 121.78, 121.75, 119.68, 119.64, 118.87, 118.83, 114.40, 110.12, 103.13, 103.10, 66.36, 59.52, 59.03, 56.57, 56.15, 51.84, 51.80, 48.11, 47.96, 47.16, 47.13, 45.75, 45.69, 35.56, 35.21, 33.80, 33.44, 32.71; HRMS (ESI) m/z calcd. for $C_{22}H_{27}N_6O_2$ $[M+H]^+$ 407.2195, found 407.2196; Purity: 96% (254 nm, RT = 4.052 min).

***N*-Hydroxy-2-(7-((1-methyl-1*H*-indol-3-yl)methyl)-2,7-diazaspiro[3.5]nonan-2-yl)pyrimidine-5-carboxamide hydrochloride (JX21013)**

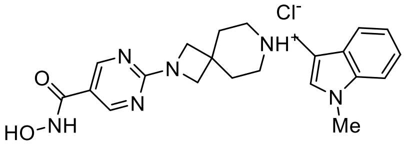

Compound **JX21013** was synthesized according to the above general synthetic method.
132 mg (6.7% yield from **H-L-Boc**); ^{1}H NMR (400 MHz, DMSO-*d*$_6$) δ 10.65 (s, 1H), 8.68 (s, 2H), 7.86 (d, *J*=7.9 Hz, 1H), 7.64 (s, 1H), 7.50 (d, *J*=8.2 Hz, 1H), 7.23 (t, *J*=7.6 Hz, 1H), 7.14 (t, *J*=7.4 Hz, 1H), 4.41 (m, 2H), 3.93 (s, 2H), 3.83 (s, 5H), 3.33 (m, 2H), 3.01 (m, 2H), 2.20–1.98 (m, 4H); HRMS (ESI) m/z calcd. for $C_{22}H_{27}N_6O_2$ [M+H]$^+$ 407.2195, found 407.2200; Purity: 99% (254 nm, RT = 3.995 min).

***N*-Hydroxy-2-(2-((1-methyl-1*H*-indol-3-yl)methyl)-2,7-diazaspiro[3.5]nonan-7-yl)pyrimidine-5-carboxamide hydrochloride (JX21014)**

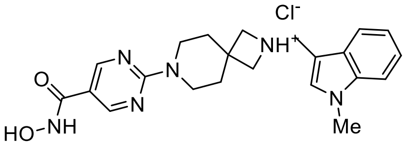

Compound **JX21014** was synthesized according to the above general synthetic method.
135 mg (6.9% yield from **H-L-Boc**); ^{1}H NMR (400 MHz, DMSO-*d*$_6$) δ 11.20 (m, 1H), 8.68 (s, 2H), 7.89 (d, *J*=7.9 Hz, 1H), 7.66 (s, 1H), 7.48 (d, *J*=8.2 Hz, 1H), 7.22 (t, *J*=7.6 Hz, 1H), 7.13 (t, *J*=7.4 Hz, 1H), 4.53 (m, 2H), 3.95–3.77 (m, 10H), 3.72 (m, 2H), 1.90 (m, 2H), 1.79 (m, 2H); HRMS (ESI) m/z calcd. for $C_{22}H_{27}N_6O_2$ [M+H]$^+$ 407.2195, found 407.2196; Purity: 97% (254 nm, RT = 4.039 min).

***N*-Hydroxy-2-(9-((1-methyl-1*H*-indol-3-yl)methyl)-2,9-diazaspiro[5.5]undecan-2-yl)pyrimidine-5-carboxamide hydrochloride (JX21017)**

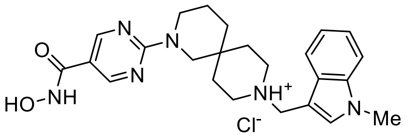

Compound **JX21017** was synthesized according to the above general synthetic method.
113 mg (6.1% yield from **H-L-Boc**); ^{1}H NMR (400 MHz, DMSO-*d*$_6$) δ 10.81–10.63 (m, 1H), 8.66 (m, 2H), 7.89 (dd, *J*=13.6, 7.9 Hz, 1H), 7.66 (d, *J*=8.6 Hz, 1H), 7.49 (dd, *J*=12.0, 8.2 Hz, 1H), 7.27–7.19 (m, 1H), 7.14 (dt, *J*=15.4, 7.5 Hz, 1H), 4.44 (m, 2H), 3.82 (m, 6H), 3.61 (s, 1H), 3.20 (m, 3H), 3.06 (m, 1H), 1.80–1.49 (m, 8H), 1.44 (m, 1H). HRMS (ESI) m/z calcd. for $C_{24}H_{31}N_6O_2$ [M+H]$^+$ 435.2508, found 435.2509. Purity: 99% (254 nm, RT = 4.031 min)

***N*-Hydroxy-2-(8-((1-methyl-1*H*-indol-3-yl)methyl)-1,8-diazaspiro[4.5]decan-1-yl)pyrimidine-5-carboxamide hydrochloride (JX21019)**

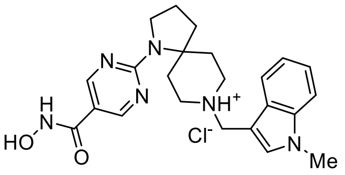

Compound **JX21019** was synthesized according to the above general synthetic method.
98 mg (5.2% yield from **H-L-Boc**); ^{1}H NMR (400 MHz, DMSO-d_6) δ 10.12 (s, 1H), 8.71 (s, 2H), 7.90 (m, 1H), 7.73 (m, 1H), 7.51 (dd, *J*=8.5, 5.1 Hz, 1H), 7.23 (t, *J*=7.6 Hz, 1H), 7.15 (t, *J*=7.5 Hz, 1H), 4.62 (m, 2H), 3.85 (s, 3H), 3.63 (m, 2H), 3.43–3.18 (m, 6H), 2.16 (m, 2H), 1.86 (m, 2H), 1.53 (m, 2H); HRMS (ESI) m/z calcd. for $C_{23}H_{29}N_6O_2$ $[M+H]^+$ 421.2352, found 421.2354; Purity: 99% (254 nm, RT = 4.059 min).

***N*-Hydroxy-2-(((1*R*,4*R*)-4-(((1-methyl-1H-indol-3-yl)methyl)amino)cyclohexyl)amino)pyrimidine-5-carboxamide hydrochloride (JX21022)**

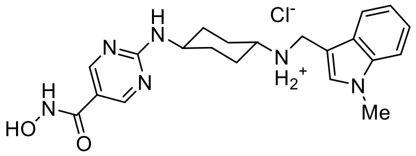

Compound **JX21022** was synthesized according to the above general synthetic method.
126 mg (6.3% yield from **H-L-Boc**); ^{1}H NMR (400 MHz, DMSO-d_6) δ 9.22 (s, 1H), 8.67 (s, 2H), 8.10–7.96 (m, 1H), 7.80 (d, *J*=7.9 Hz, 1H), 7.63 (s, 1H), 7.48 (d, *J*=8.2 Hz, 1H), 7.22 (t, *J*=7.6 Hz, 1H), 7.13 (t, *J*=7.5 Hz, 1H), 4.30 (m, 2H), 3.82 (m, 4H), 3.10–2.96 (m, 1H), 2.25 (m, 2H), 2.08–1.93 (m, 2H), 1.62 (m, 2H), 1.40–1.26 (m, 2H); HRMS (ESI) m/z calcd. for $C_{21}H_{27}N_6O_2$ $[M+H]^+$ 395.2195, found 395.2196; Purity: 96% (254 nm, RT = 4.129 min).

2. Synthesis of R derivatives[a]

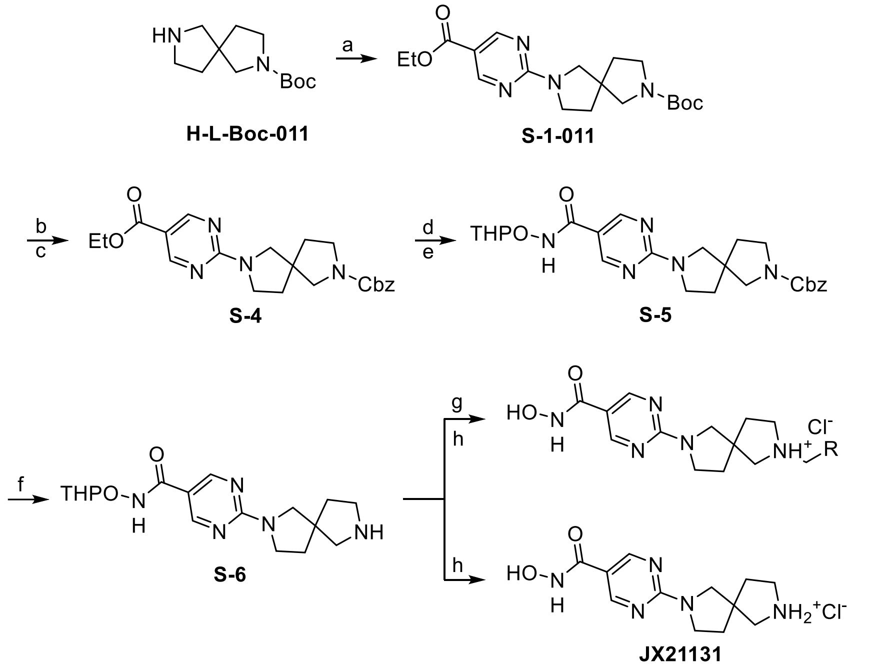

**JX21101 R = 2-naphthyl**	**JX21119 R = 3-pyridyl**
**JX21102 R = 1-methyl-6-chloro-3-indonyl**	**JX21120 R = 3-thienyl**
**JX21104 R = 1-methyl-5-methoxy-3-indonyl**	**JX21121 R = cyclopentyl**
**JX21105 R = phenyl**	**JX21122 R = 3-imidazo[1,2-a]pyridyl**
**JX21106 R = 1-methyl-6-fluoro-3-indonyl**	**JX21123 R = 1-methyl-2-indonyl**
**JX21108 R = 1-methyl-5-cyno-3-indonyl**	**JX21124 R = 1-methyl-3-indazyl**
**JX21109 R = 2,3-dihydro-5-benzofuryl**	**JX21125 R = 1-naphthyl**
**JX21110 R = 2-thienyl**	**JX21129 R = 1-methyl-3-pyrrolo[2,3-c]pyridyl**
**JX21111 R = 2-furyl**	**JX21130 R = 1-methyl-3-pyrrolo[2,3-b]pyridyl**
**JX21112 R = cyclohexyl**	**JX21137 R = 6-quinolyl**
**JX21113 R = 3-benzofuryl**	**JX21138 R = 9-anthracyl**
**JX21114 R = 4-quinolyl**	**JX21140 R = 3,4-(methylenedioxy)benzyl**
**JX21115 R = 3-benzothienyl**	**JX21141 R = 1-methyl-5-bromo-3-indonyl**
**JX21116 R = 4-phenylphenyl**	**JX21142 R = 1-methyl-6-methoxy-3-indonyl**
**JX21117 R = 2-benzofuryl**	**JX21143 R = 2-quinolyl**
**JX21118 R = 2-benzothienyl**	

Scheme 2. Synthesis of R derivatives. [a]Reagents and conditions: (a) ethyl 2-chloropyrimidine-5-carboxylate, DIPEA, DCM, rt, 6 h; (b) HCl/1, 4-dioxane (4 M), DCM, rt, 0.5 h; (c) Cbz-Cl, DIPEA, DCM, rt, 6 h; (d) K_2CO_3, MeOH, H_2O, 65 °C, 12 h; (e) $THPONH_2$, EDCI, HOBt, TEA, DMF, rt, 72 h; (f) Et_3SiH, Pd/C, MeOH, DCM, rt, 2 h; (g) R-CHO, $NaBH_3(CN)$, MeOH, rt, 24 h; (h) HCl/1, 4-dioxane 1, 4-dioxane(4 M), DCM, rt, 30 min.

General synthetic method of compounds **JX21101-JX21143**

Synthesis of **S-1-011**

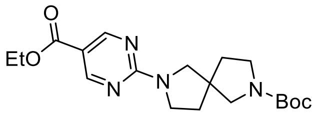

To a mixture of **H-L-Boc-011** (6 g, 26.5 mmol) and ethyl 2-chloropyrimidine-5-carboxylate (5.9 g, 32 mmol 1.2 eq.) in DCM (130 mL, 0.2 M for **H-L-Boc**) was added DIPEA (6.6 mL, 40 mmol, 1.5 eq.) in one portion at 0 °C under N_2. The mixture was stirred at rt for 6 h. TLC (PE/EA = 2/1) showed the

reaction was complete. The reaction mixture was concentrated under reduced pressure to remove DCM. The residue was purified by flash column chromatography (SiO_2, PE/EA) to get compound **S-1-011**(9.7 g, 98% yield).
^{1}H NMR (400 MHz, Chloroform-*d*) δ 8.87 (m, 2H), 4.35 (q, *J*=7.1 Hz, 1H), 3.87–3.24 (m, 8H), 1.98 (m, 4H), 1.46 (s, 9H), 1.37 (t, *J* = 7.1 Hz, 3H).

Synthesis of **S-4**

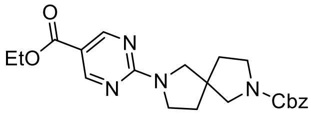

To a solution of compound **S-1-011**(9.5 g, 25.4 mmol) in DCM (51 mL, 0.5 M) was added HCl/1, 4-dioxane (4 M, 64 mL, 256 mmol, 10 eq.). The mixture was stirred at rt for 0.5 h. TLC (PE/EA = 2/1) showed the reaction was complete. The solvent was removed in vacuo. To the resultant mixture was added DCM (130 mL, 0.2 M), DIPEA (12.6 mL, 76.2 mmol, 3 eq.), then Cbz-Cl (4.4 mL, 30.8 mmol, 1.2 eq.) was added slowly to the mixture. The mixture was stirred at rt for 6 h. TLC (PE/EA = 2/1) showed the reaction was complete. The reaction mixture was poured into saturated $NaHCO_3$ and the aqueous phase was extracted three times with DCM (300 mL in total). The combined organic layers was washed once with brine, dried (Na_2SO_4), and the solvent was removed in vacuo. The residue was purified by flash column chromatography (SiO_2, PE/EA) to get compound **S-4** (10 g, 96% yield).
^{1}H NMR (400 MHz, Chloroform-*d*) δ 8.86 (m, 2H), 7.35 (m, 5H), 5.14 (m, 2H), 4.34 (q, *J*=7.1 Hz, 2H), 3.83–3.62 (m, 3H), 3.57 (m, 3H), 3.50–3.36 (m, 2H), 2.11–1.87 (m, 4H), 1.37 (t, *J*=7.1 Hz, 3H).

Synthesis of **S-5**

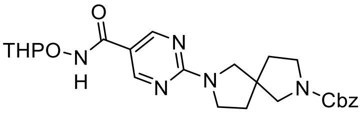

To a mixture of **S-4** (10 g, 24.5 mmol), and potassium carbonate (8.5 g, 61.5 mmol, 2.5 eq.) was added H_2O (60 mL) and MeOH (60 mL). The mixture was heated to 65 °C for 18 h. TLC (PE/EA = 2/1) showed the reaction was complete. The reaction mixture was concentrated under reduced pressure to remove solvent. The mixture was then acidified to pH = 2~3 with 2 M HCl, causing a solid to precipitate. This solid was collected, washed with large amount of H_2O to pH = ca.7 and dried in vacuo. To the dried mixture was added HOBt (9.9 g, 73.5 mmol, 3 eq.), DCM (120 mL, 0.2 M) and stirred at rt under a nitrogen atmosphere until all the solid dissolved. EDCI (14 g, 73.5 mmol, 3 eq.) was added and stirred for 30 min. $THPONH_2$ (5.7 g, 49 mmol, 2 eq.) and TEA (6.8 mL, 49 mmol, 2 eq.) and the mixture was allowed to stirred at rt for 72 h. TLC (DCM/MeOH = 9/1) showed the reaction was complete. The mixture was then diluted with H_2O and extracted three times with DCM. The combined organic layers were wash twice with H_2O, once with brine, dried (Na_2SO_4), and the solvent was removed in vacuo. The residue was purified by column chromatography (SiO_2, DCM/MeOH) to get compound **S-5** (10.6 g, 90% yield).
^{1}H NMR (400 MHz, DMSO-d_6) δ 11.52 (s, 1H), 8.68 (m, 2H), 7.54–7.16 (m, 5H), 5.07 (m, 2H), 4.96 (m, 1H), 4.20–3.93 (m, 1H), 3.63 (m, 2H), 3.58–3.40 (m, 5H), 3.34 (m, 3H), 1.93 (m, 4H), 1.72 (m, 3H), 1.55 (m, 3H).

Synthesis of **S-6**

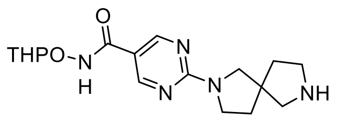

S-5 (0.48 g 1 mmol) was dissolved into DCM (5 mL) and MeOH (5 mL). To the solvent was added 10% Pd/C (0.19 g, wetted with ca. 55% water) and Et_3SiH (96 μL, 0.6 mmol). The mixture was allowed to stirred at rt for 1 h, and Et_3SiH (96 μL, 0.6 mmol) was added to the mixture. After 1 h, the mixture was filter through celite, and the solid residure was washed with MeOH. The solvent was rotavapored to dryness, and the residue was purified by column chromatography (SiO_2, DCM/MeOH) to get compound **S-6** (0.29 g, 84% yield).

^{1}H NMR (400 MHz, DMSO-d_6) δ 8.73 (s, 2), 5.01 (m, 1H), 4.10 (m, 1H), 3.75–3.43 (m, 6H), 3.09 (m, 2H), 3.00–2.83 (m, 2H), 2.02 (m, 2H), 1.82 (m, 5H), 1.61 (m, 3H).

Synthesis of **JX21101-JX21143**

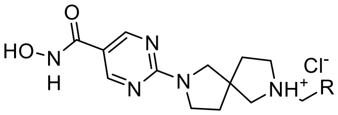

S-5 (0.48 g 1 mmol) was hydrogenated via Pd/C-Et_3SiH, and the crude residure was directly used without purification. To the crude mixture was add MeOH (10 mL), R-CHO (1.2 mmol), $NaBH_3(CN)$ (5 mmol) and stirred for 24 h. The reaction mixture was poured into saturated $NaHCO_3$ and the aqueous phase was extracted three times with DCM (75 mL in total). The combined organic layers was washed once with brine, dried (Na_2SO_4), and the solvent was removed in vacuo. The residue was purified by flash column chromatography (SiO_2, DCM/MeOH) and the product was dissolved into DCM (0.05 M) under a nitrogen atmosphere and HCl/1, 4-dioxane (4 M, 2.4 eq.) was add in one portion. The mixture was stirred at rt for 30 min and TLC showed the reaction was complete. The reaction mixture was filtered and the resulted precipitate was wash with large amount of DCM and ether, dried in vacuo to get compound. Similarily treated with HCl/1, 4-dioxane solution in DCM, **JX21131** was synthesized from **S-5**.

***N*-Hydroxy-2-(7-(naphthalen-2-ylmethyl)-2,7-diazaspiro[4.4]nonan-2-yl)pyrimidine-5-carboxamide hydrochloride (JX21101)**

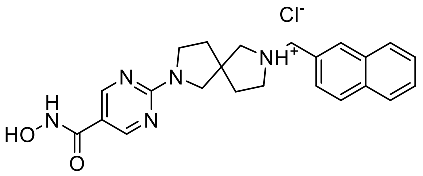

Compound **JX21101** was synthesized according to the above general synthetic method.

190 mg (43% yield from **S-5**); ^{1}H NMR (400 MHz, DMSO-d_6) δ 12.02–11.82 (m, 1H), 8.86–8.66 (m, 2H), 8.17 (s, 1H), 8.09–7.83 (m, 4H), 7.59 (m, 2H), 4.58 (m, 2H), 3.76 – 3.50 (m, 5H), 3.39 (m, 2H), 3.33–3.25 (m, 1H), 2.34–1.92 (m, 4H); HRMS (ESI) m/z calcd. for $C_{23}H_{26}N_5O_2$ $[M+H]^+$ 404.2087, found 404.2084; Purity: 96% (254 nm, RT = 4.007 min).

2-(7-((6-Chloro-1-methyl-1*H*-indol-3-yl)methyl)-2,7-diazaspiro[4.4]nonan-2-yl)-*N*-hydroxypyrimidine-5-carboxamide hydrochloride (JX21102)

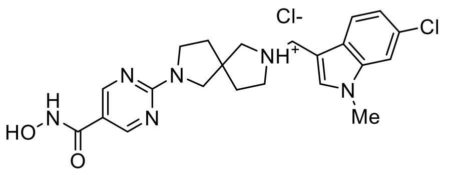

Compound **JX21102** was synthesized according to the above general synthetic method.
169 mg (35% yield from **S-5**); ^{1}H NMR (400 MHz, DMSO-d_6) δ 11.47–11.08 (m, 1H), 8.69 (m, 2H), 7.90 (d, J=8.5 Hz, 1H), 7.69 (s, 1H), 7.63 (s, 1H), 7.15 (d, J=7.7 Hz, 1H), 4.50 (m, 2H), 3.81 (s, 3H), 3.57 (m, 5H), 3.42 (m, 1H), 3.31 (m, 1H), 3.21 (m, 1H), 2.06 (m, 4H); HRMS (ESI) m/z calcd. for $C_{22}H_{26}ClN_6O_2$ [M+H]$^+$ 441.1806, found 441.1807; Purity: 97% (254 nm, RT = 4.095 min).

***N*-Hydroxy-2-(7-((5-methoxy-1-methyl-1*H*-indol-3-yl)methyl)-2,7-diazaspiro[4.4]nonan-2-yl)pyrimidine-5-carboxamide hydrochloride (JX21104)**

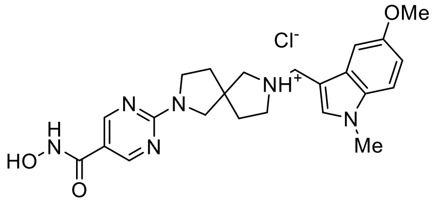

Compound **JX21104** was synthesized according to the above general synthetic method.
192 mg (41% yield from **S-5**); ^{1}H NMR (400 MHz, DMSO-d_6) δ 11.36–10.93 (m, 2H), 8.68 (m, 2H), 7.57 (s, 1H), 7.43 (s, 1H), 7.37 (d, J=8.9 Hz, 1H), 6.89–6.81 (m, 1H), 4.49 (m, 2H), 3.81 (d, 3H), 3.78 (s, 3H), 3.64 (m, 1H), 3.60–3.47 (m, 4H), 3.42 (m, 1H), 3.32 (m, 1H), 3.27–3.16 (m, 1H), 2.05 (m, 4H); HRMS (ESI) m/z calcd. for $C_{23}H_{29}N_6O_3$ [M+H]$^+$ 437.2301, found 437.2300; Purity: 99% (254 nm, RT = 4.064 min).

2-(7-Benzyl-2,7-diazaspiro[4.4]nonan-2-yl)-*N*-hydroxypyrimidine-5-carboxamide hydrochloride (JX21105)

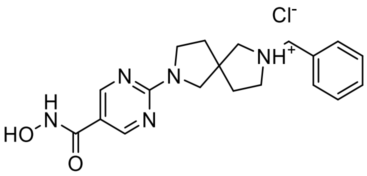

Compound **JX21105** was synthesized according to the above general synthetic method.
192 mg (49% yield from **S-5**); ^{1}H NMR (400 MHz, DMSO-d_6) δ 11.52–11.28 (m, 1H), 8.68 (m, 2H), 7.65 (m, 2H), 7.44 (m, 3H), 4.48–4.31 (m, 2H), 3.73–3.54 (m, 4H), 3.49 (m, 1H), 3.34 (m, 2H), 3.25–3.14 (m, 1H), 2.07 (m, 4H); HRMS (ESI) m/z calcd. for $C_{19}H_{24}N_5O_2$ [M+H]$^+$ 354.1930, found 354.1932; Purity: 96% (254 nm, RT = 3.954 min).

2-(7-((6-Fluoro-1-methyl-1*H*-indol-3-yl)methyl)-2,7-diazaspiro[4.4]nonan-2-yl)-*N*-hydroxypyrimidine-5-carboxamide hydrochloride (JX21106)

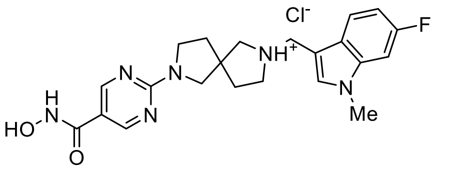

Compound **JX21106** was synthesized according to the above general synthetic method.

171 mg (37% yield from **S-5**); ^{1}H NMR (400 MHz, DMSO-d_6) δ 11.41 (m, 1H), 8.71 (d, 2H), 7.91 (dd, J=8.8, 5.3 Hz, 1H), 7.67 (s, 1H), 7.37 (d, J=9.8 Hz, 1H), 6.99 (q, J=8.0 Hz, 1H), 4.51 (m, 2H), 3.79 (s, 3H), 3.69–3.49 (m, 5H), 3.43 (m, 1H), 3.32 (m, 1H), 3.22 (m, 1H), 2.05 (m, 4H); HRMS (ESI) m/z calcd. for $C_{22}H_{26}FN_6O_2$ [M+H]$^+$ 425.2101, found 425.2102; Purity: 96% (254 nm, RT = 4.074 min).

2-(7-((5-Cyano-1-methyl-1*H*-indol-3-yl)methyl)-2,7-diazaspiro[4.4]nonan-2-yl)-*N*-hydroxypyrimidine-5-carboxamide hydrochloride (JX21108)

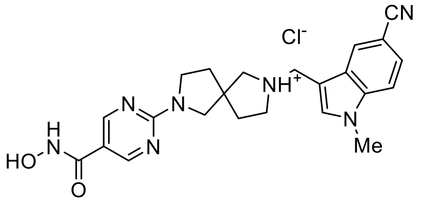

Compound **JX21108** was synthesized according to the above general synthetic method.

205 mg (44% yield from **S-5**); ^{1}H NMR (400 MHz, DMSO-d_6) δ 11.41–11.13 (m, 2H), 8.69 (m, 2H), 8.52 (s, 1H), 7.87 (s, 1H), 7.70 (d, J=8.5 Hz, 1H), 7.58 (d, J=8.5 Hz, 1H), 4.57 (m, 2H), 3.88 (s, 3H), 3.65 (m, 1H), 3.55 (m, 4H), 3.43 (m, 1H), 3.34 (m, 1H), 3.28–3.18 (m, 1H), 2.19–1.93 (m, 4H); ^{13}C NMR (151 MHz, DMSO) δ 161.94, 161.88, 159.83, 159.80, 157.07, 156.77, 137.97, 134.92, 134.88, 127.14, 127.11, 125.18, 125.15, 124.36, 124.33, 120.53, 120.51, 114.40, 111.60, 104.75, 104.73, 101.75, 59.71, 59.19, 56.58, 56.23, 52.06, 52.03, 47.64, 47.49, 47.18, 47.14, 45.76, 45.71, 35.56, 35.26, 33.86, 33.47, 33.05; HRMS (ESI) m/z calcd. for $C_{23}H_{26}N_7O_2$ [M+H]$^+$ 432.2148, found 432.2149; Purity: 99% (254 nm, RT = 4.013 min).

2-(7-((2,3-Dihydrobenzofuran-5-yl)methyl)-2,7-diazaspiro[4.4]nonan-2-yl)-*N*-hydroxypyrimidine-5-carboxamide hydrochloride (JX21109)

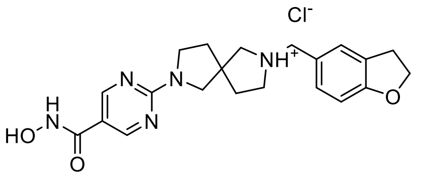

Compound **JX21109** was synthesized according to the above general synthetic method.

138 mg (32% yield from **S-5**); ^{1}H NMR (400 MHz, DMSO-d_6) δ 11.53–11.32 (m, 1H), 8.70 (m, 2H), 7.51 (s, 1H), 7.42–7.30 (m, 1H), 6.80 (d, J=8.1 Hz, 1H), 4.55 (t, J=8.7 Hz, 1H), 4.27 (m, 2H), 3.74–3.52 (m, 4H), 3.45 (m, 1H), 3.40–3.23 (m, 2H), 3.22–3.10 (m, 3H), 2.07 (m, 4H); HRMS (ESI) m/z calcd. for $C_{21}H_{26}N_5O_3$ [M+H]$^+$ 396.2036, found 396.2035; Purity: 97% (254 nm, RT = 3.986 min).

N-Hydroxy-2-(7-(thiophen-2-ylmethyl)-2,7-diazaspiro[4.4]nonan-2-yl)pyrimidine-5-carboxamide hydrochloride (JX21110)

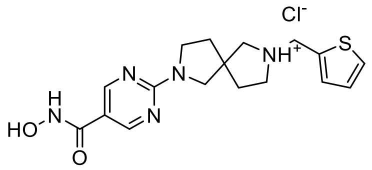

Compound **JX21110** was synthesized according to the above general synthetic method.

130 mg (33% yield from **S-5**); ^{1}H NMR (400 MHz, DMSO-d_6) δ 11.65 (d, J=41.7 Hz, 1H), 8.70 (m, 2H), 7.67 (d, J=5.1 Hz, 1H), 7.44 (s, 1H), 7.12 (t, J=4.4 Hz, 1H), 4.62 (m, 2H), 3.70–3.46 (m, 5H) 3.41 (m, 1H), 3.31 (m, 1H), 3.22 (m, 1H), 2.23–1.92 (m, 4H); HRMS (ESI) m/z calcd. for $C_{17}H_{22}N_5O_2S$ $[M+H]^+$ 360.1494, found 360.1495; Purity: 98% (254 nm, RT = 4.009 min).

2-(7-(Furan-2-ylmethyl)-2,7-diazaspiro[4.4]nonan-2-yl)-N-hydroxypyrimidine-5-carboxamide hydrochloride (JX21111)

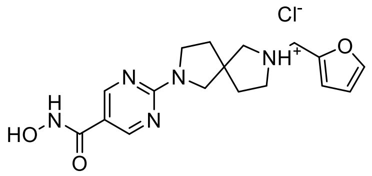

Compound **JX21111** was synthesized according to the above general synthetic method.

128 mg (34% yield from **S-5**); ^{1}H NMR (400 MHz, DMSO-d_6) δ 11.52 (m, 1H), 8.70 (m, 2H), 7.80 (d, J=2.3 Hz, 1H), 6.74 (d, J=3.3 Hz, 1H), 6.61–6.52 (m, 1H), 4.46 (m, 2H), 3.69–3.40 (m, 6H), 3.30 (m, 1H), 3.25–3.16 (m, 1H), 2.20–1.92 (m, 4H); HRMS (ESI) m/z calcd. for $C_{17}H_{22}N_5O_3$ $[M+H]^+$ 344.1723, found 344.1724; Purity: 95% (254 nm, RT = 3.972 min).

2-(7-(Cyclohexylmethyl)-2,7-diazaspiro[4.4]nonan-2-yl)-N-hydroxypyrimidine-5-carboxamide hydrochloride (JX21112)

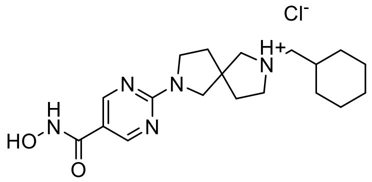

Compound **JX21112** was synthesized according to the above general synthetic method.

160 mg (40% yield from **S-5**); ^{1}H NMR (400 MHz, DMSO-d_6) δ 10.80 (d, 1H), 8.70 (s, 2H), 3.81–3.51 (m, 5H), 3.29–2.94 (m, 3H), 2.06 (m, 3H), 1.89 (m, 1H), 1.81 (m, 1H), 1.76–1.56 (m, 3H), 1.22 (m, 4H), 0.94 (m, 2H); HRMS (ESI) m/z calcd. for $C_{19}H_{30}N_5O_2$ $[M+H]^+$ 360.2400, found 360.2401; Purity: 96% (254 nm, RT = 3.996 min).

2-(7-(Benzofuran-3-ylmethyl)-2,7-diazaspiro[4.4]nonan-2-yl)-*N*-hydroxypyrimidine-5-carboxamide hydrochloride (JX21113)

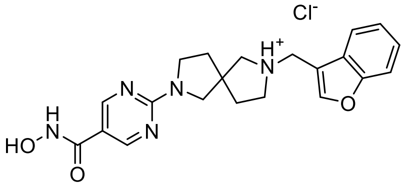

Compound **JX21113** was synthesized according to the above general synthetic method.

202 mg (47% yield from **S-5**); ^{1}H NMR (400 MHz, DMSO-d_6) δ 11.93–11.59 (m, 1H), 8.71 (m, 2H), 8.32 (s, 1H), 8.03 (d, J = 7.5 Hz, 1H), 7.65 (d, J = 7.9 Hz, 1H), 7.37 (m, 2H), 4.58 (m, 2H), 3.80–3.46 (m, 6H), 3.39 (m, 1H), 3.32–3.20 (m, 1H), 2.31–1.89 (m, 4H). HRMS (ESI) m/z calcd. for $C_{21}H_{24}N_5O_3$ $[M+H]^+$ 394.1879, found 394.1880; Purity: 99% (254 nm, RT = 3.994 min);

***N*-Hydroxy-2-(7-(quinolin-4-ylmethyl)-2,7-diazaspiro[4.4]nonan-2-yl)pyrimidine-5-carboxamide hydrochloride (JX21114)**

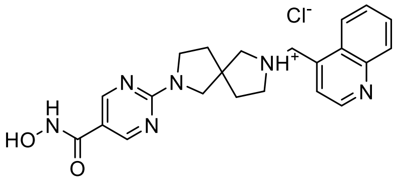

Compound **JX21114** was synthesized according to the above general synthetic method.

185 mg (42% yield from **S-5**); ^{1}H NMR (400 MHz, DMSO-d_6) δ 12.44–12.10 (m, 1H), 9.33 (m, 1H), 8.68 (m, 3H), 8.60–8.38 (m, 2H), 8.13 (t, J=7.5 Hz, 1H), 7.98 (t, J=7.8 Hz, 1H), 5.26 (m, 2H), 3.80–3.44 (m, 7H), 3.43–3.32 (m, 1H), 2.32–1.91 (m, 4H); HRMS (ESI) m/z calcd. for $C_{22}H_{25}N_6O_2$ $[M+H]^+$ 405.2039, found 405.2040; Purity: 95% (254 nm, RT = 4.099 min).

2-(7-(Benzo[*b*]thiophen-3-ylmethyl)-2,7-diazaspiro[4.4]nonan-2-yl)-*N*-hydroxypyrimidine-5-carboxamide hydrochloride (JX21115)

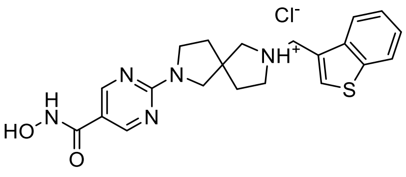

Compound **JX21115** was synthesized according to the above general synthetic method.

189 mg (42 yield from **S-5**); ^{1}H NMR (400 MHz, DMSO-d_6) δ 11.50 (m, 1H), 8.70 (m, 2H), 8.26 (d, J=5.3 Hz, 1H), 8.18 (d, J=7.9 Hz, 1H), 8.07 (d, J=7.8 Hz, 1H), 7.47 (m, 2H), 4.71 (m, 2H), 3.76–3.46 (m, 6H), 3.40 (m, 1H), 3.33–3.19 (m, 1H), 2.23–1.94 (m, 4H); HRMS (ESI) m/z calcd. for $C_{21}H_{24}N_5O_2S$ $[M+H]^+$ 410.1651, found 410.1652; Purity: 97% (254 nm, RT = 4.112 min).

2-(7-([1,1'-Biphenyl]-4-ylmethyl)-2,7-diazaspiro[4.4]nonan-2-yl)-*N*-hydroxypyrimidine-5-carboxamide hydrochloride (JX21116)

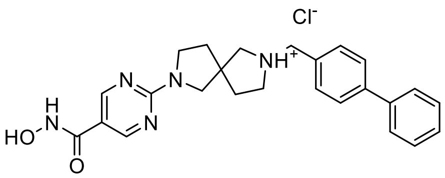

Compound **JX21116** was synthesized according to the above general synthetic method.

202 mg (43% yield from **S-5**); ^{1}H NMR (400 MHz, DMSO-d_6) δ 11.70 (m, 1H), 8.70 (m, 2H), 7.84–7.66 (m, 6H), 7.49 (t, 2H), 7.39 (t, *J*=7.3 Hz, 1H), 4.53–4.34 (m, 2H), 3.74–3.50 (m, 5H), 3.47–3.29 (m, 2H), 3.29–3.17 (m, 1H), 2.11 (m, 4H); HRMS (ESI) m/z calcd. for $C_{25}H_{28}N_5O_2$ $[M+H]^+$ 430.2243, found 430.2244; Purity: 99% (254 nm, RT = 3.970 min).

2-(7-(Benzofuran-2-ylmethyl)-2,7-diazaspiro[4.4]nonan-2-yl)-*N*-hydroxypyrimidine-5-carboxamide hydrochloride (JX21117)

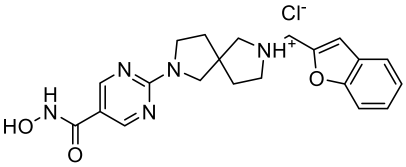

Compound **JX21117** was synthesized according to the above general synthetic method.

174 mg (40% yield from **S-5**); ^{1}H NMR (400 MHz, DMSO-d_6) δ 11.87 (m, 1H), 8.70 (m, 2H), 7.72 (d, *J*=7.7 Hz, 1H), 7.62 (d, *J*=8.2 Hz, 1H), 7.39 (t, *J*=7.7 Hz, 1H), 7.30 (t, *J*=7.5 Hz, 1H), 7.23 (s, 1H), 4.77–4.60 (m, 2H), 3.61 (m, 6H), 3.42 (m, 1H), 3.32 (m, 1H), 2.09 (m, 4H); HRMS (ESI) m/z calcd. for $C_{21}H_{24}N_5O_3$ $[M+H]^+$ 394.1879, found 394.1880; Purity: 97% (254 nm, RT = 4.060 min).

2-(7-(Benzo[b]thiophen-2-ylmethyl)-2,7-diazaspiro[4.4]nonan-2-yl)-*N*-hydroxypyrimidine-5-carboxamide hydrochloride (JX21118)

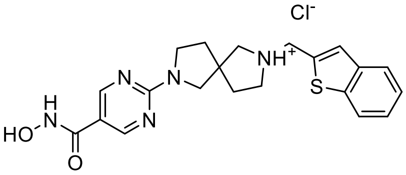

Compound **JX21118** was synthesized according to the above general synthetic method.

184 mg (41% yield from **S-5**); ^{1}H NMR (400 MHz, DMSO-d_6) δ 12.09–11.84 (m, 1H), 8.70 (m, 2H), 8.09–8.00 (m, 1H), 7.90 (m, 1H), 7.75 (s, 1H), 7.52–7.31 (m, 2H), 4.82–4.63 (m, 2H), 3.71–3.55 (m, 5H), 3.49 (m, 1H), 3.38 (m, 1H), 3.28 (m, 1H), 2.10 (m, 4H); HRMS (ESI) m/z calcd. for $C_{21}H_{24}N_5O_2S$ $[M+H]^+$ 410.1651, found 410.1652; Purity: 96% (254 nm, RT = 4.009 min).

N-Hydroxy-2-(7-(pyridin-3-ylmethyl)-2,7-diazaspiro[4.4]nonan-2-yl)pyrimidine-5-carboxamide hydrochloride (JX21119)

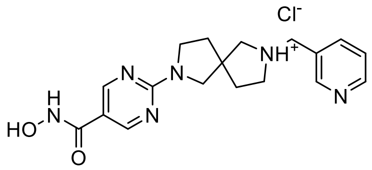

Compound **JX21119** was synthesized according to the above general synthetic method.
176 mg (45% yield from **S-5**); ^{1}H NMR (400 MHz, DMSO-d_6) δ 12.23 (m, 1H), 9.15 (s, 1H), 8.93 (d, J=5.5 Hz, 1H), 8.78 (m, 1H), 8.69 (m, 2H), 8.02 (t, J=6.9 Hz, 1H), 4.63 (m, 2H), 3.77–3.52 (m, 5H), 3.49–3.19 (m, 3H), 2.09 (m, 4H); HRMS (ESI) m/z calcd. for $C_{18}H_{23}N_6O_2$ $[M+H]^+$ 355.1882, found 355.1885; Purity: 97% (254 nm, RT = 4.072 min).

N-Hydroxy-2-(7-(thiophen-3-ylmethyl)-2,7-diazaspiro[4.4]nonan-2-yl)pyrimidine-5-carboxamide hydrochloride (JX21120)

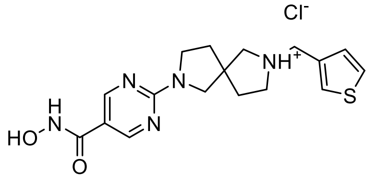

Compound **JX21120** was synthesized according to the above general synthetic method.
146 mg (37% yield from **S-5**); ^{1}H NMR (400 MHz, DMSO-d_6) δ 11.76 (m, 1H), 8.71 (m, 2H), 7.82 (d, J=3.0 Hz, 1H), 7.63 (dd, J=5.0, 3.0 Hz, 0H), 7.44 (t, J=5.0 Hz, 1H), 4.45–4.32 (m, 2H), 3.70–3.54 (m, 4H), 3.47 (m, 1H), 3.36 (m, 1H), 3.27 (m, 1H), 3.18 (m, 1H), 2.23–1.93 (m, 4H); ^{1}HRMS (ESI) m/z calcd. for $C_{17}H_{22}N_5O_2S$ $[M+H]^+$ 360.1494, found 360.1495; Purity: 95% (254 nm, RT = 3.996 min).

2-(7-(Cyclopentylmethyl)-2,7-diazaspiro[4.4]nonan-2-yl)-N-hydroxypyrimidine-5-carboxamide hydrochloride (JX21121)

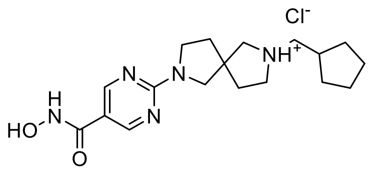

Compound **JX21121** was synthesized according to the above general synthetic method.
133 mg (35% yield from **S-5**); ^{1}H NMR (400 MHz, DMSO-d_6) δ 10.94 (m, 1H), 8.71 (s, 2H), 3.89–3.52 (m, 6H), 3.29–3.03 (m, 4H), 2.33–1.96 (m, 5H), 1.83 (m, 2H), 1.69–1.48 (m, 4H), 1.26 (m, 3H); HRMS (ESI) m/z calcd. for $C_{18}H_{28}N_5O_2$ $[M+H]^+$ 346.2243, found 346.2244; Purity: 96% (254 nm, RT = 4.046 min).

N-Hydroxy-2-(7-(imidazo[1,2-a]pyridin-3-ylmethyl)-2,7-diazaspiro[4.4]nonan-2-yl)pyrimidine-5-carboxamide hydrochloride (JX21122)

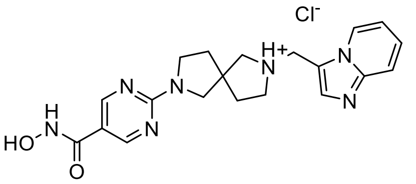

Compound **JX21122** was synthesized according to the above general synthetic method.

128 mg (30% yield from **S-5**); ^{1}H NMR (400 MHz, DMSO-d_6) δ 12.11 (d, 1H), 11.13 (s, 1H), 9.33 (d, J=6.8 Hz, 1H), 8.69 (s, 2H), 8.49 (s, 1H), 8.01 (m, 2H), 7.58 (td, J=6.8, 2.1 Hz, 2H), 5.00 (m, 2H), 3.89–3.32 (m, 8H), 2.10 (m, 4H); HRMS (ESI) m/z calcd. for $C_{20}H_{24}N_7O_2$ [M+H]$^+$ 394.1991, found 394.1992; Purity: 99% (254 nm, RT = 4.009 min).

N-Hydroxy-2-(7-((1-methyl-1H-indol-2-yl)methyl)-2,7-diazaspiro[4.4]nonan-2-yl)pyrimidine-5-carboxamide hydrochloride (JX21123)

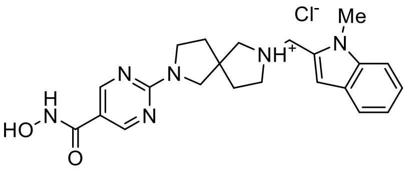

Compound **JX21123** was synthesized according to the above general synthetic method.

131 mg (30% yield from **S-5**); ^{1}H NMR (400 MHz, DMSO-d_6) δ 11.58 (m, 1H), 8.69 (m, 2H), 7.58 (d, J=7.9 Hz, 1H), 7.50 (d, J=8.3 Hz, 1H), 7.21 (t, J=7.7 Hz, 1H), 7.07 (t, J=7.4 Hz, 1H), 6.84 (s, 1H), 4.76–4.60 (m, 2H), 3.88 (s, 3H), 3.75–3.47 (m, 6H), 3.40 (m, 1H), 3.34 (m, 1H), 2.26–1.97 (m, 4H); HRMS (ESI) m/z calcd. for $C_{22}H_{27}N_6O_2$ [M+H]$^+$ 407.2195, found 407.2194; Purity: 97% (254 nm, RT = 4.131 min).

N-Hydroxy-2-(7-((1-methyl-1H-indazol-3-yl)methyl)-2,7-diazaspiro[4.4]nonan-2-yl)pyrimidine-5-carboxamide hydrochloride (JX21124)

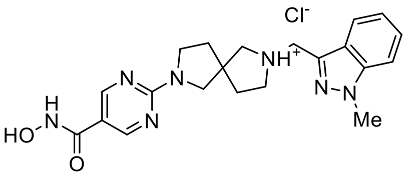

Compound **JX21124** was synthesized according to the above general synthetic method.

142 mg (32% yield from **S-5**); ^{1}H NMR (400 MHz, DMSO-d_6) δ 11.21 (m, 2H), 8.68 (m, 2H), 8.05 (d, J=8.2 Hz, 1H), 7.71 (d, J=8.5 Hz, 1H), 7.54–7.33 (m, 1H), 7.25 (td, J=7.5, 4.6 Hz, 1H), 4.78 (m, 2H), 4.10 (s, 3H), 3.73–3.48 (m, 6H), 3.44 (m, 1H), 3.33 (m, 1H), 2.25–1.89 (m, 4H); HRMS (ESI) m/z calcd. for $C_{21}H_{26}N_7O_2$ [M+H]$^+$ 408.2148, found 408.2149; Purity: 98% (254 nm, RT = 3.915 min).

***N*-Hydroxy-2-(7-(naphthalen-1-ylmethyl)-2,7-diazaspiro[4.4]nonan-2-yl)pyrimidine-5-carboxamide hydrochloride (JX21125)**

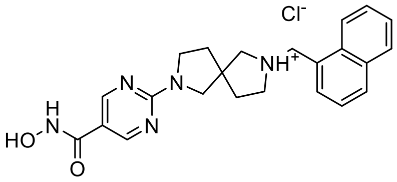

Compound **JX21125** was synthesized according to the above general synthetic method.

146 mg (33% yield from **S-5**); ^{1}H NMR (400 MHz, DMSO-d_6) δ 11.01–10.76 (m, 1H), 8.74–8.63 (m, 2H), 8.39 (d, *J*=8.4 Hz, 1H), 8.04 (t, *J*=8.2 Hz, 2H), 7.93 (t, *J*=7.3 Hz, 1H), 7.71–7.57 (m, 3H), 4.92 (m, 2H), 3.77–3.44 (m, 7H), 3.37 (m, 1H), 2.14 (m, 2H), 2.01 (m, 1H); HRMS (ESI) m/z calcd. for $C_{23}H_{26}N_5O_2$ $[M+H]^+$ 404.2087, found 404.2088; Purity: 100% (254 nm, RT = 4.015 min).

***N*-Hydroxy-2-(7-((1-methyl-1*H*-pyrrolo[2,3-c]pyridin-3-yl)methyl)-2,7-diazaspiro[4.4]nonan-2-yl)pyrimidine-5-carboxamide hydrochloride (JX21129)**

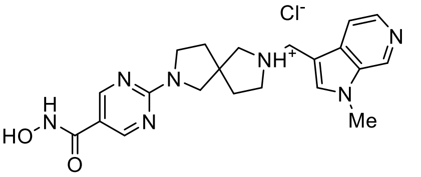

Compound **JX21129** was synthesized according to the above general synthetic method.

122 mg (27% yield from **S-5**); ^{1}H NMR (400 MHz, DMSO-d_6) δ 11.43 (m, 1H), 8.70 (m, 2H), 8.47 (d, *J*=7.9 Hz, 1H), 8.37 (d, *J*=4.8 Hz, 1H), 7.88 (s, 1H), 7.27 (dt, *J*=7.9, 4.8 Hz, 1H), 4.55 (m, 2H), 3.69–3.50 (m, 5H), 3.44 (m, 1H), 3.37–3.29 (m, 1H), 3.23 (m, 1H), 2.20–1.92 (m, 4H); HRMS (ESI) m/z calcd. for $C_{21}H_{26}N_7O_2$ $[M+H]^+$ 408.2148, found 408.2149; Purity: 99% (254 nm, RT = 4.002 min).

***N*-Hydroxy-2-(7-((1-methyl-1*H*-pyrrolo[2,3-b]pyridin-3-yl)methyl)-2,7-diazaspiro[4.4]nonan-2-yl)pyrimidine-5-carboxamide hydrochloride (JX21130)**

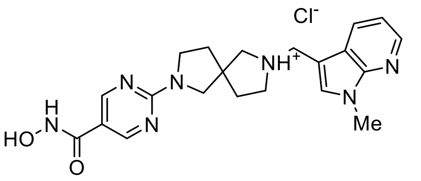

Compound **JX21130** was synthesized according to the above general synthetic method.

126 mg (28% yield from **S-5**); ^{1}H NMR (400 MHz, DMSO-d_6) δ 11.22 (m, 2H), 8.68 (m, 2H), 8.41 (d, *J*=7.9 Hz, 1H), 8.35 (d, *J*=4.7 Hz, 1H), 7.86 (s, 1H), 7.24 (dt, *J*=7.9, 4.7 Hz, 1H), 4.54 (m, 2H), 3.87 (s, 3H), 3.69–3.50 (m, 5H), 3.43 (m, 1H), 3.33 (m, 1H), 3.22 (m, 1H), 2.06 (m, 4H); HRMS (ESI) m/z calcd. for $C_{21}H_{26}N_7O_2$ $[M+H]^+$ 408.2148, found 408.2149; Purity: 97% (254 nm, RT = 4.080 min).

N-Hydroxy-2-(2,7-diazaspiro[4.4]nonan-2-yl)pyrimidine-5-carboxamide hydrochloride (JX21131)

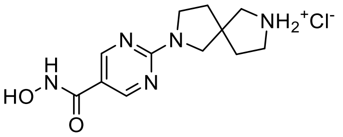

Compound **JX21131** was synthesized according to the above general synthetic method.

126 mg (42% yield from **S-5**); ^{1}H NMR (400 MHz, DMSO-d_6) δ 9.49 (m, 2H), 8.70 (s, 2H), 3.72–3.51 (m, 4H), 3.28 (m, 2H), 3.16 (m, 2H), 2.21–1.89 (m, 4H); HRMS (ESI) m/z calcd. for $C_{12}H_{18}N_5O_2$ [M+H]$^+$ 264.1460, found 264.1461; Purity: 100% (254 nm, RT = 4.044 min).

N-Hydroxy-2-(7-(quinolin-6-ylmethyl)-2,7-diazaspiro[4.4]nonan-2-yl)pyrimidine-5-carboxamide hydrochloride (JX21137)

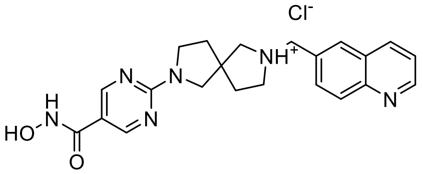

Compound **JX21137** was synthesized according to the above general synthetic method.

137 mg (31% yield from **S-5**); ^{1}H NMR (400 MHz, DMSO-d_6) δ 12.06 (m, 1H), 9.28 (m, 1H), 8.99 (m, 1H), 8.68 (m, 2H), 8.53 (m, 1H), 8.45–8.35 (m, 2H), 8.03 (m, 1H), 4.83–4.60 (m, 2H), 3.79–3.53 (m, 5H), 3.41 (m, 5.8 Hz, 2H), 3.29 (m, 1H), 2.09 (m, 4H); HRMS (ESI) m/z calcd. for $C_{22}H_{25}N_6O_2$ [M+H]$^+$ 405.2039, found 405.2040; Purity: 96% (254 nm, RT = 3.966 min).

2-(7-(Anthracen-9-ylmethyl)-2,7-diazaspiro[4.4]nonan-2-yl)-N-hydroxypyrimidine-5-carboxamide hydrochloride (JX21138)

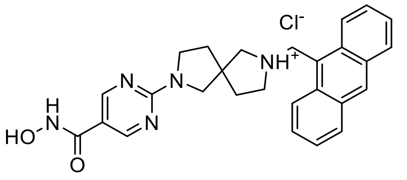

Compound **JX21138** was synthesized according to the above general synthetic method.

125 mg (26% yield from **S-5**); ^{1}H NMR (400 MHz, DMSO-d_6) δ 10.38 (m, 1H), 8.84 (s, 1H), 8.69 (m, 4H), 8.21 (d, J=8.4 Hz, 1H), 7.72 (q, J=6.9 Hz, 2H), 7.64 (m, 2H), 5.63–5.51 (m, 2H), 3.87–3.29 (m, 9H), 2.24–1.86 (m, 4H); HRMS (ESI) m/z calcd. for $C_{27}H_{28}N_5O_2$ [M+H]$^+$ 454.2243, found 454.2244; Purity: 98% (254 nm, RT = 3.966 min).

2-(7-(Benzo[d][1,3]dioxol-5-ylmethyl)-2,7-diazaspiro[4.4]nonan-2-yl)-N-hydroxypyrimidine-5-carboxamide hydrochloride (JX21140)

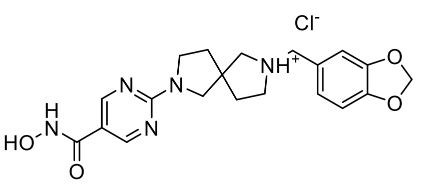

Compound **JX21140** was synthesized according to the above general synthetic method.

152 mg (35% yield from **S-5**); ^{1}H NMR (400 MHz, DMSO-d_6) δ 11.78–11.46 (m, 1H), 8.90–8.48 (m, 2H), 7.33 (d, *J*=5.6 Hz, 1H), 7.09 (d, *J*=8.0 Hz, 1H), 6.96 (d, *J*=7.7 Hz, 1H), 6.06 (s, 2H), 4.39–4.18 (m, 2H), 3.70–3.53 (m, 4H), 3.51–3.42 (m, 1H), 3.39–3.09 (m, 3H), 2.07 (m, 4H); HRMS (ESI) m/z calcd. for $C_{20}H_{24}N_5O_4$ $[M+H]^+$ 398.1828, found 398.1829; Purity: 100% (254 nm, RT = 4.051 min).

2-(7-((5-Bromo-1-methyl-1*H*-indol-3-yl)methyl)-2,7-diazaspiro[4.4]nonan-2-yl)-*N*-hydroxypyrimidine-5-carboxamide hydrochloride (JX21141)

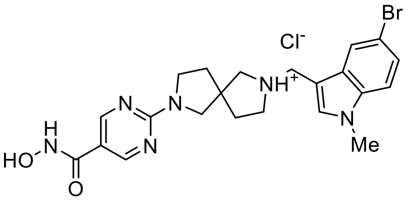

Compound **JX21141** was synthesized according to the above general synthetic method.
161 mg (31% yield from **S-5**); ^{1}H NMR (400 MHz, DMSO-d_6) δ 10.96 (m, 1H), 8.81–8.58 (m, 2H), 8.13 (*J*=1.9 Hz, 1H), 7.71 (s, 1H), 7.48 (d, *J*=8.7 Hz, 1H), 7.33 (dt, *J*=8.7, 1.9 Hz, 1H), 4.51 (m, 2H), 3.82 (s, 3H), 3.69–3.37 (m, 7H), 3.39–3.29 (m, 1H), 3.26–3.14 (m, 1H), 2.21–1.90 (m, 4H); HRMS (ESI) m/z calcd. for $C_{22}H_{26}BrN_6O_2$ $[M+H]^+$ 485.1301, found 485.1302; Purity: 99% (254 nm, RT = 4.094 min).

***N*-Hydroxy-2-(7-((6-methoxy-1-methyl-1*H*-indol-3-yl)methyl)-2,7-diazaspiro[4.4]nonan-2-yl)pyrimidine-5-carboxamide hydrochloride (JX21142)**

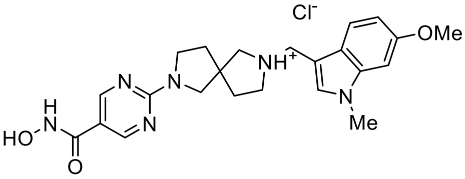

Compound **JX21142** was synthesized according to the above general synthetic method.
144 mg (30% yield from **S-5**); ^{1}H NMR (400 MHz, DMSO-d_6) δ 11.3—10.98 (m, 1H), 8.69 (m, 2H), 7.73 (dd, *J*=8.4, 2.0 Hz, 1H), 7.50 (s, 1H), 7.01 (d, *J*=2.2 Hz, 1H), 6.77 (td, *J*=8.4, 2.2 Hz, 1H), 4.47 (m, 2H), 3.81 (s, 3H), 3.77 (s, 3H), 3.67–3.47 (m, 5H), 3.41 (m, 1H), 3.30 (m, 1H), 3.21 (m, 1H), 2.20–1.91 (m, 4H); HRMS (ESI) m/z calcd. for $C_{23}H_{29}N_6O_3$ $[M+H]^+$ 437.2301, found 437.2302; Purity: 98% (254 nm, RT = 4.057 min).

***N*-Hydroxy-2-(7-(quinolin-2-ylmethyl)-2,7-diazaspiro[4.4]nonan-2-yl)pyrimidine-5-carboxamide hydrochloride (JX21143)**

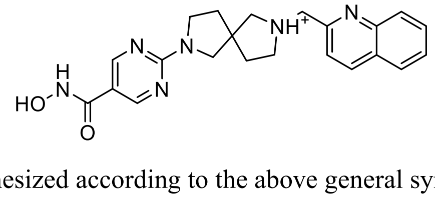

Compound **JX21143** was synthesized according to the above general synthetic method.
139 mg (32% yield from **S-5**); ^{1}H NMR (400 MHz, DMSO-d_6) δ 11.50 (s, 1H), 8.71 (s, 2H), 8.52 (d, *J* = 8.5 Hz, 1H), 8.08 (dd, 2H), 7.93—7.79 (m, 2H), 7.69 (t, *J* = 7.4 Hz, 1H), 4.83 (m, 2H), 3.79—3.39 (m, 10H), 2.14 (m, 4H);HRMS (ESI) m/z calcd. for $C_{22}H_{25}N_6O_2$ $[M+H]^+$ 405.2039, found 405.2040; Purity: 97% (254 nm, RT = 4.007 min).

3. Synthesis of enantiomeric JX21108

3.1 Synthetic route

Because of the failure to separate the chiral antipodes of **JX21108** via chiral column chromatography, both enantiomeric **JX21108** were prepared from enantiopure **S-1-011** using procedures in the synthesis of linker derivatives. **(+)-JX21108** was synthesized from **(+)-S-1-011**, and **(-)-JX21108** was synthesized from **(-)-S-1-011**. The enantiopurity of products was guaranteed by original substrate materials since no chiral manipulation was introduced during synthetic process.

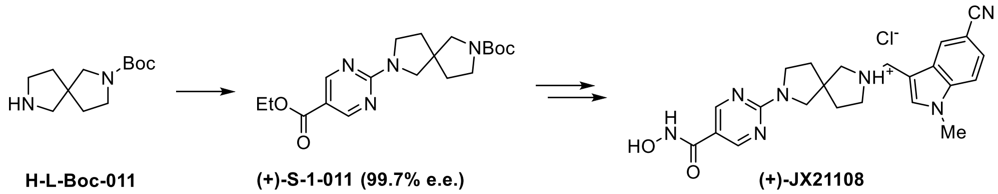

H-L-Boc-011

(+)-S-1-011 (99.7% e.e.)
(-)-S-1-011 (99.5% e.e.)
(chiral column separation)

(+)-JX21108
(-)-JX21108

Scheme 4. Synthesis of enantiomeric **JX21108**

3.2 Chiral column separation of **S-1-011**

3.2.1 Separation and analysis of **S-1-011**

Both 1.45 g **(+)-S-1-011** and **(-)-S-1-011** were obtain from 3.00 g racemic **S-1-011**.

(+)-S-1-011

$[\alpha]_{20}^{D}$ (deg mL^3 g^{-1} dm^{-1})=68 (c=0.01 g mL^{-3} in MeOH); ^{1}H NMR (400 MHz, Chloroform-*d*) δ 8.87 (m 2H), 4.35 (q, J = 7.1 Hz, 2H), 3.88—3.25 (m, 8H), 1.98 (m, 4H), 1.46 (s, 9H), 1.37 (t, J = 7.1 Hz, 3H).

(-)-S-1-011

$[\alpha]_{20}^{D}$ (deg mL^3 g^{-1} dm^{-1})=-66.3 (c=0.01 g mL^{-3} in MeOH); ^{1}H NMR (400 MHz, Chloroform-*d*) δ 8.87 (m, 1H), 4.35 (q, J = 7.1 Hz, 1H), 3.84 – 3.27 (m, 8H), 1.98 (m, 4H), 1.46 (s, 9H), 1.37 (t, J = 7.1 Hz, 3H).

The conditions of chiral separation and analysis were listed as followes:

Column :	CHIRALPAK ID
Column size	5.0 cm I.D. × 25 cm L, 10 µm
Sample solution	15 mg/ml in Mobile phase
Injection	15 ml
Mobile phase	EtOH/MeCN = 95/5(V/V)
Flow rate	60 ml/min
Wave length	UV 214 nm
Temperature	35 ℃

Separation conditions

Column	CHIRALPAK ID (ID00CD-TB002)
Column size	0.46 cm I.D. × 15 cm L
Injection	1 µl
Mobile phase	EtOH/MeCN = 95/5(V/V)
Flow rate	0.5 ml/min
Wave length	UV 254nm
Temperature	25 ℃
HPLC equipment	Shimadzu LC-20AD, CP-HPLC-05

Analyse conditions

3.2.2 Analyse result

S-01-011

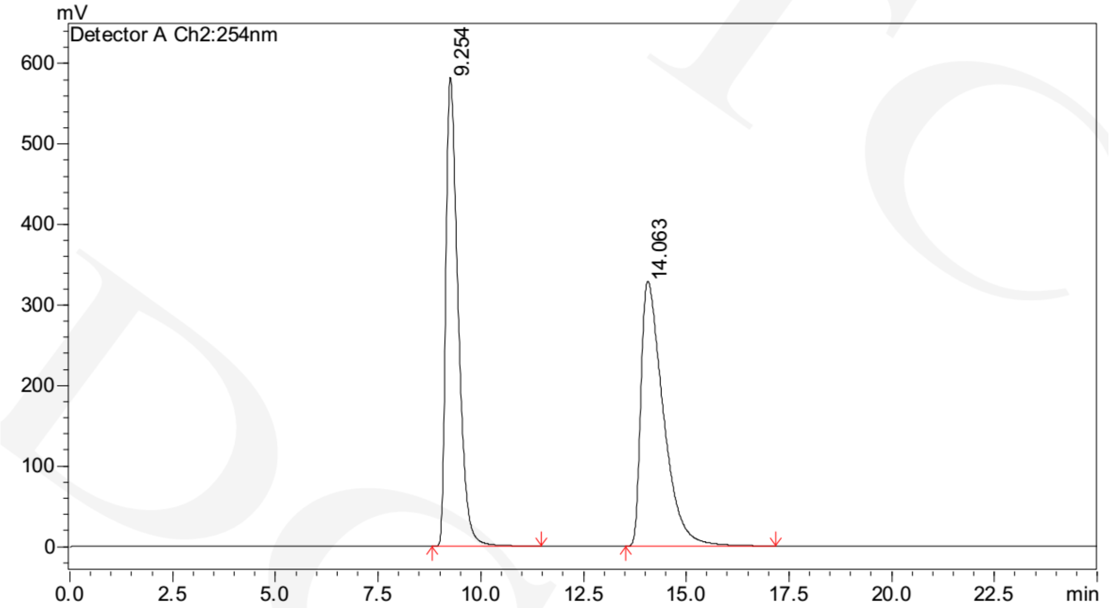

Peak#	Ret. Time	Area	Area%	T.Plate#	Tailing F.	Resolution
1	9.254	12161198	49.2951	4653.218	1.644	--
2	14.063	12509010	50.7049	3294.776	2.050	6.316

Chromatogram of racemic **S-1-011**

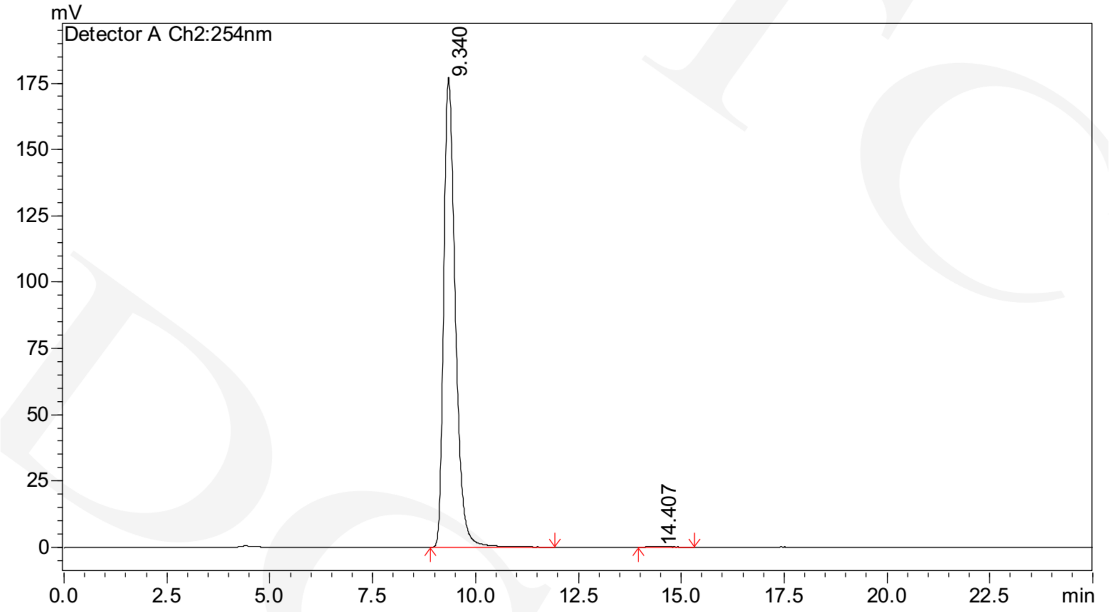

Peak#	Ret. Time	Area	Area%	T.Plate#	Tailing F.	Resolution
1	9.340	3482762	99.8337	5448.383	1.404	--
2	14.407	5801	0.1663	4694.278	1.610	7.522

Chromatogram of **S-1-011**-1

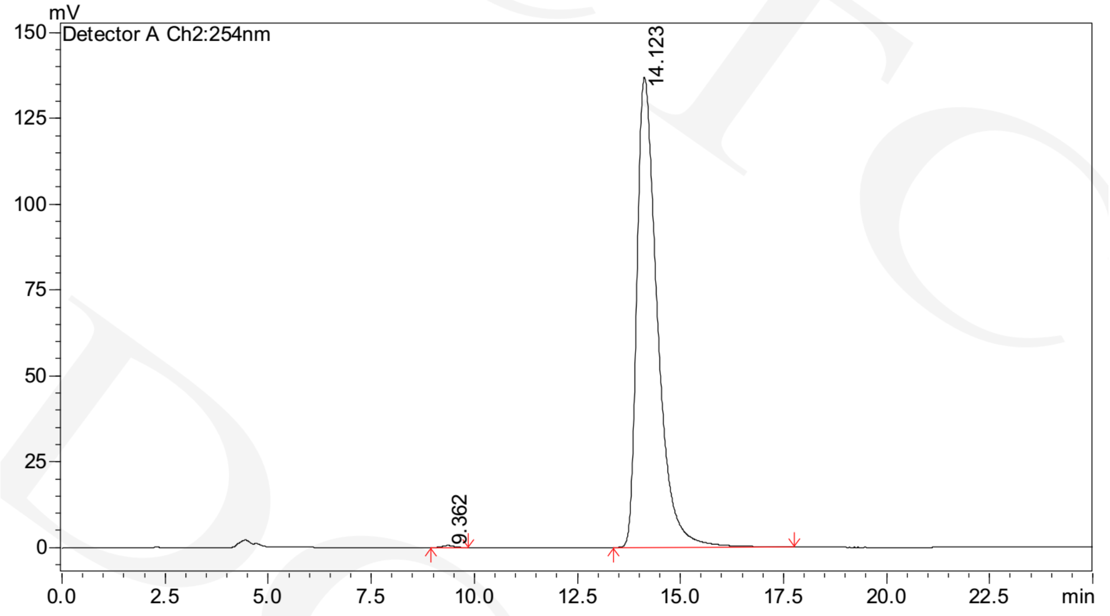

Peak#	Ret. Time	Area	Area%	T.Plate#	Tailing F.	Resolution
1	9.362	10814	0.2275	5843.085	1.141	--
2	14.123	4743378	99.7725	4181.857	1.652	6.983

Chromatogram of **S-1-011**-2

3.3 Characterization data of **(+)-JX21108** and **(-)-JX21108**

(+)-JX21108

Compound **(+)-JX21108** was synthesized according to the above general synthetic method.

232 mg (19% from **(+)-S-1-011**); $[\alpha]_{20}^{D}$ (deg mL^3 g^{-1} dm^{-1})=22.3 (c=0.01 g mL^{-3} in MeOH); ^{1}H NMR (400 MHz, DMSO-d_6) δ 11.50–11.22 (m, 1H), 8.70 (m, 2H), 8.53 (s, 1H), 7.87 (s, 1H), 7.70 (d, J = 8.5 Hz, 1H), 7.58 (d, J = 8.5 Hz, 1H), 4.57 (m, 2H), 3.88 (s, 3H), 3.66 (m, 1H), 3.62–3.48 (m, 4H), 3.42 (m, 1H), 3.34 (m, 1H), 3.24 (m, 1H), 2.19–1.93 (m, 4H); ^{13}C NMR (151 MHz, DMSO) δ 161.90, 159.78, 157.05, 156.86, 137.97, 134.92, 134.90, 127.14, 127.11, 125.17, 124.36, 120.53, 114.39, 111.59, 111.57, 104.75, 104.72, 101.75, 59.71, 59.19, 56.59, 56.23, 52.06, 52.03, 47.64, 47.48, 47.18, 47.14, 45.76, 45.72, 35.56, 35.26, 33.86, 33.46, 33.05; HRMS (ESI) m/z calcd. for $C_{23}H_{26}N_7O_2$ [M+H]$^+$ 432.2148, found 432.2149; Purity: 99% (254 nm, RT = 4.043 min).

(-)-JX21108

Compound **(-)-JX21108** was synthesized according to the above general synthetic method.

226 mg (18% from **(-)-S-1-011**); $[\alpha]_{20}^{D}$ (deg mL^3 g^{-1} dm^{-1})=-23.8 (c=0.01 g mL^{-3} in MeOH); ^{1}H NMR (400 MHz, DMSO-d_6) δ 11.46–11.19 (m, 1H), 8.69 (m, 2H), 8.53 (s, 1H), 7.87 (s, 1H), 7.70 (d, J = 8.5 Hz, 1H), 7.58 (d, J = 8.5 Hz, 1H), 4.57 (m, 2H), 3.88 (s, 3H), 3.65 (m, 1H), 3.55 (m, 4H), 3.42 (m, 1H), 3.34 (m, 1H), 3.29–3.19 (m, 1H), 2.19–1.93 (m, 4H); ^{13}C NMR (151 MHz, DMSO-d_6) δ 161.92, 159.84, 157.05, 156.74, 137.97, 134.92, 134.87, 127.14, 127.11, 125.17, 124.36, 120.53, 114.40, 111.59, 104.75, 104.72, 101.75, 59.72, 59.20, 56.58, 56.23, 52.06, 52.03, 47.64, 47.49, 47.18, 47.14, 45.75, 45.70, 35.56, 35.26, 33.86, 33.46, 33.05; HRMS (ESI) m/z calcd. for $C_{23}H_{26}N_7O_2$ [M+H]$^+$ 432.2148, found 432.2149; Purity: 99% (254 nm, RT = 4.084 min).

Reference

1 Dickens, J. W. J. *et al.* New mono-hydrochloric salt of N-hydroxy-2-(4-((((1-methyl-1H-indol-3-yl)methyl)amino)methyl)-1-piperidinyl)-5-pyrimidinecarboxamide useful in a medicament for treating e.g. leukemia, lung cancer, pancreatic cancer, prostate cancer. WO2008138918-A1.

2 Verdonck, M. G. C. *et al.* New substituted indolyl alkyl amino derivatives useful for treating e.g. cancer, osteoarthritis, conjunctivitis, asthma, Adult respiratory distress syndrome and diabetes. WO2006010750-A1.

4. Data of characterization

Compound **JX21011** ^{1}H NMR

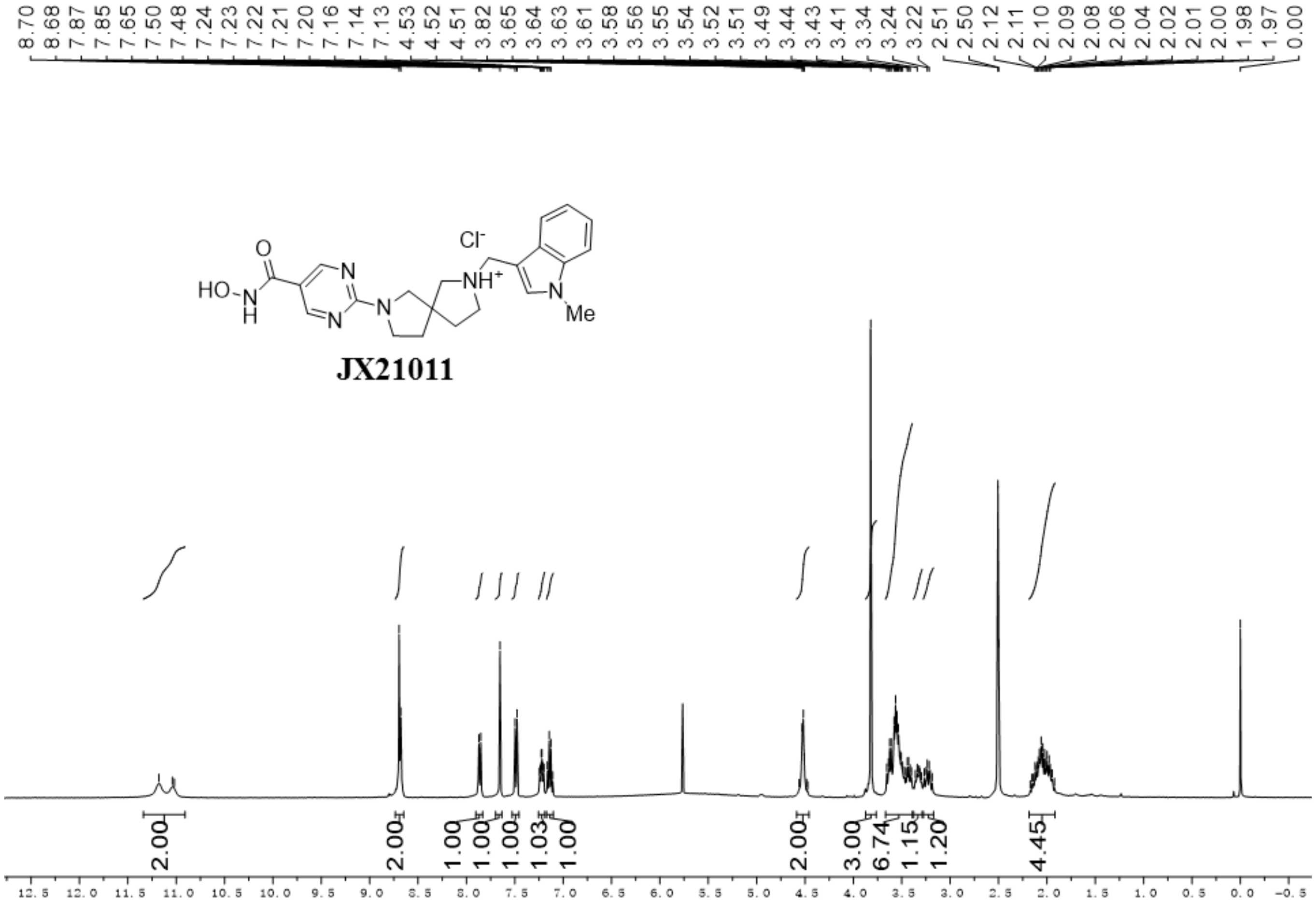

JX21011

Compound **JX21011** ^{13}C NMR

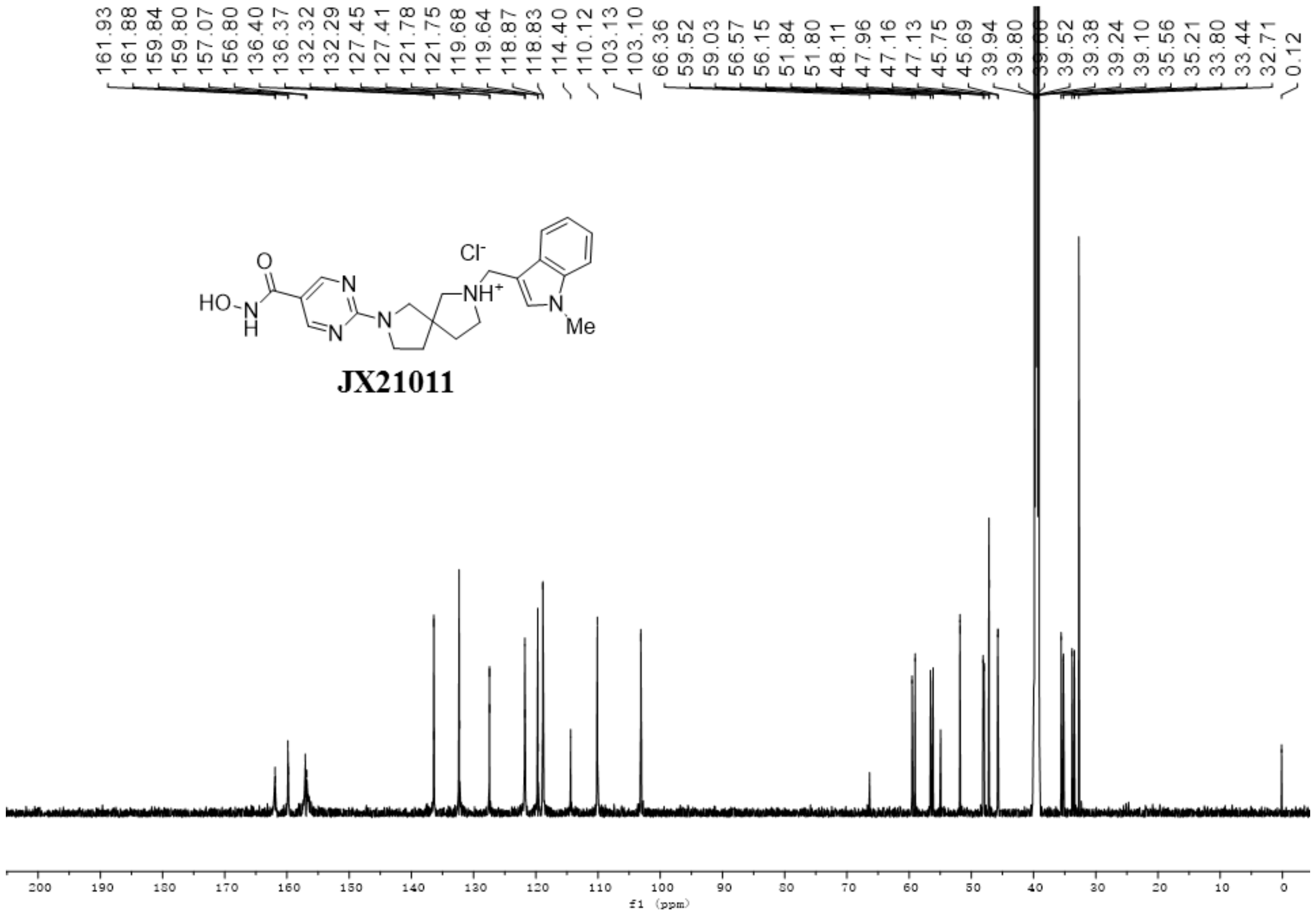

Compound **JX21011** HRMS

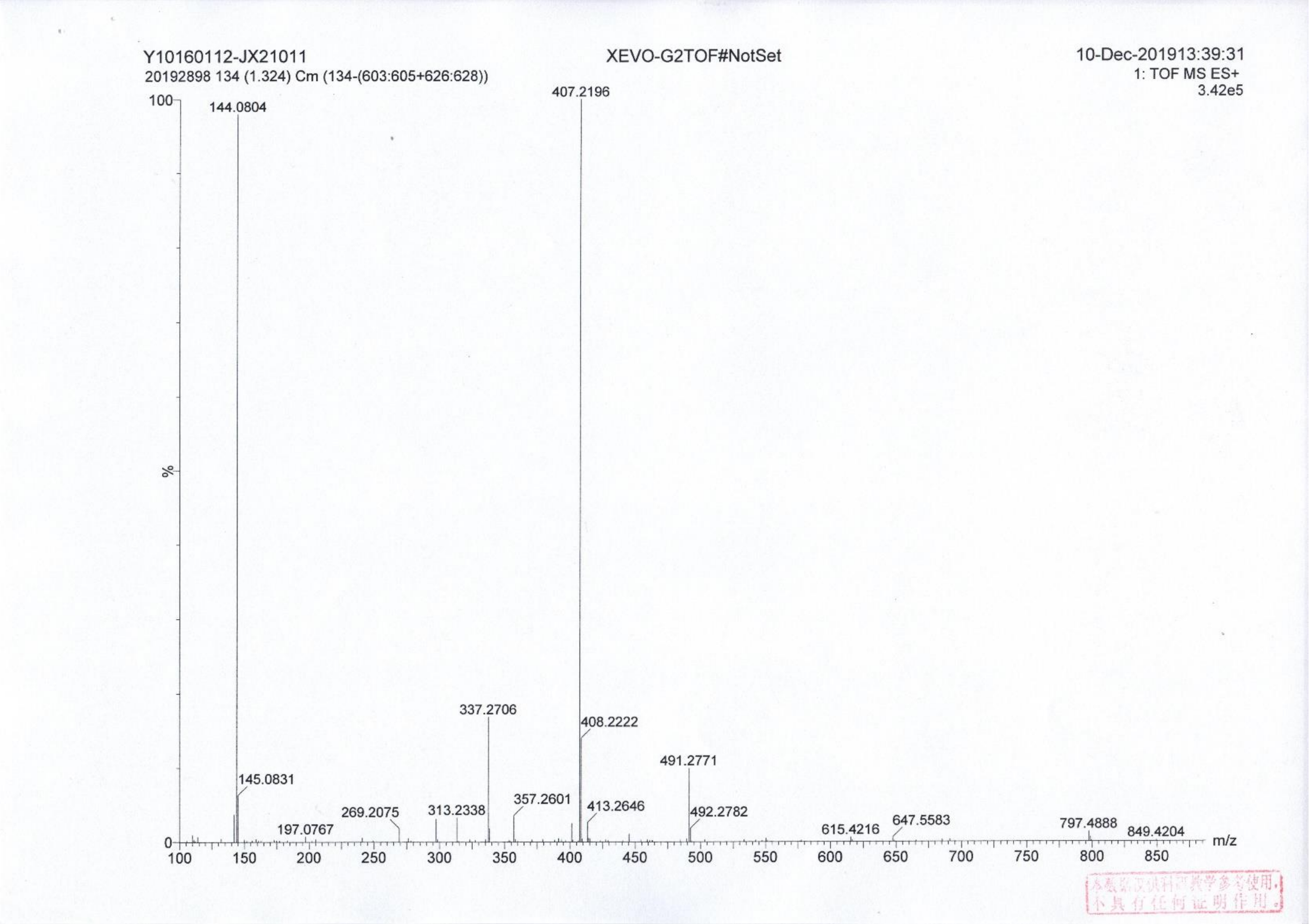

Compound **JX21011** HPLC

数据文件: C:\CHEM32\1\DATA\LRX\JX21011-2-A0340.D
样品名称: JX21011-2-A

===

操作者　　　　: LRX
仪器　　　　　: 仪器 1　　　　　　　　　　位置 : 样品瓶 1
进样日期　　　: 2019/12/4 20:55:59
采集方法　　　: C:\CHEM32\1\METHODS\LYL_LC.M
最后修改　　　: 2019/12/4 20:53:59 : LRX
(调用后修改)
分析方法　　　: C:\CHEM32\1\METHODS\ZC_LC.M
最后修改　　　: 2019/12/29 22:55:18 : ZC
样品信息　　　: MeOH:H3PO4(c=0.1)=90:10, 0.5mL/min

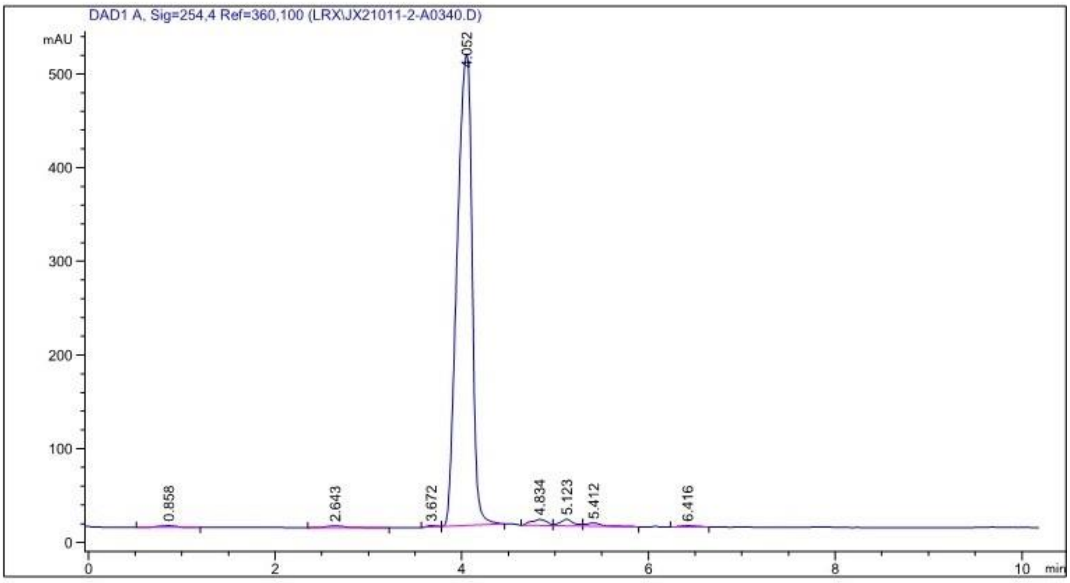

===

面积百分比报告

===

排序　　　　　　: 信号
乘积因子:　　　　: 1.0000
稀释因子:　　　　: 1.0000
样品量:　　　　　: 10.00000 [ng/ul] (校正中没有使用)
内标使用乘积因子和稀释因子

信号 1: DAD1 A, Sig=254,4 Ref=360,100

峰 #	保留时间 [min]	类型	峰宽 [min]	峰面积 [mAU*s]	峰高 [mAU]	峰面积 %
1	0.858	BB	0.2025	25.66597	1.91384	0.4412
2	2.643	BB	0.2134	24.22771	1.68889	0.4165
3	3.672	BV	0.1022	12.79082	1.90737	0.2199
4	4.052	VB	0.1831	5559.62500	502.52350	95.5792
5	4.834	BV	0.1750	72.59579	6.36809	1.2480
6	5.123	VV	0.1356	67.24068	7.03036	1.1560

Compound **JX21108** ^{1}H NMR

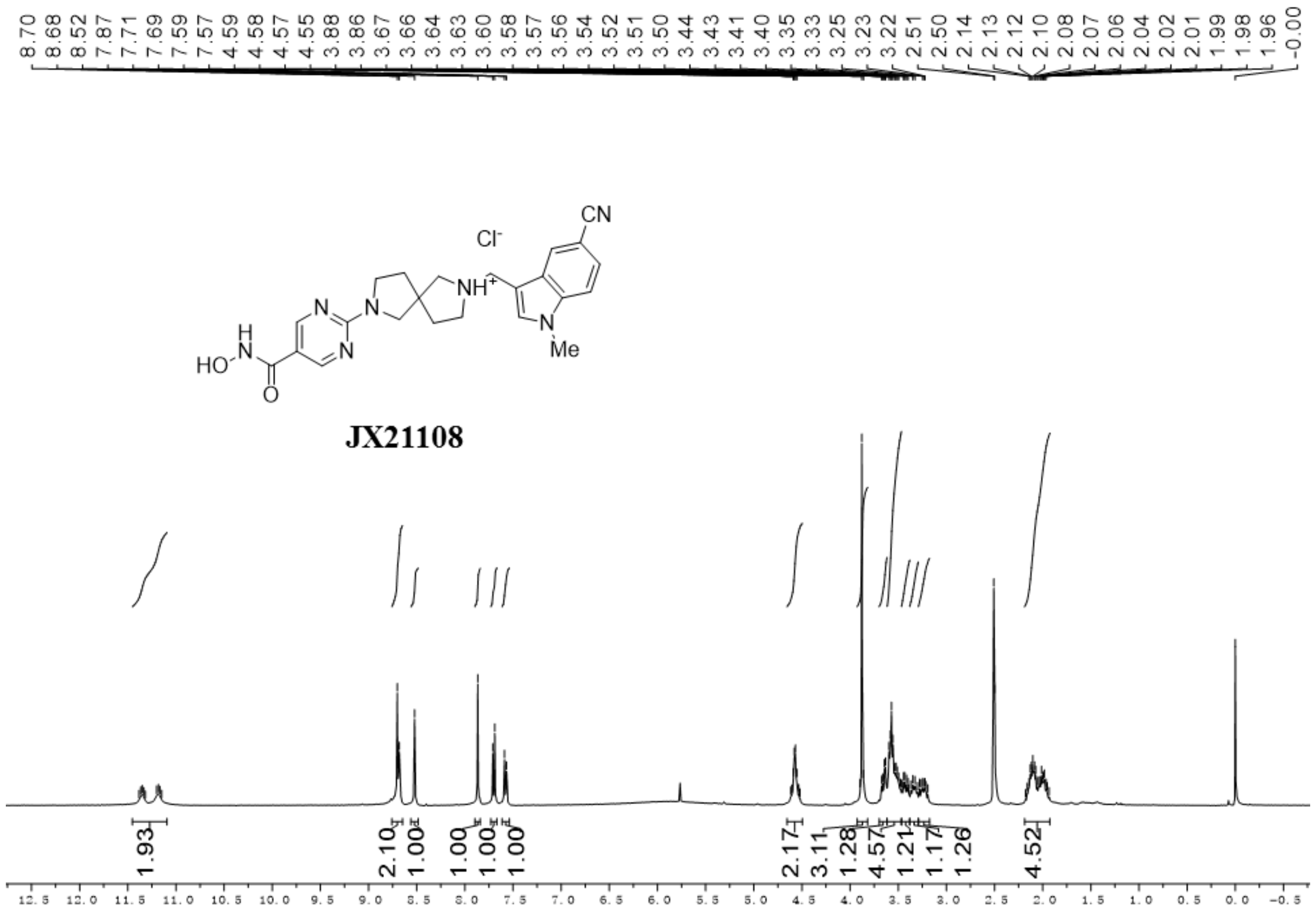

Compound **JX21108** ^{13}C NMR

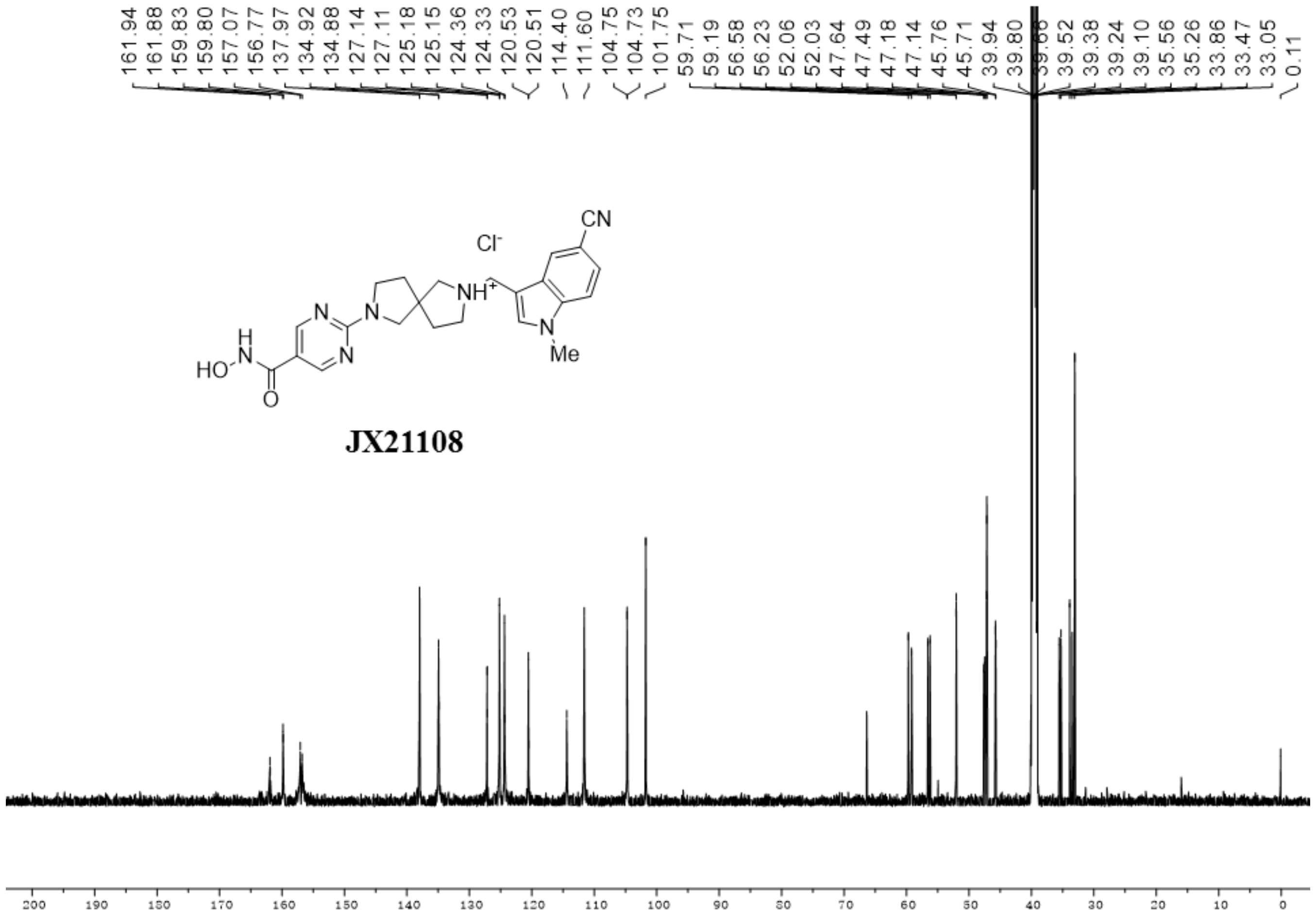

Compound **JX21108** HRMS

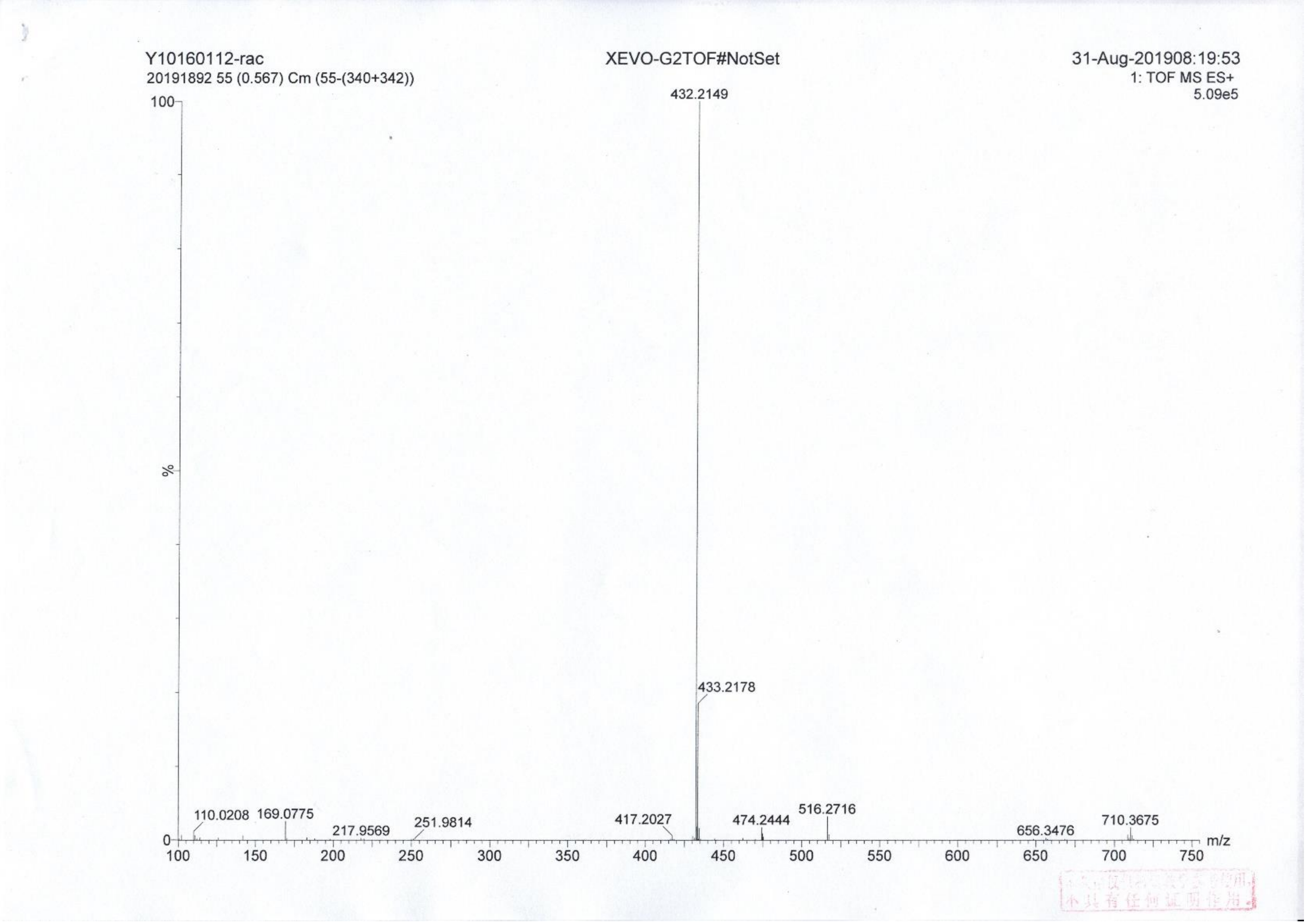

Compound **JX21108** HPLC

数据文件: C:\CHEM32\1\DATA\LRX\JX21108-1-A0379.D
样品名称: JX21108-1-A

===

操作者	: LRX		
仪器	: 仪器 1	位置	: 样品瓶 1
进样日期	: 2019/12/5 19:07:35		
采集方法	: C:\CHEM32\1\METHODS\LYL_LC.M		
最后修改	: 2019/12/5 17:46:18 : LRX		
分析方法	: C:\CHEM32\1\METHODS\ZC_LC.M		
最后修改	: 2019/12/29 22:55:18 : ZC		
样品信息	: MeOH:H3PO4(c=0.1)=90:10, 0.5mL/min		

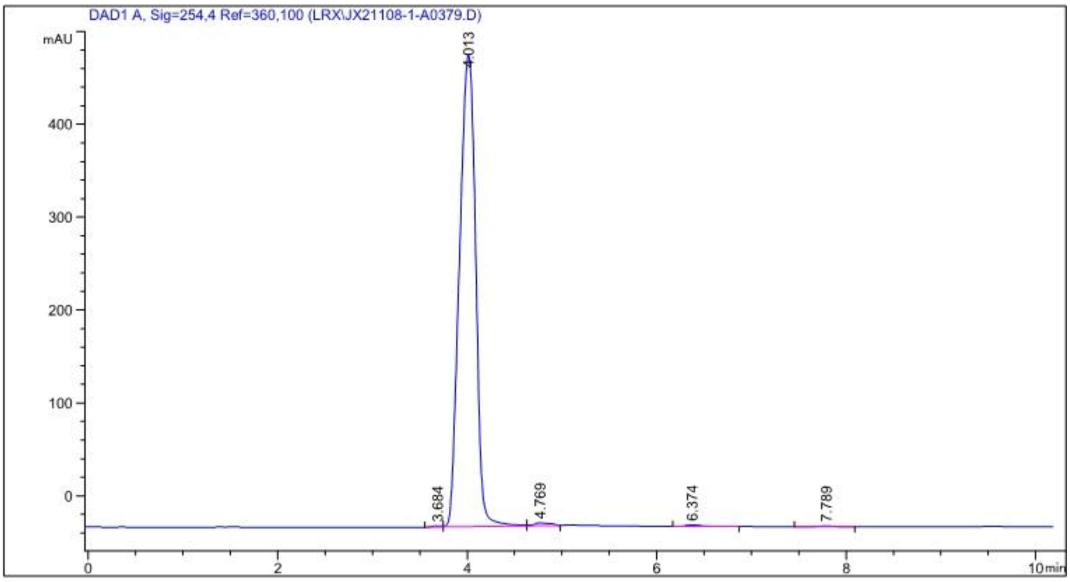

===
面积百分比报告
===

排序 : 信号
乘积因子: : 1.0000
稀释因子: : 1.0000
样品量: : 10.00000 [ng/ul] (校正中没有使用)
内标使用乘积因子和稀释因子

信号 1: DAD1 A, Sig=254,4 Ref=360,100

峰 #	保留时间 [min]	类型	峰宽 [min]	峰面积 [mAU*s]	峰高 [mAU]	峰面积 %
1	3.684	BV	0.1025	7.89031	1.17299	0.1296
2	4.013	VV	0.1958	5999.80420	508.11118	98.5542
3	4.769	VB	0.1701	40.93482	3.31476	0.6724
4	6.374	BB	0.1953	25.16251	1.82020	0.4133
5	7.789	BB	0.2190	14.02817	1.02924	0.2304
总量 :				6087.82001	515.44837	

Compound **(+)-JX21108** ^{1}H NMR

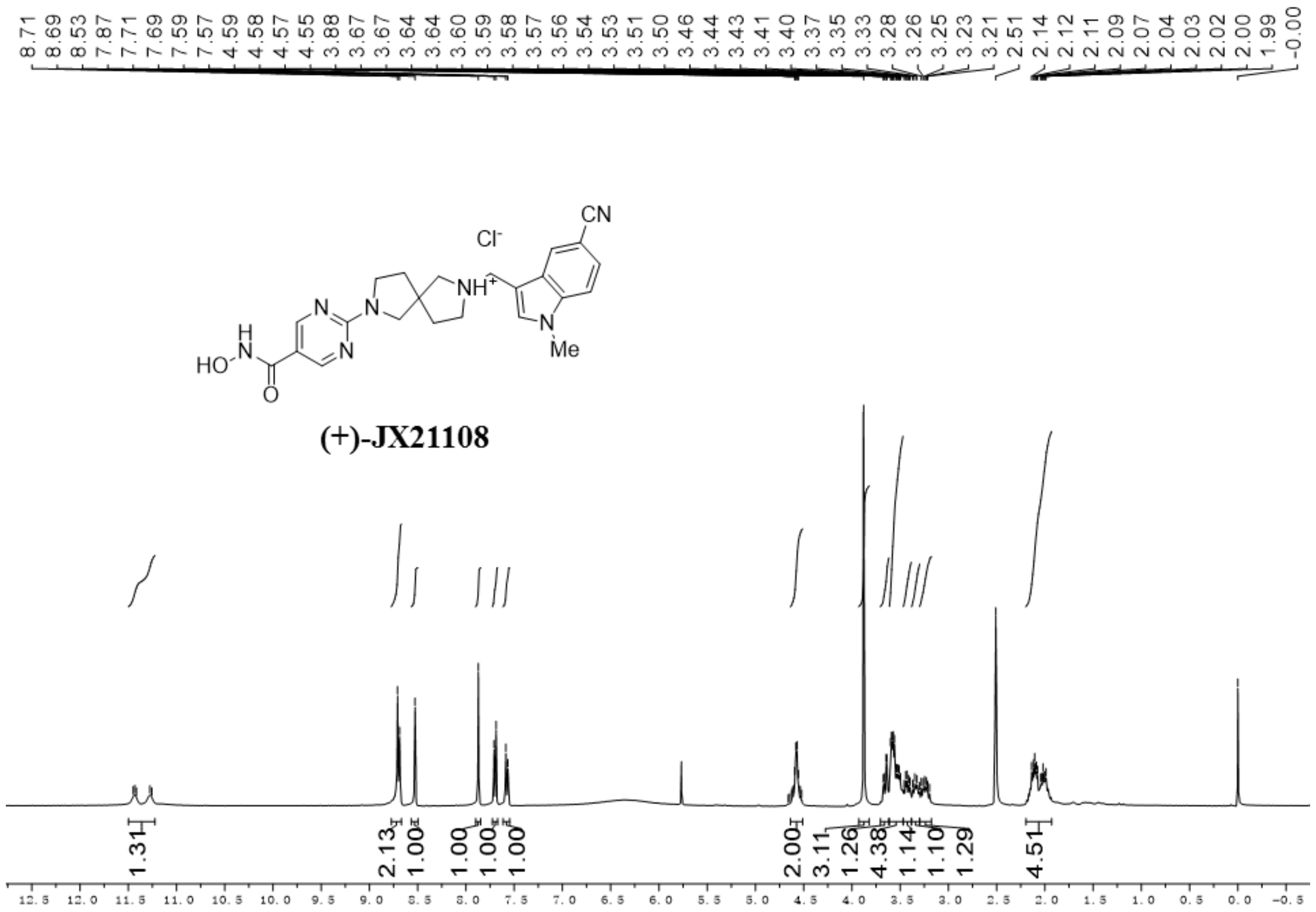

Compound **(+)-JX21108** ^{13}C NMR

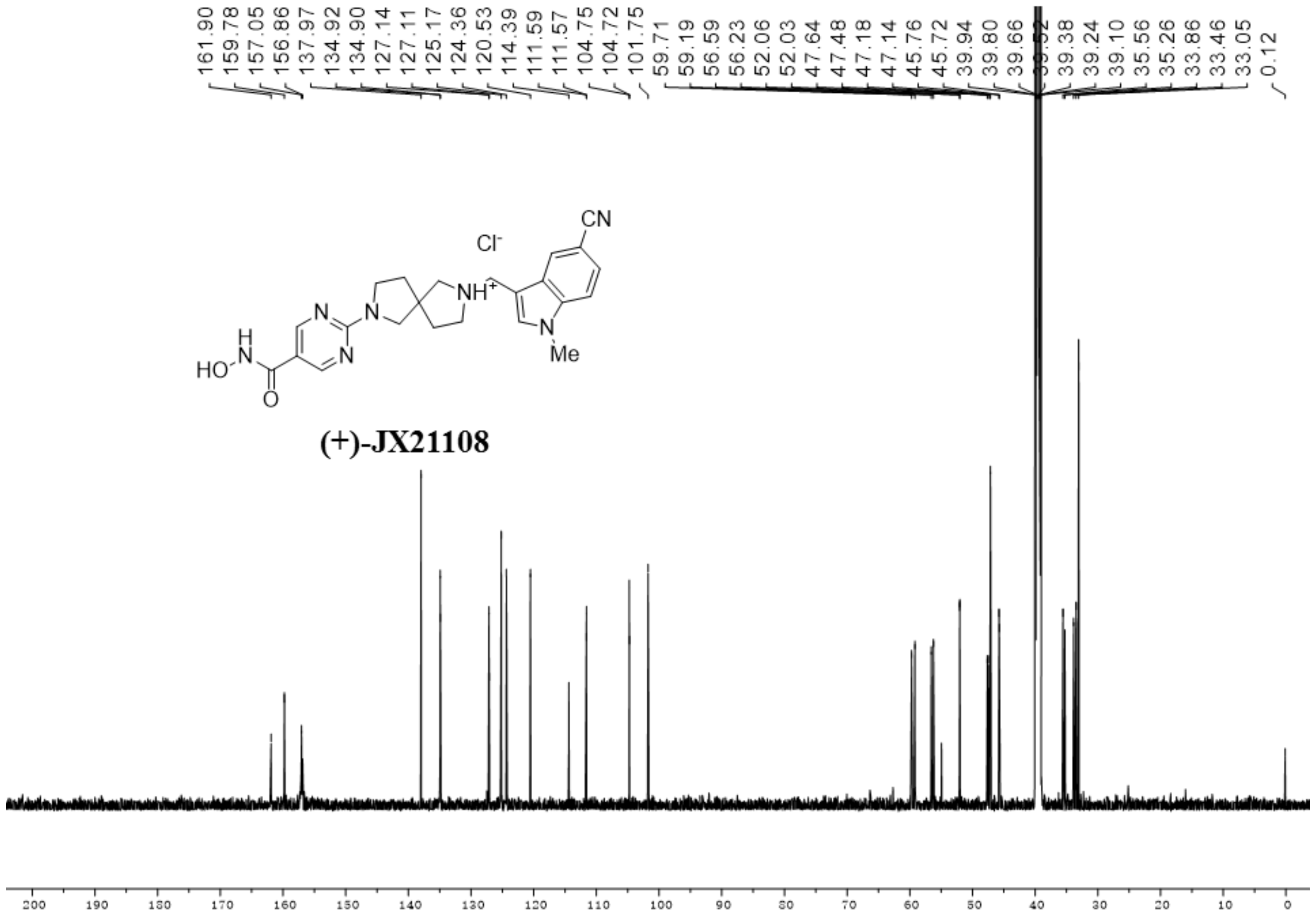

Compound **(+)-JX21108** HRMS

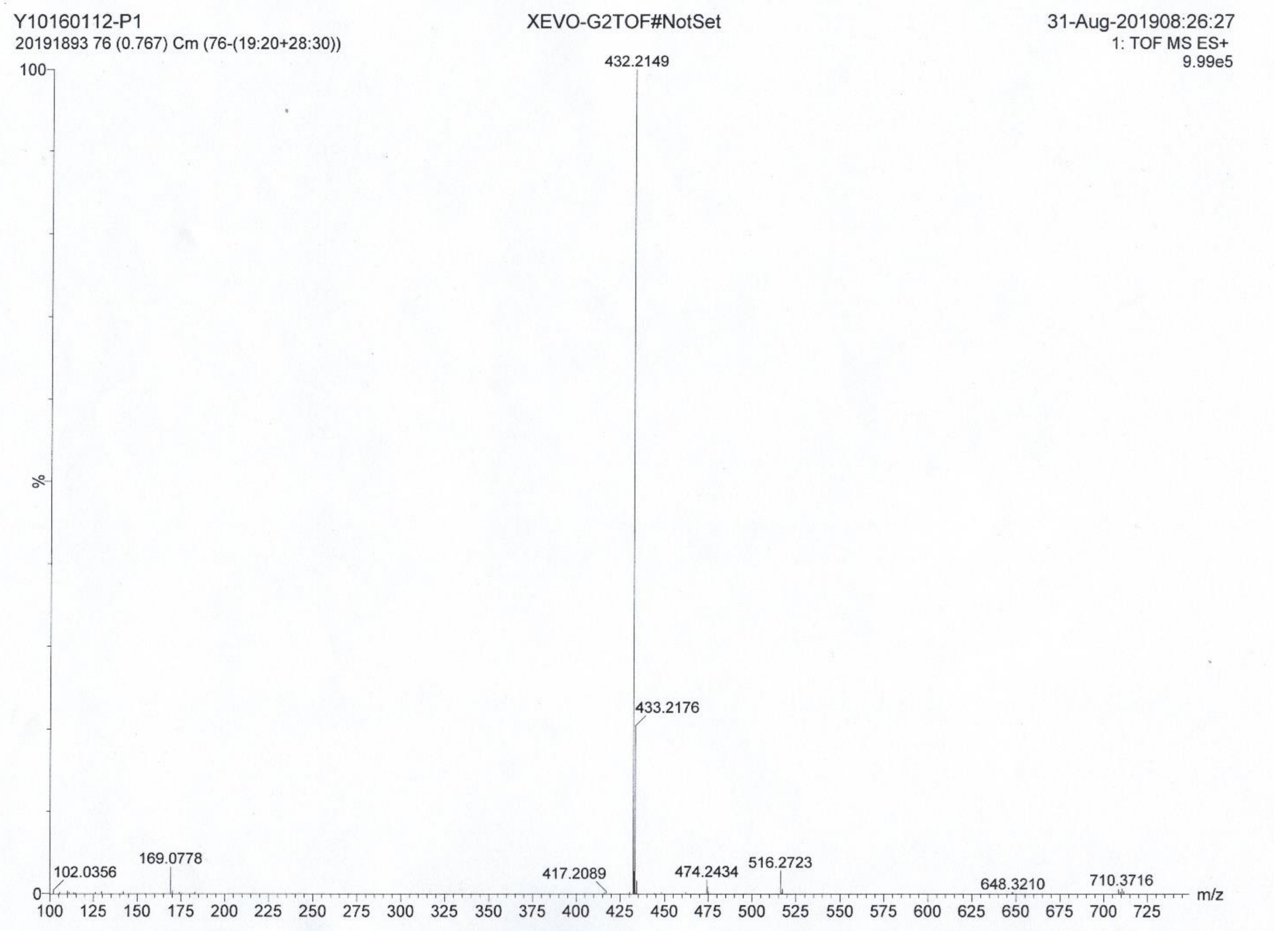

Compound **(+)-JX21108** HPLC

数据文件: C:\CHEM32\1\DATA\LRX\108-1-1-A000380.D
样品名称: 108-1-1-A

===

操作者 : LRX
仪器 : 仪器 1 位置 : 样品瓶 1
进样日期 : 2019/12/5 19:23:11
采集方法 : C:\CHEM32\1\METHODS\LYL_LC.M
最后修改 : 2019/12/5 17:46:18 : LRX
分析方法 : C:\CHEM32\1\METHODS\ZC_LC.M
最后修改 : 2019/12/29 22:55:18 : ZC
样品信息 : MeOH:H3PO4(c=0.1)=90:10, 0.5mL/min

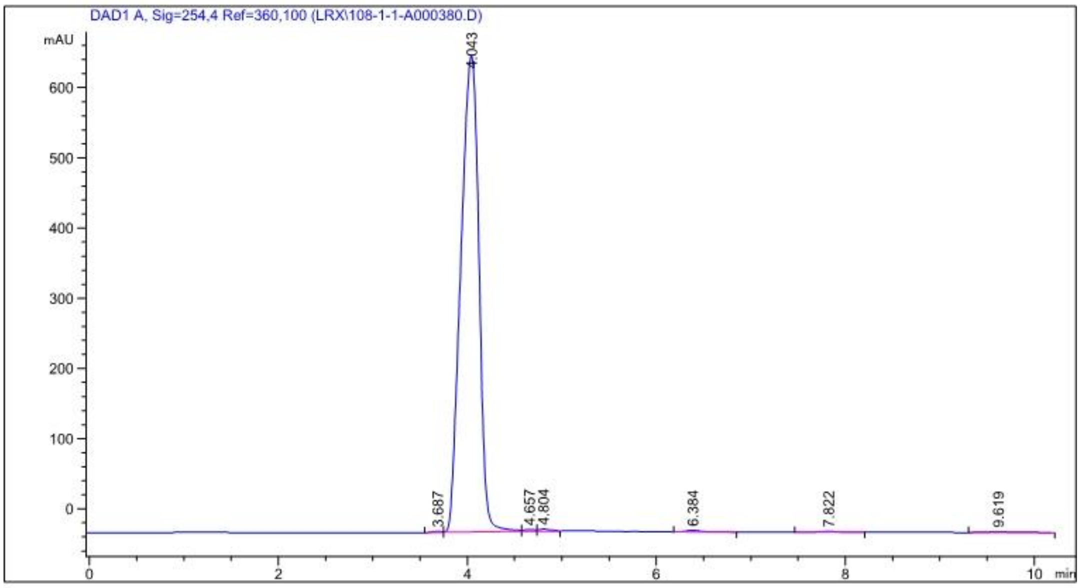

===

面积百分比报告

===

排序 : 信号
乘积因子: : 1.0000
稀释因子: : 1.0000
样品量: : 10.00000 [ng/ul] (校正中没有使用)
内标使用乘积因子和稀释因子

信号 1: DAD1 A, Sig=254,4 Ref=360,100

峰 #	保留时间 [min]	类型	峰宽 [min]	峰面积 [mAU*s]	峰高 [mAU]	峰面积 %
1	3.687	BV	0.1089	9.35873	1.38457	0.1027
2	4.043	VV	0.2184	8983.01855	678.78870	98.6046
3	4.657	VV	0.1184	23.74902	2.87441	0.2607
4	4.804	VB	0.1370	28.70254	3.07344	0.3151
5	6.384	BB	0.1866	29.12960	2.25600	0.3197
6	7.822	BB	0.2406	19.96937	1.33468	0.2192
7	9.619	BB	0.2268	16.21591	1.09510	0.1780

Compound **(-)-JX21108** ^{1}H NMR

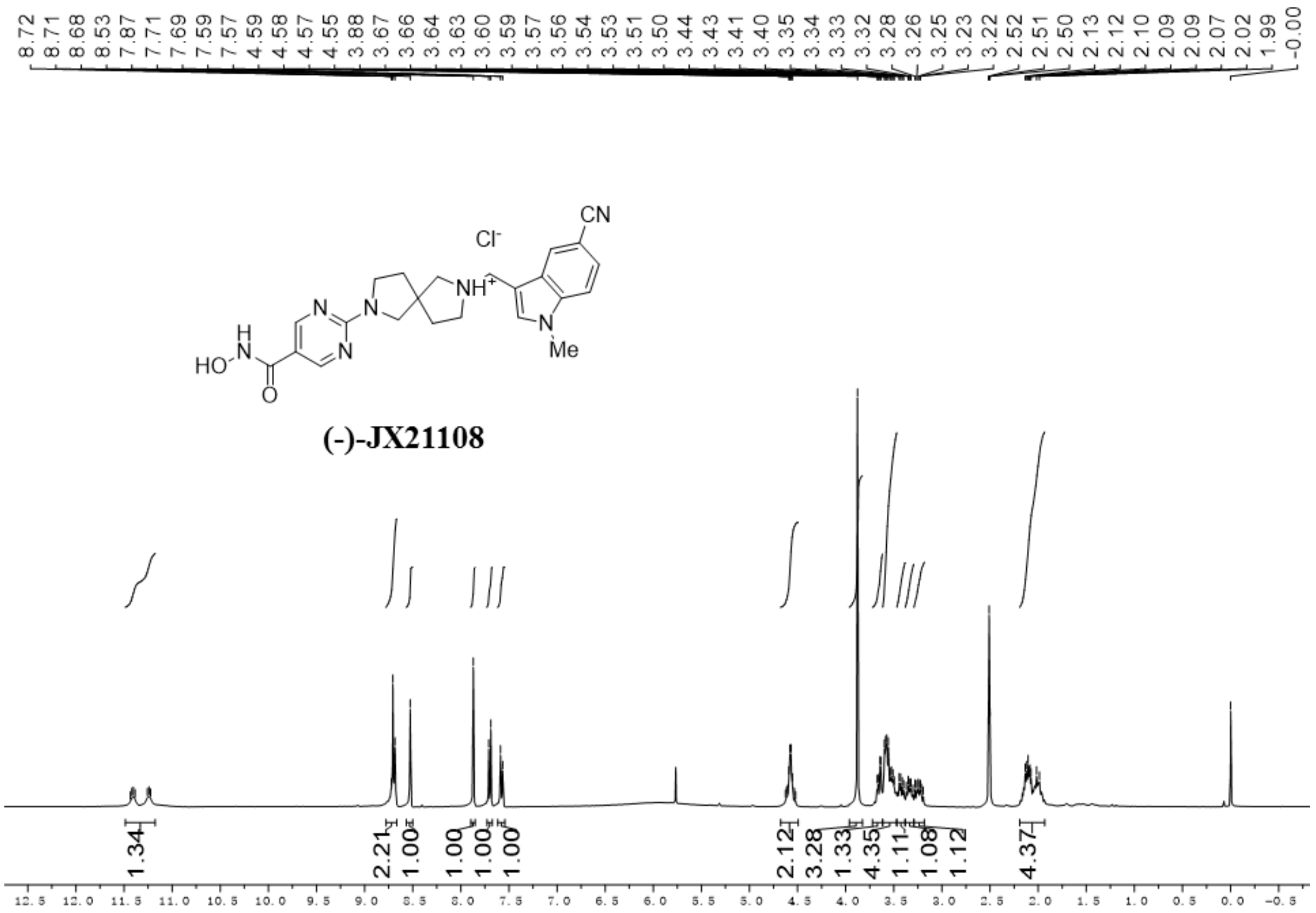

Compound **(-)-JX21108** ^{13}C NMR

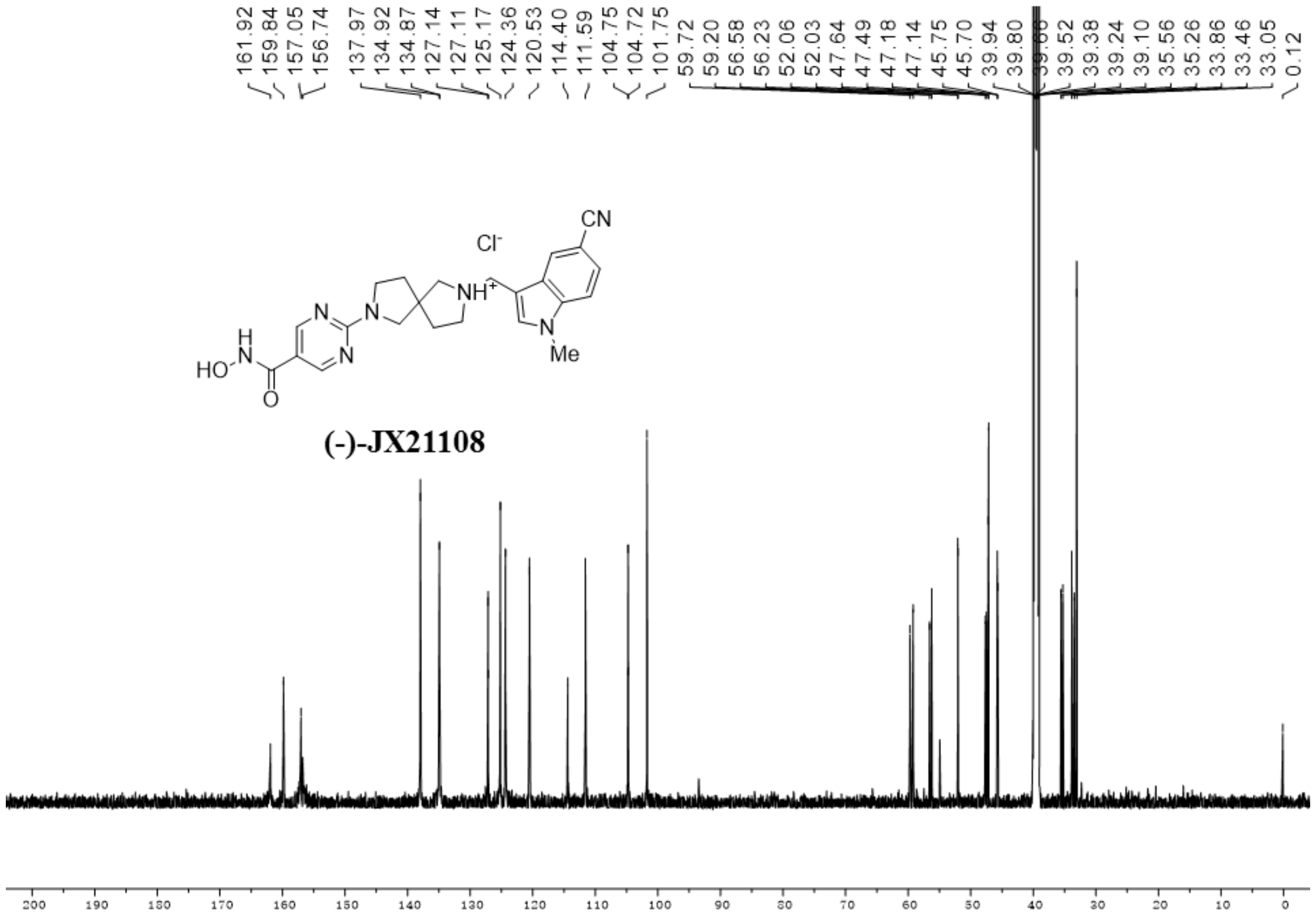

Compound **(+)-JX21108** HRMS

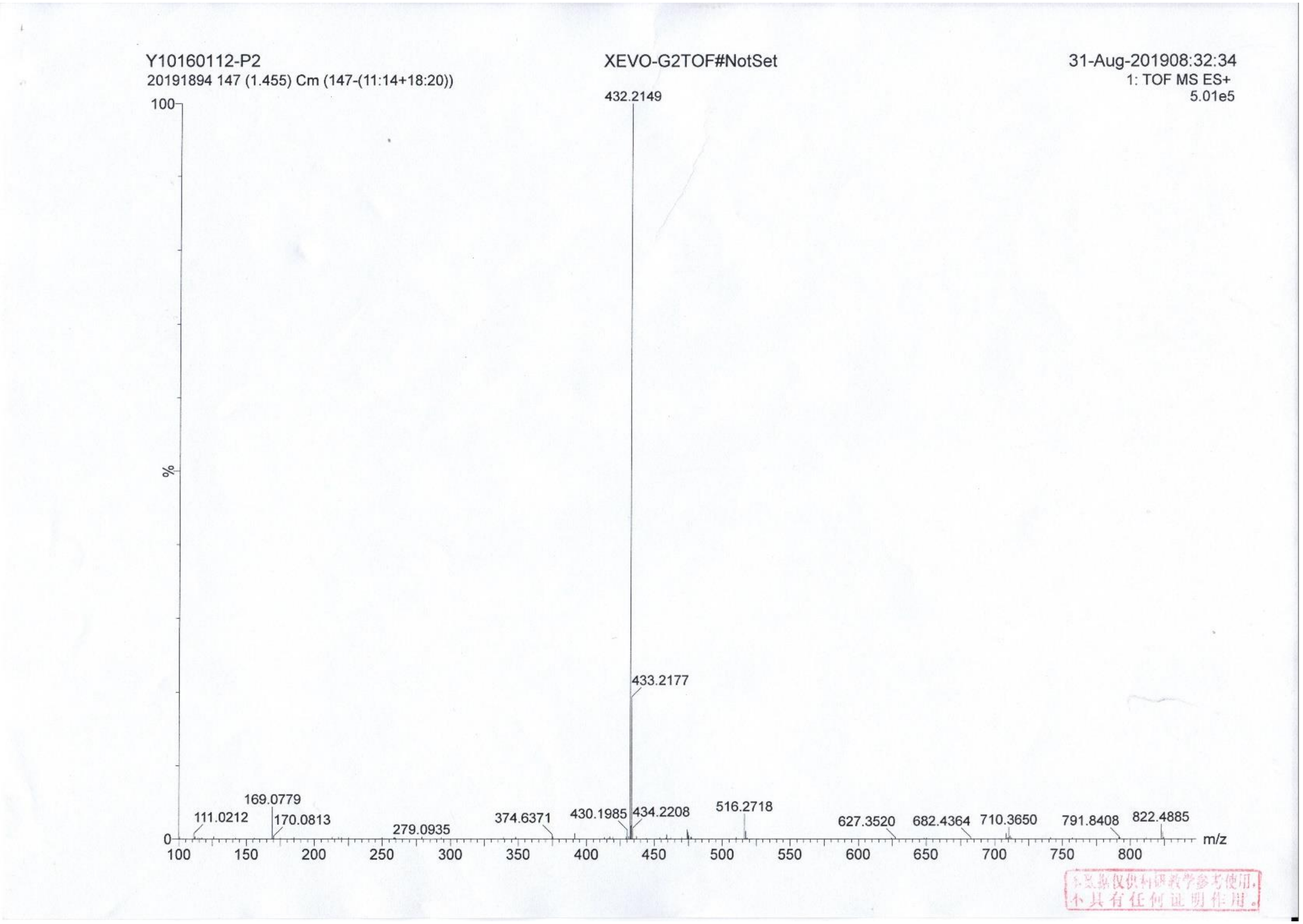

Compound **(-)-JX21108** HPLC

数据文件: C:\CHEM32\1\DATA\LRX\108-2-1-A000381.D
样品名称: 108-2-1-A

===

操作者	: LRX		
仪器	: 仪器 1	位置 :	样品瓶 1
进样日期	: 2019/12/5 19:37:33		
采集方法	: C:\CHEM32\1\METHODS\LYL_LC.M		
最后修改	: 2019/12/5 17:46:18 : LRX		
分析方法	: C:\CHEM32\1\METHODS\ZC_LC.M		
最后修改	: 2019/12/29 22:55:18 : ZC		
样品信息	: MeOH:H3PO4(c=0.1)=90:10, 0.5mL/min		

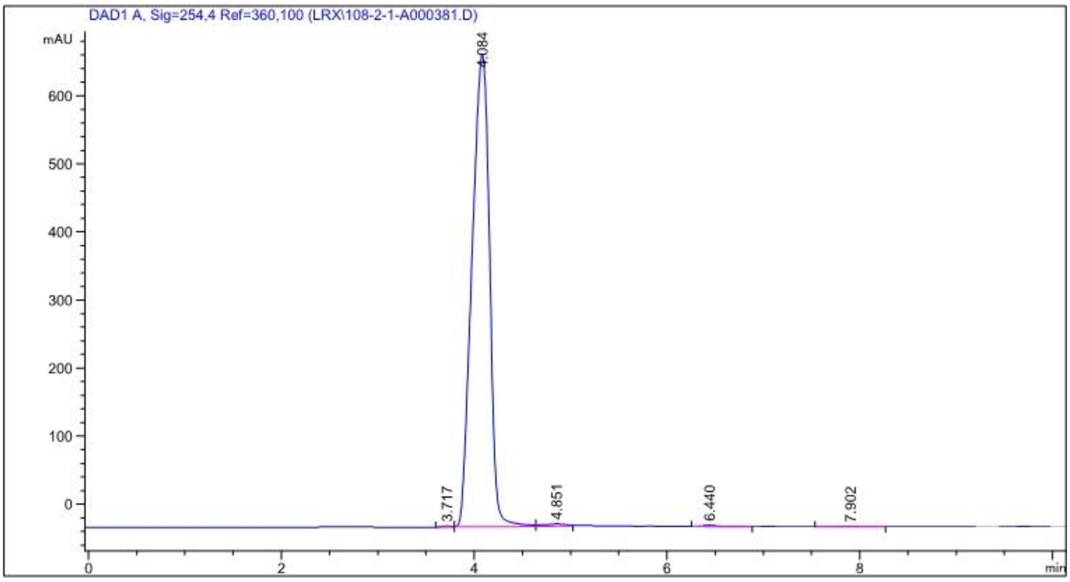

===

面积百分比报告

===

排序	:	信号		
乘积因子:	:	1.0000		
稀释因子:	:	1.0000		
样品量:	:	10.00000	[ng/ul]	(校正中没有使用)

内标使用乘积因子和稀释因子

信号 1: DAD1 A, Sig=254,4 Ref=360,100

峰 #	保留时间 [min]	类型	峰宽 [min]	峰面积 [mAU*s]	峰高 [mAU]	峰面积 %
1	3.717	BV	0.1044	9.79344	1.45804	0.1107
2	4.084	VV	0.2070	8739.78125	693.66473	98.7993
3	4.851	VV	0.2038	56.18254	3.52836	0.6351
4	6.440	BB	0.1702	23.14072	1.98251	0.2616
5	7.902	BB	0.2469	17.10129	1.11498	0.1933

总量 : 8845.99923 701.74863
